# Supplementary material for: The Plasmodium falciparum apicoplast cysteine desulfurase provides sulfur for both iron-sulfur cluster assembly and tRNA modification
Source: eLife. 2023 May 11;12:e84491. doi: 10.7554/eLife.84491 (PMC10219651; doi:10.7554/eLife.84491)
Supplement: Figure 1—source data 1. [file elife-84491-fig1-data1.zip › Figure 1- source data 1/Figure 1- source data 1.pdf]

Figure 1(B), Top

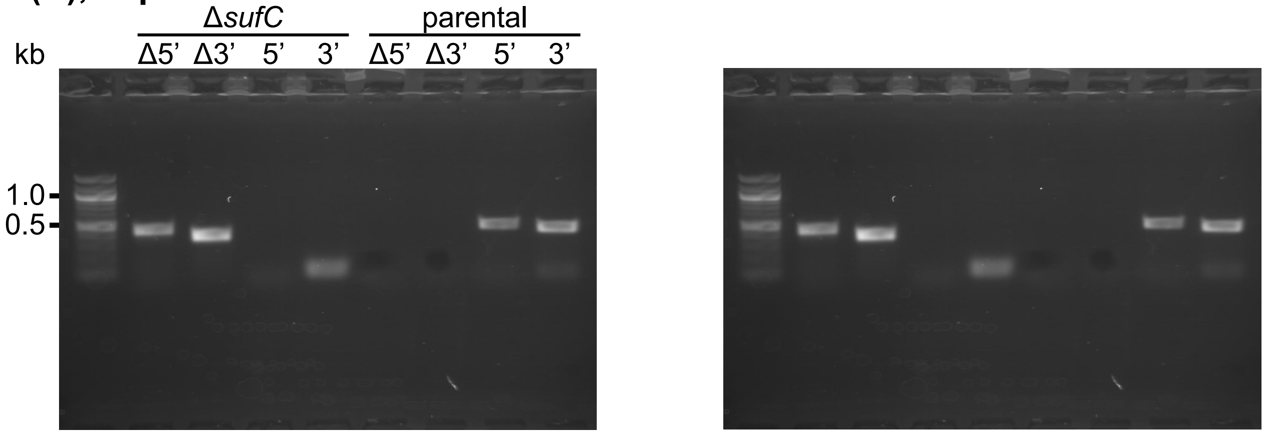

Figure 1(B), Bottom

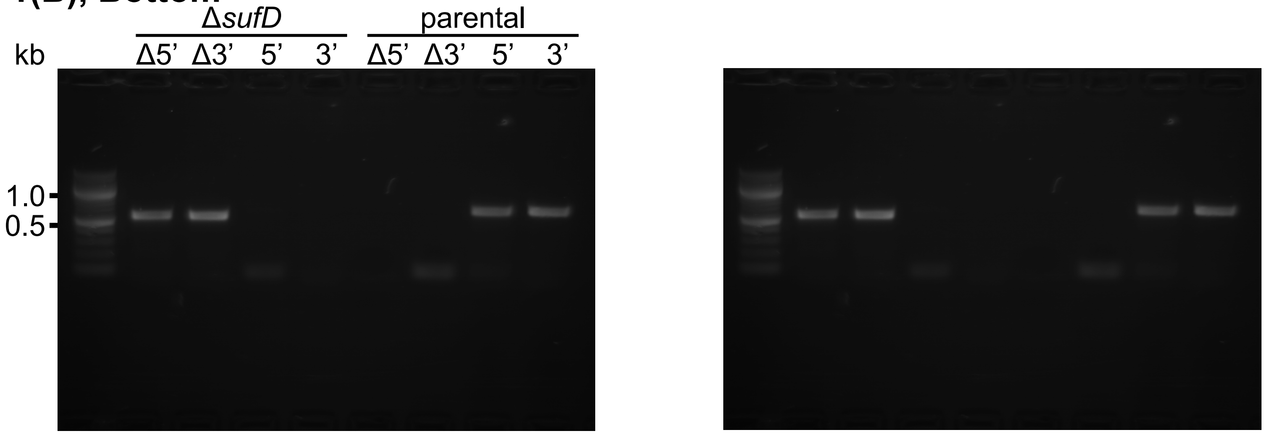

Figure 1(C), Left

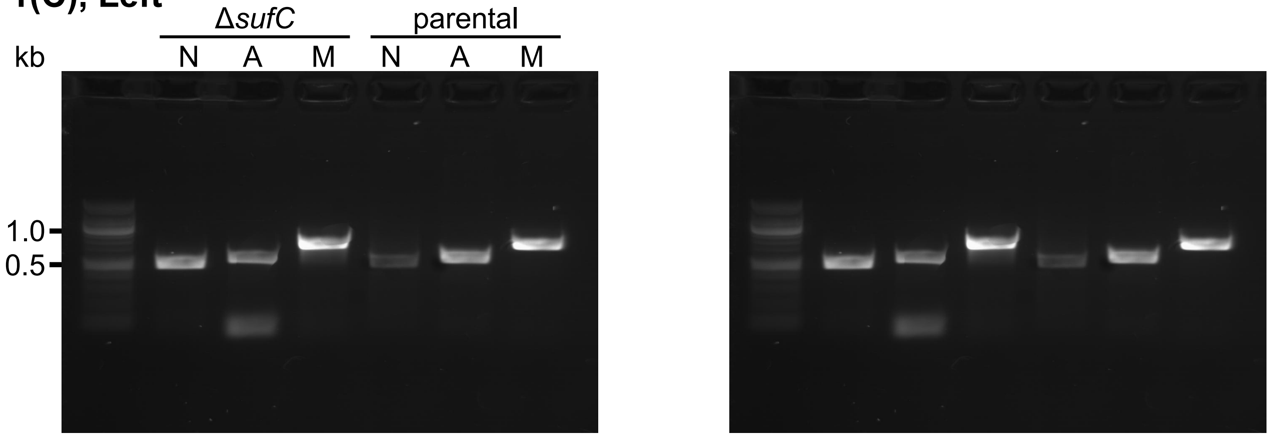

Figure 1(C), Right

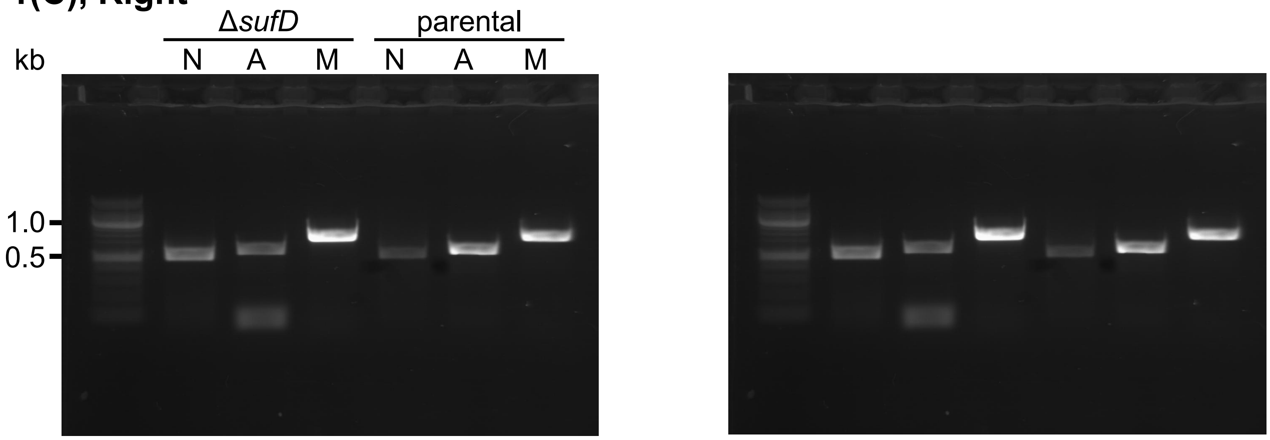

Figure 1(F), Top

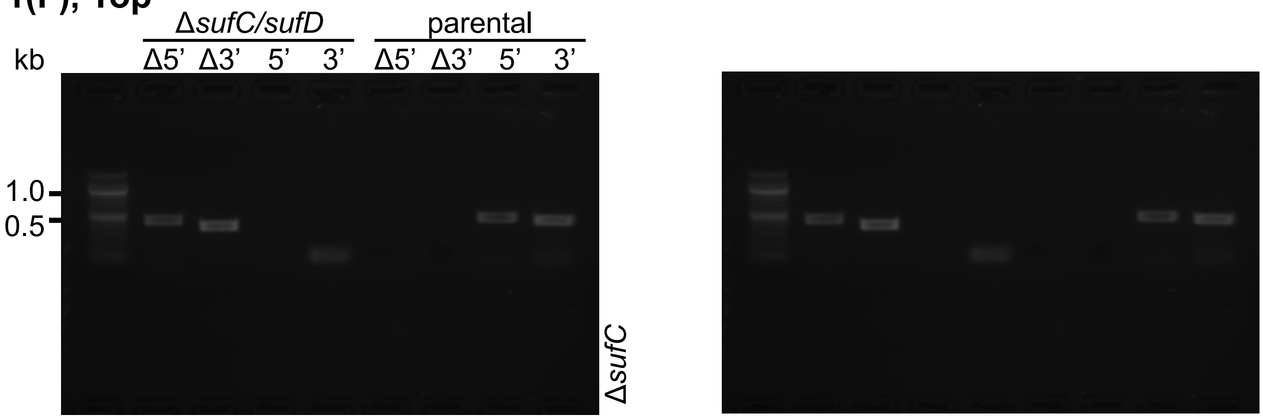

Figure 1(F), Bottom

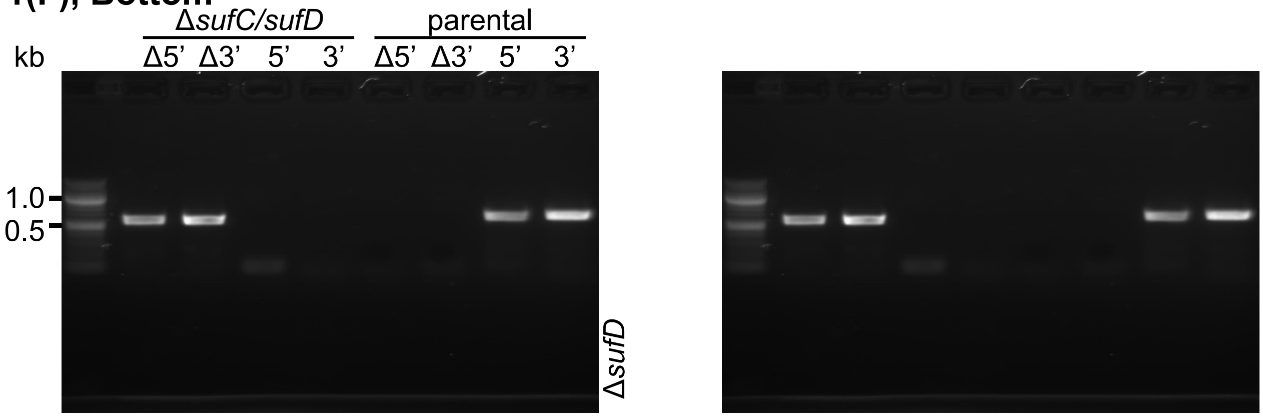

Figure 1(G)

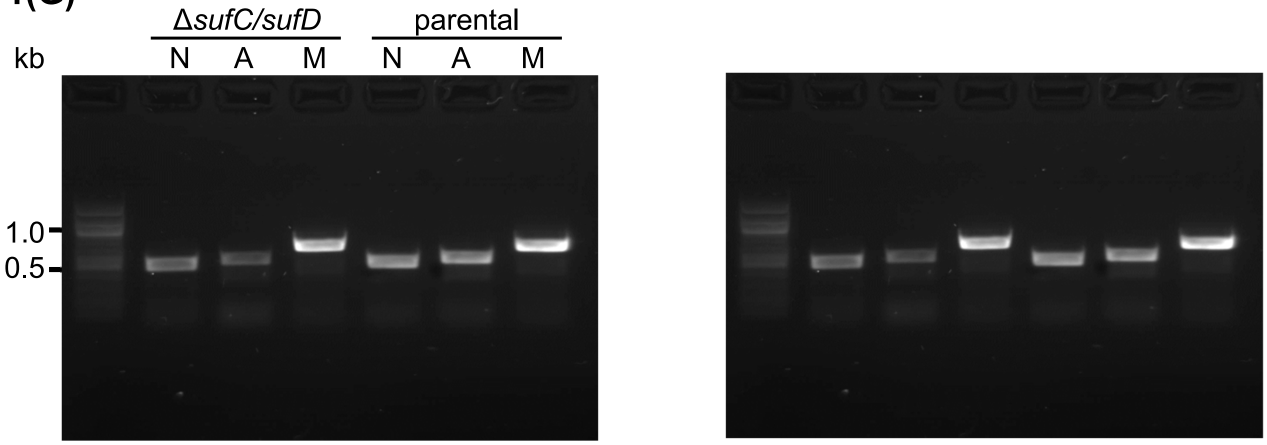

Figure 1- source data 1. Uncropped agarose gel images of PCR analyses presented in **Figures 1(B)**, **1(C)**, **1(F)**, and **1(G)**.
