# Supplementary material for: The Plasmodium falciparum apicoplast cysteine desulfurase provides sulfur for both iron-sulfur cluster assembly and tRNA modification
Source: eLife. 2023 May 11;12:e84491. doi: 10.7554/eLife.84491 (PMC10219651; doi:10.7554/eLife.84491)
Supplement: Figure 1—source data 1. [file elife-84491-fig1-data1.zip › Figure 1- source data 1/Figure 1- source data 1.pptx]

## Slide 1
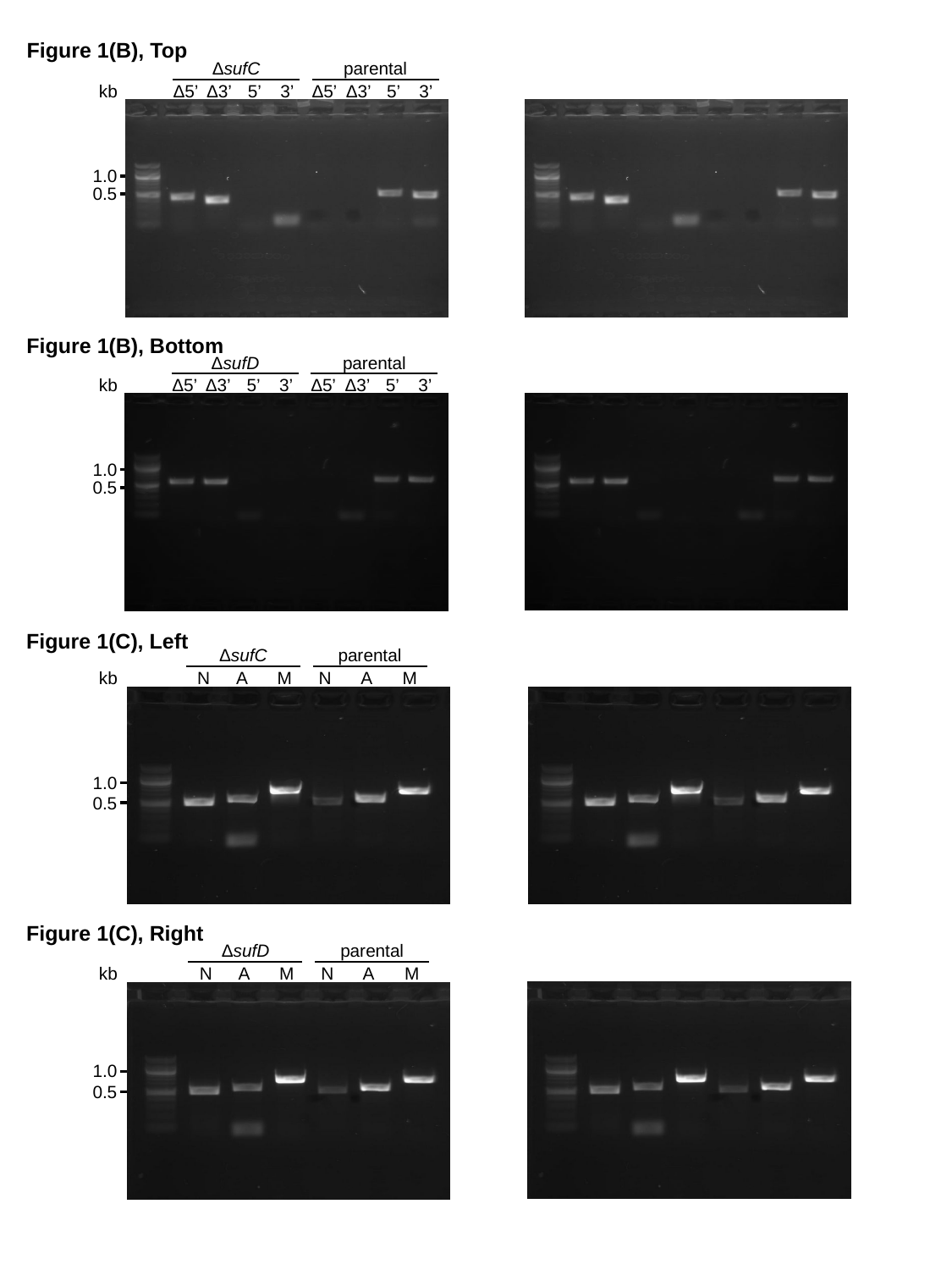

Figure 1(B), Top
ΔsufC
parental
kb
Δ5’
Δ3’
5’
3’
Δ5’
Δ3’
5’
3’
1.0
0.5
Figure 1(B), Bottom
ΔsufD
parental
kb
Δ5’
Δ3’
5’
3’
Δ5’
Δ3’
5’
3’
1.0
0.5
Figure 1(C), Left
ΔsufC
parental
kb
N
A
M
N
A
M
1.0
0.5
Figure 1(C), Right
ΔsufD
parental
kb
N
A
M
N
A
M
1.0
0.5

## Slide 2
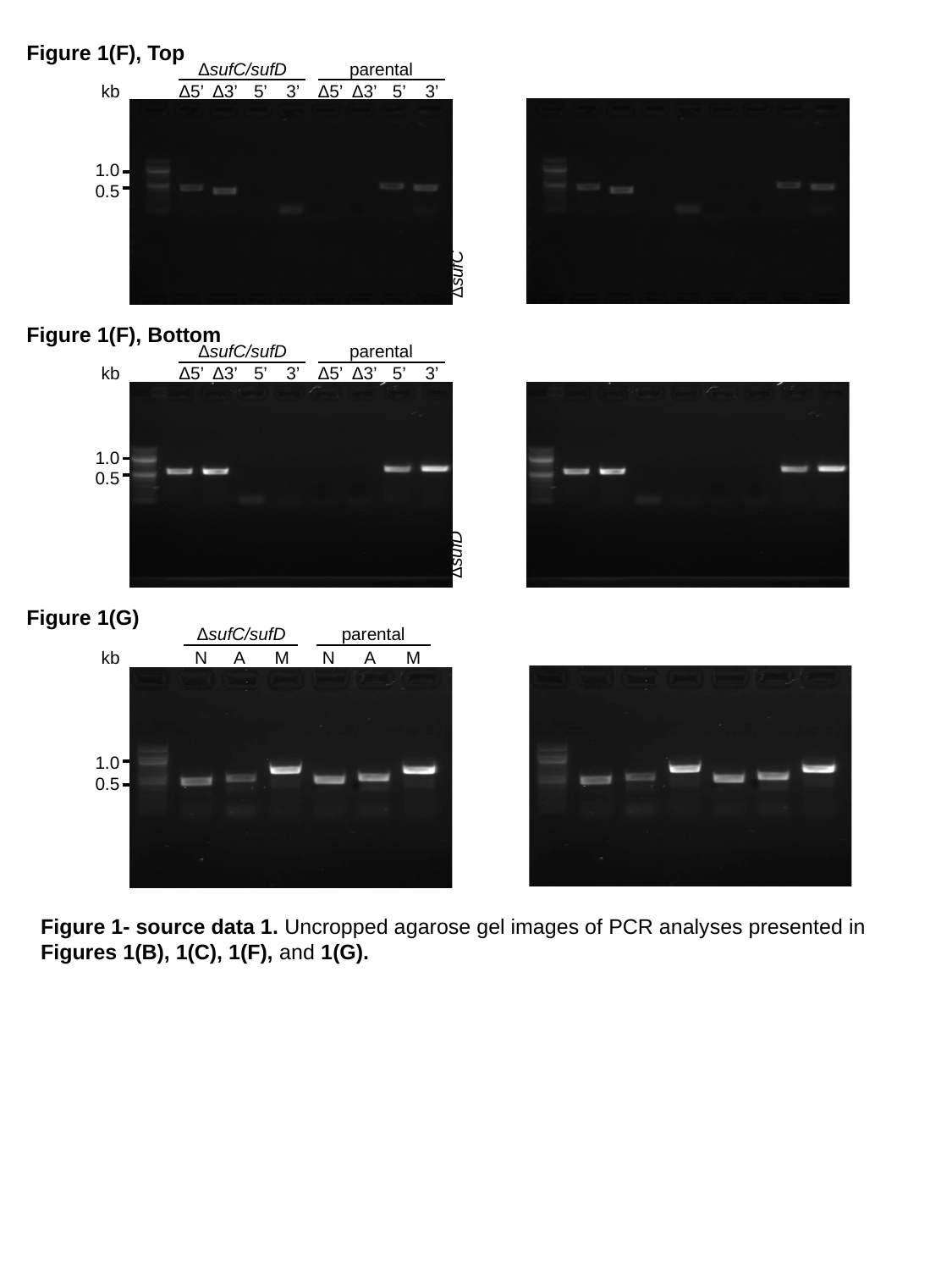

Figure 1(F), Top
ΔsufC/sufD
parental
kb
Δ5’
Δ3’
5’
3’
Δ5’
Δ3’
5’
3’
1.0
0.5
ΔsufC
Figure 1(F), Bottom
ΔsufC/sufD
parental
kb
Δ5’
Δ3’
5’
3’
Δ5’
Δ3’
5’
3’
1.0
0.5
ΔsufD
Figure 1(G)
ΔsufC/sufD
parental
kb
N
A
M
N
A
M
1.0
0.5
Figure 1- source data 1. Uncropped agarose gel images of PCR analyses presented in Figures 1(B), 1(C), 1(F), and 1(G).
