# Supplementary material for: The Plasmodium falciparum apicoplast cysteine desulfurase provides sulfur for both iron-sulfur cluster assembly and tRNA modification
Source: eLife. 2023 May 11;12:e84491. doi: 10.7554/eLife.84491 (PMC10219651; doi:10.7554/eLife.84491)
Supplement: Figure 1—source data 1. [file elife-84491-fig1-data1.zip › Figure 1 figure supplement 1.pdf]

A

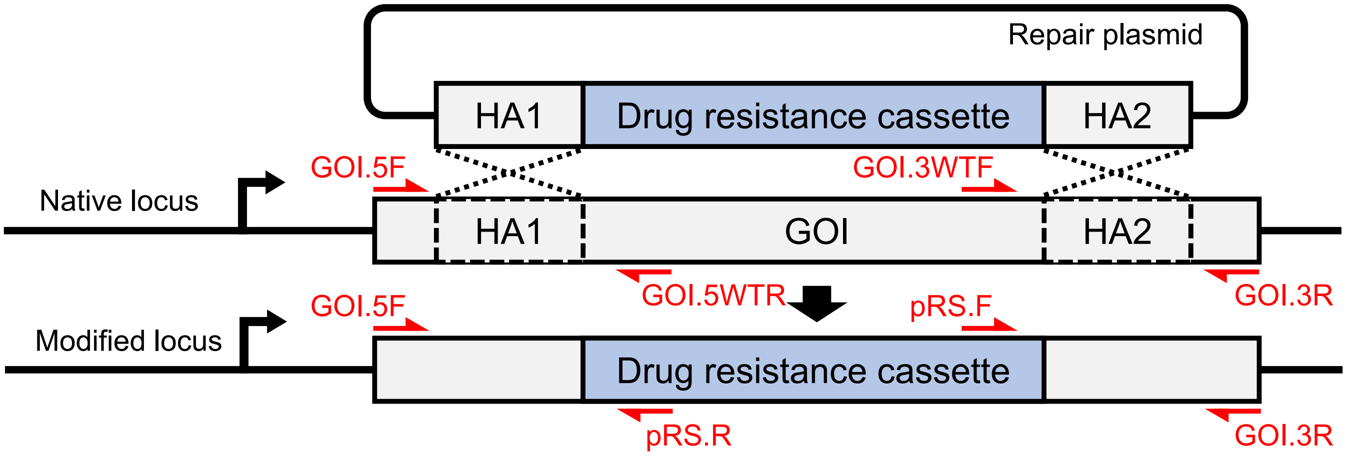

B

PCR reaction	Primer combination		Parasite line
	Forward	Reverse	
Δ5’	GOI.5F	pRS.R	Gene knockout lines
Δ3’	pRS.F	GOI.3R	
5’	GOI.5F	GOI.5WTR	
3’	GOI.3WTF	GOI.3R	
Δ5’	GOI.5F	pRS.R	PfMev (parental)
Δ3’	pRS.F	GOI.3R	
5’	GOI.5F	GOI.5WTR	
3’	GOI.3WTF	GOI.3R	

C

	Anticipated amplicon sizes (bp)			
Gene of interest	Δ5’	Δ3’	5’	3’
sufC	467	389	486	457
sufD	593	582	636	649
sufE	490	543	556	656
sufS	584	677	691	754
mnmA	605	577	626	617
