# Supplementary figures and images for: The Plasmodium falciparum apicoplast cysteine desulfurase provides sulfur for both iron-sulfur cluster assembly and tRNA modification
Source: eLife. 2023 May 11;12:e84491. doi: 10.7554/eLife.84491 (PMC10219651; doi:10.7554/eLife.84491)

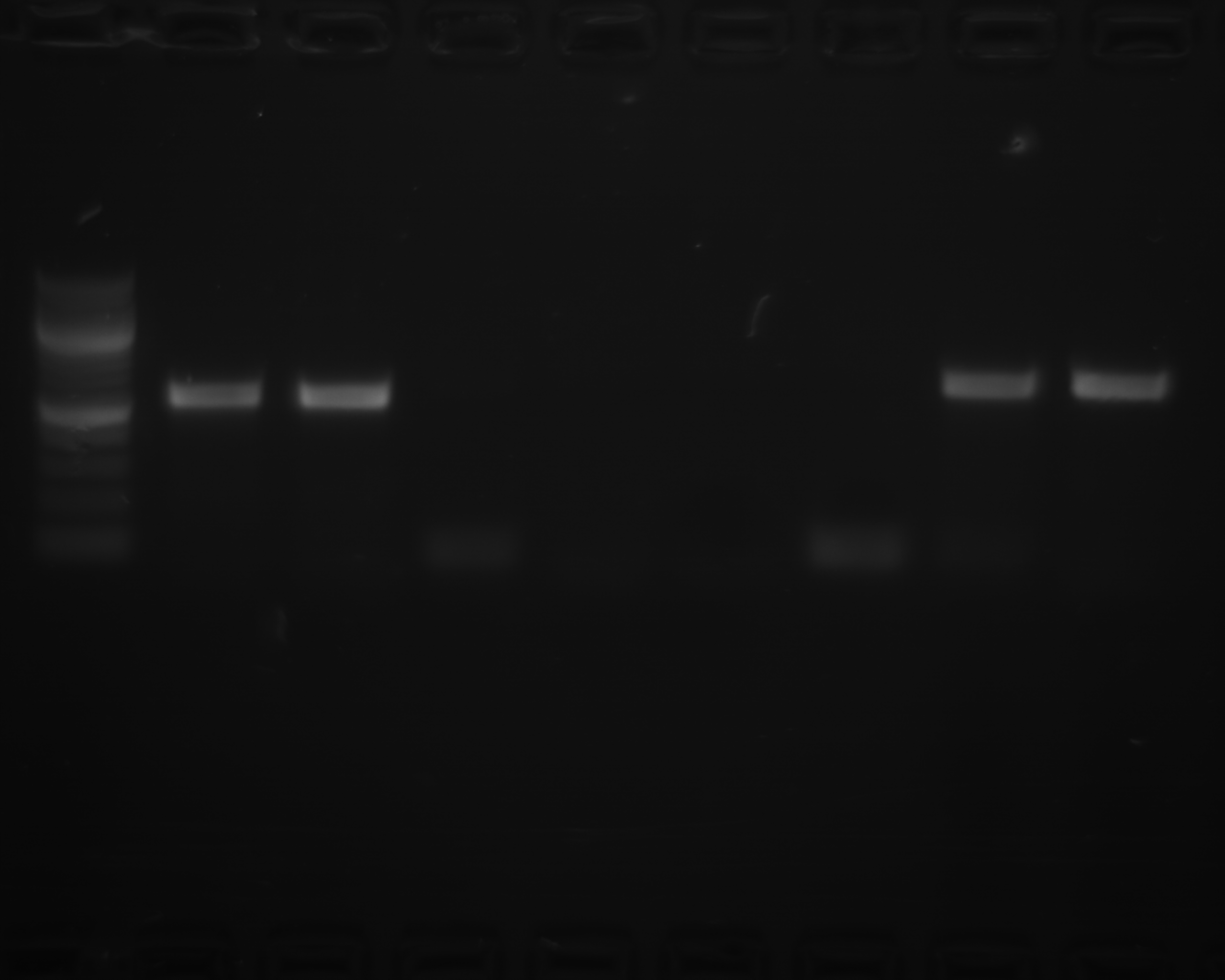

Supplement: Figure 1—source data 1. [file elife-84491-fig1-data1.zip › Figure 1- source data 1/Figure-1(B)_bottom.tif]

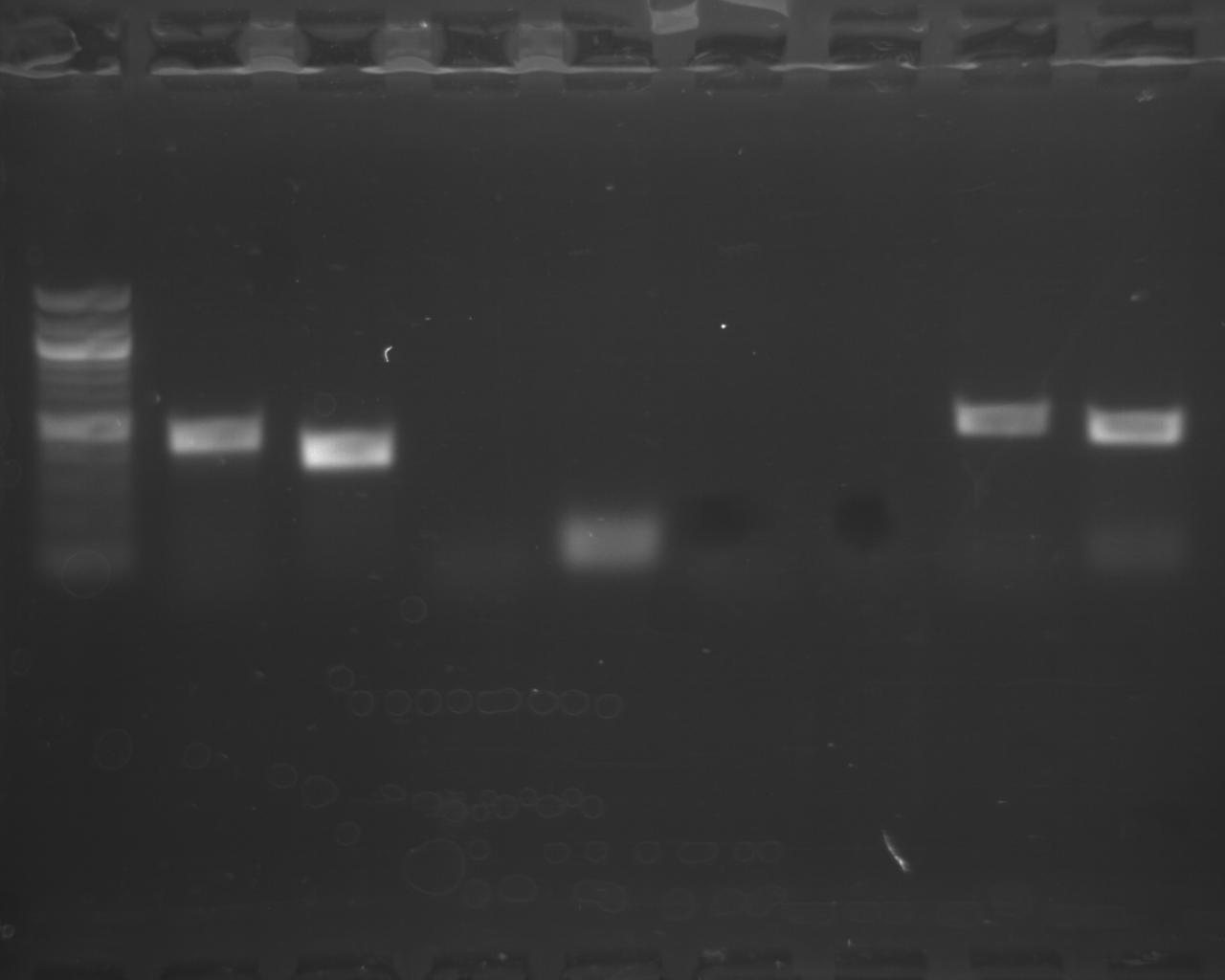

Supplement: Figure 1—source data 1. [file elife-84491-fig1-data1.zip › Figure 1- source data 1/Figure-1(B)_top.jpg]

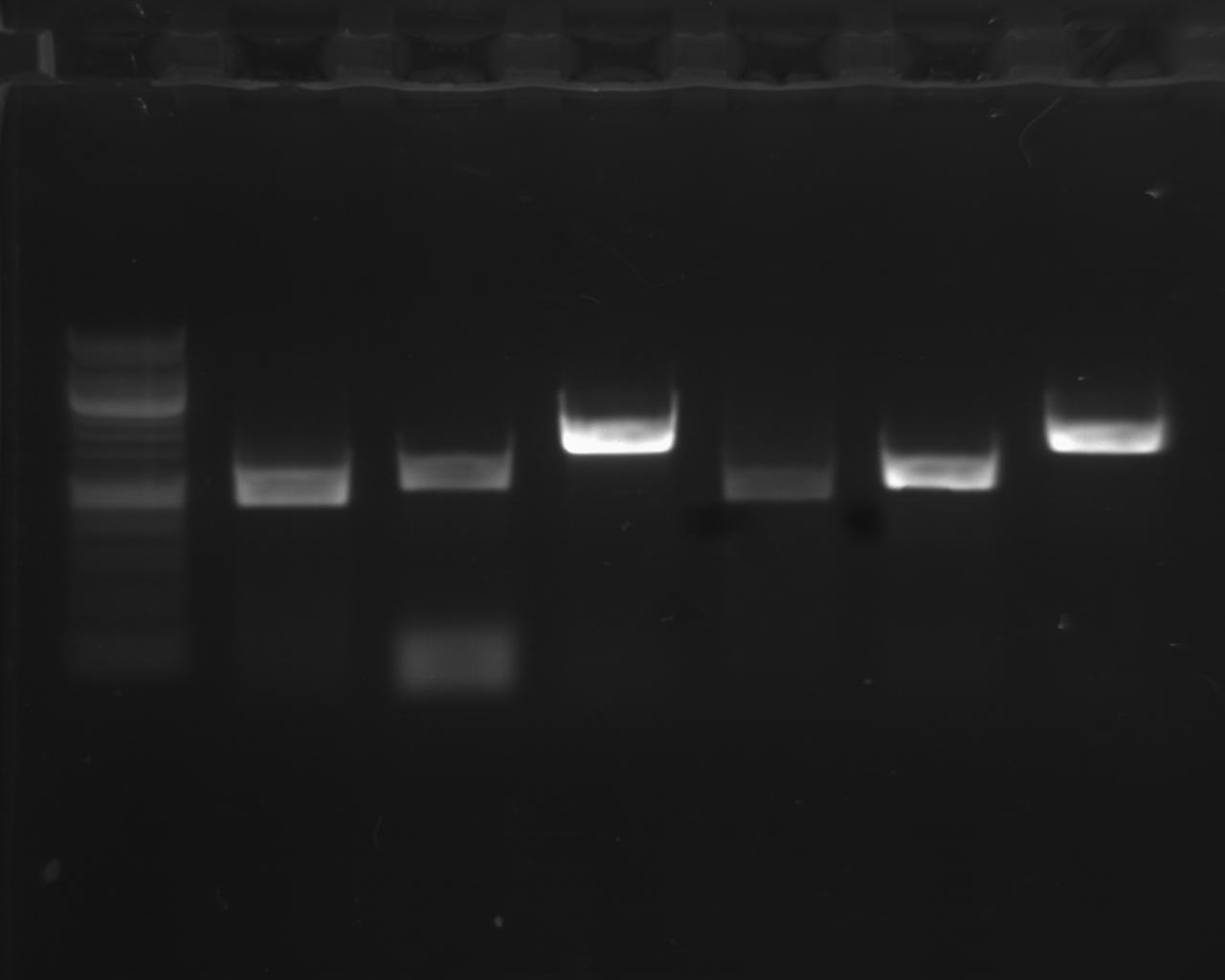

Supplement: Figure 1—source data 1. [file elife-84491-fig1-data1.zip › Figure 1- source data 1/Figure-1(C)_right.jpg]

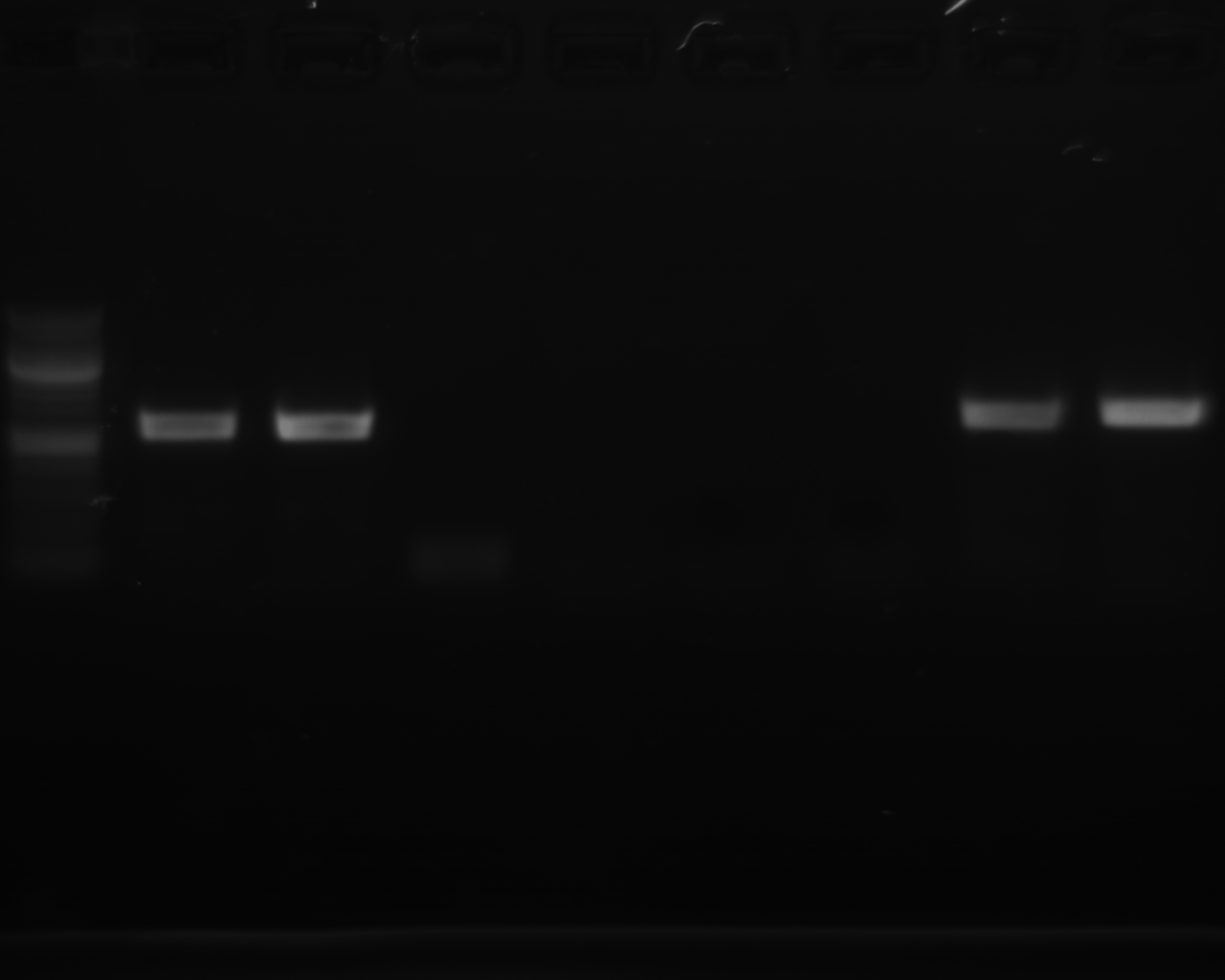

Supplement: Figure 1—source data 1. [file elife-84491-fig1-data1.zip › Figure 1- source data 1/Figure-1(F)_bottom.tif]

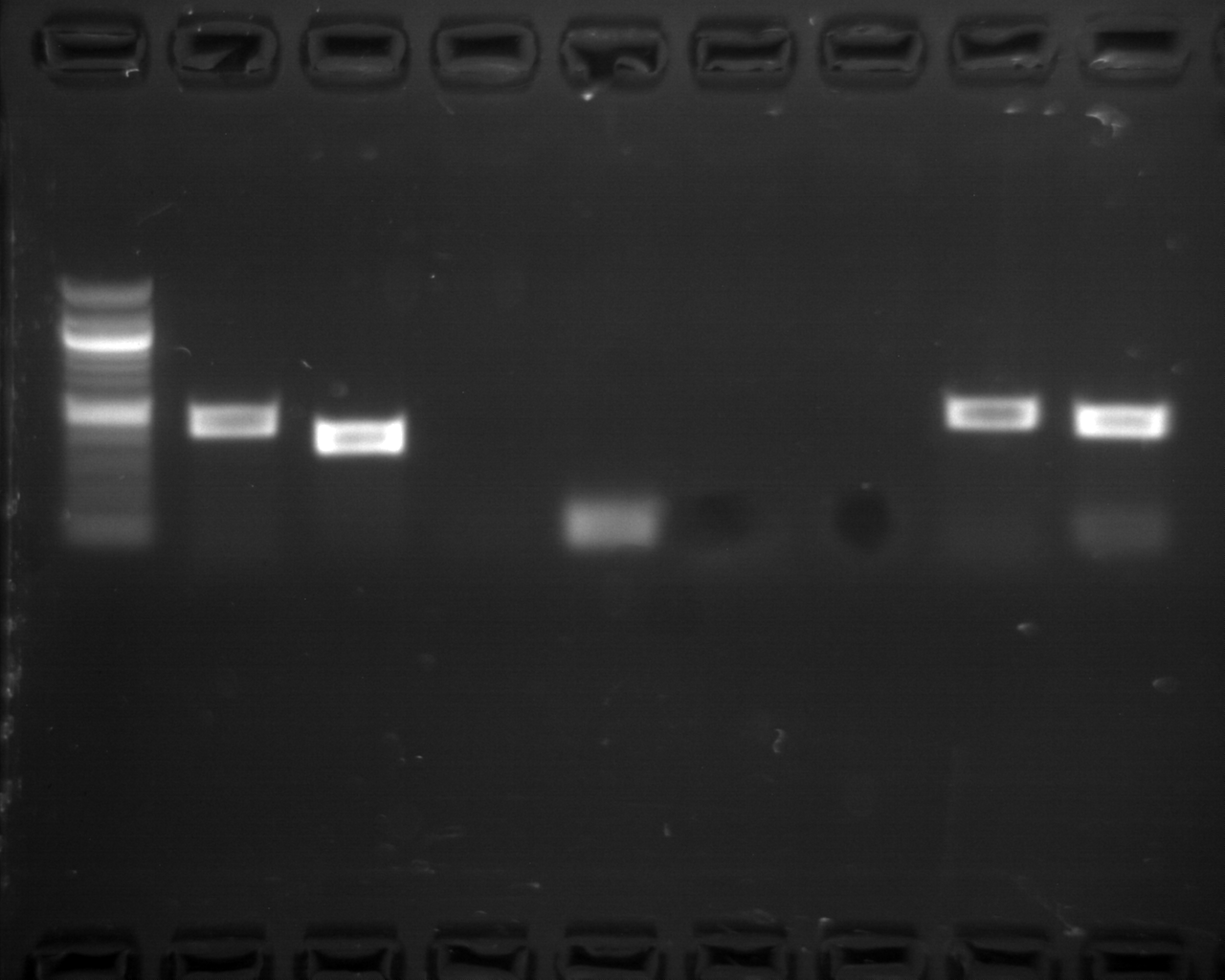

Supplement: Figure 1—source data 1. [file elife-84491-fig1-data1.zip › Figure 1- source data 1/Figure-1(F)_top.tif]

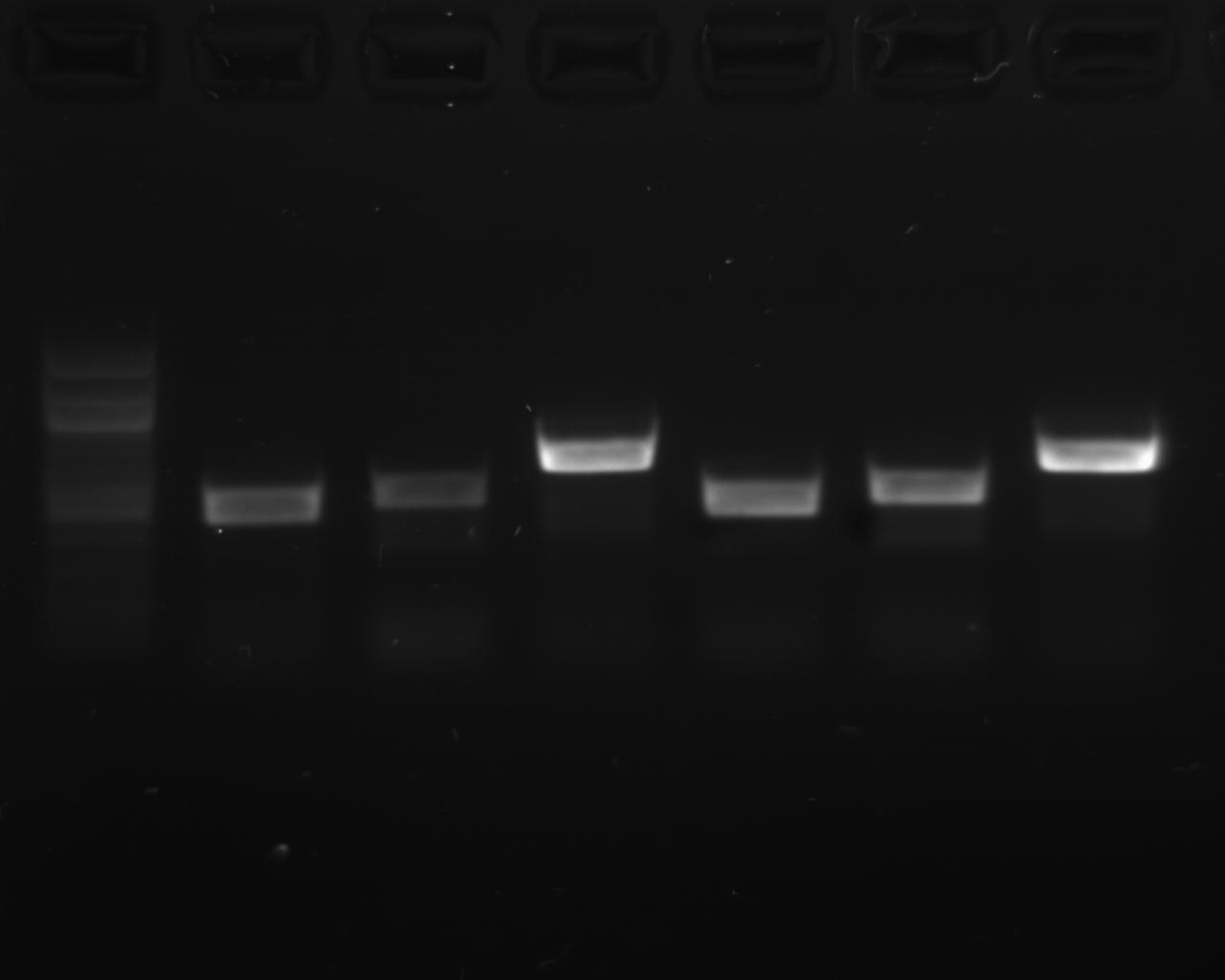

Supplement: Figure 1—source data 1. [file elife-84491-fig1-data1.zip › Figure 1- source data 1/Figure-1(G).jpg]

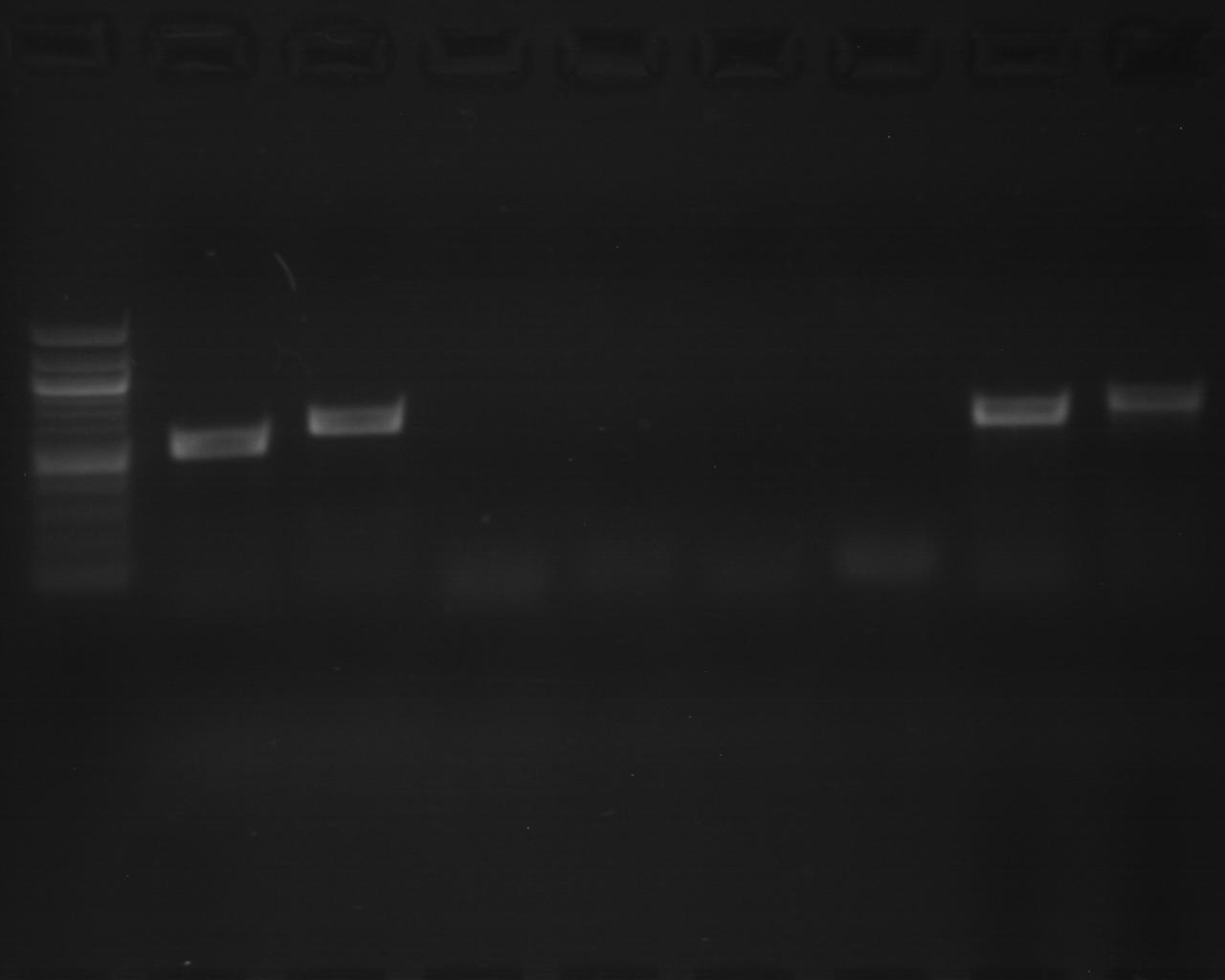

Supplement: Figure 2—source data 1. [file elife-84491-fig2-data1.zip › Figure 2- source data 1/Figure-2(A)_bottom.jpg]

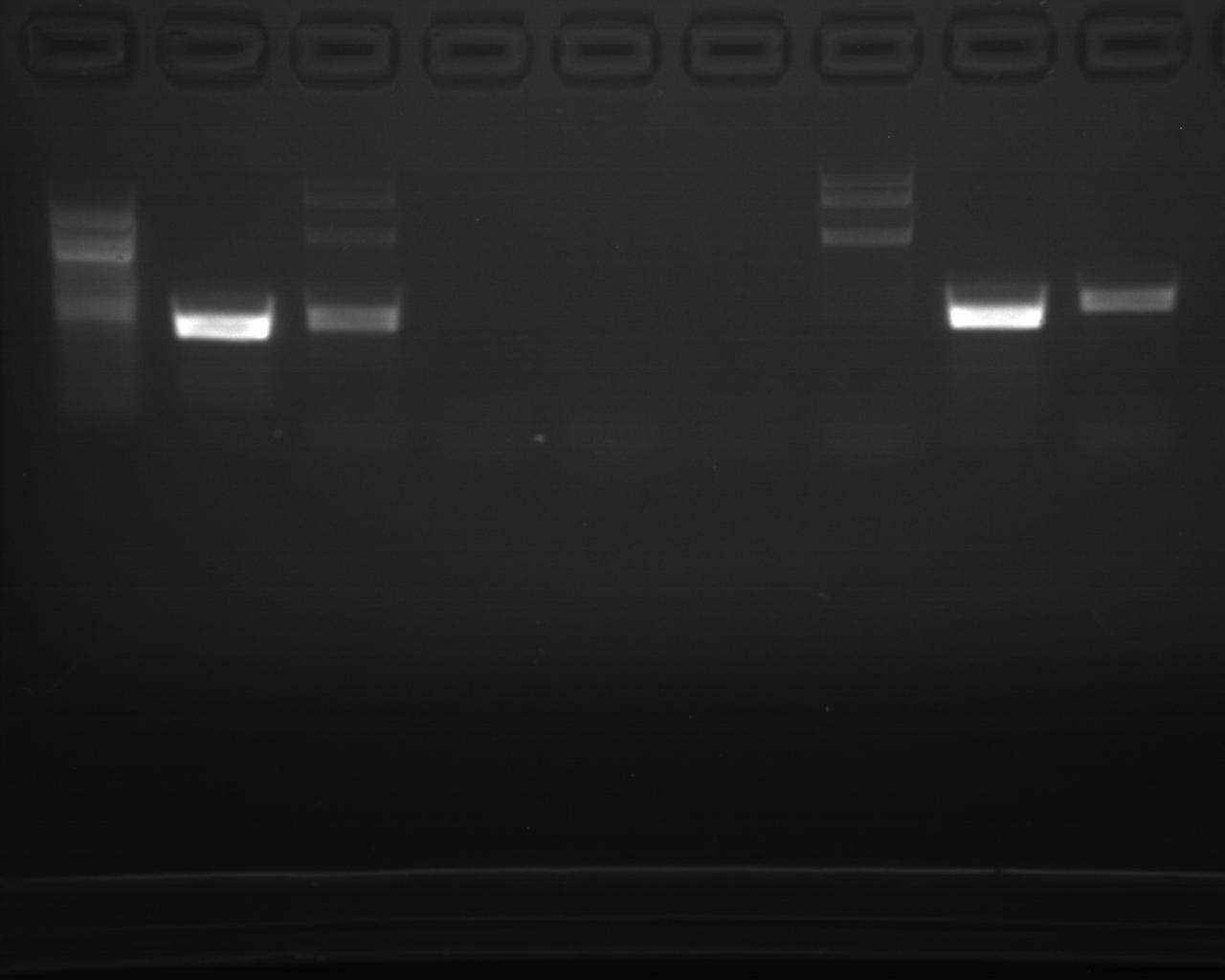

Supplement: Figure 2—source data 1. [file elife-84491-fig2-data1.zip › Figure 2- source data 1/Figure-2(A)_top.jpg]

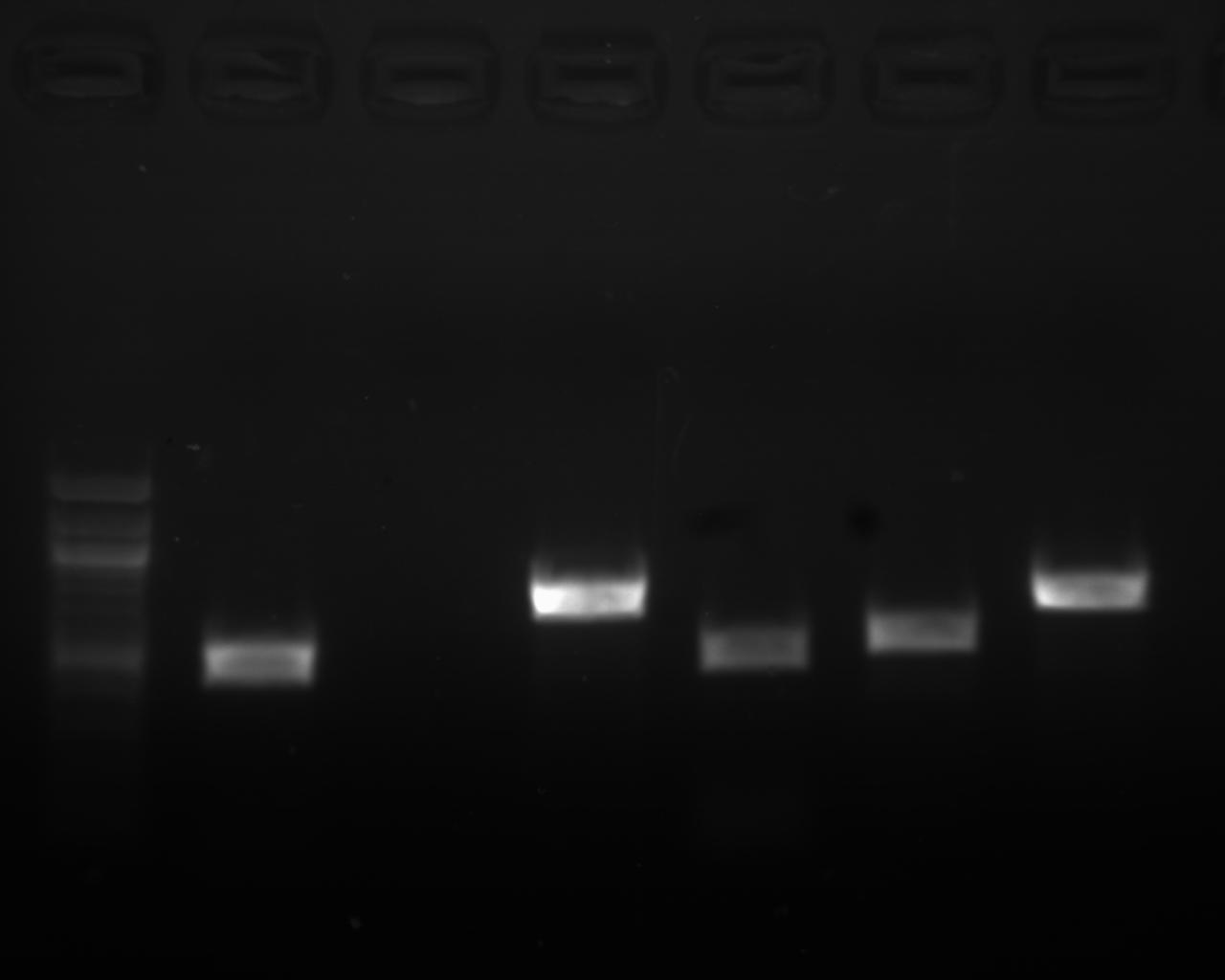

Supplement: Figure 2—source data 1. [file elife-84491-fig2-data1.zip › Figure 2- source data 1/Figure-2(B)_bottom.jpg]

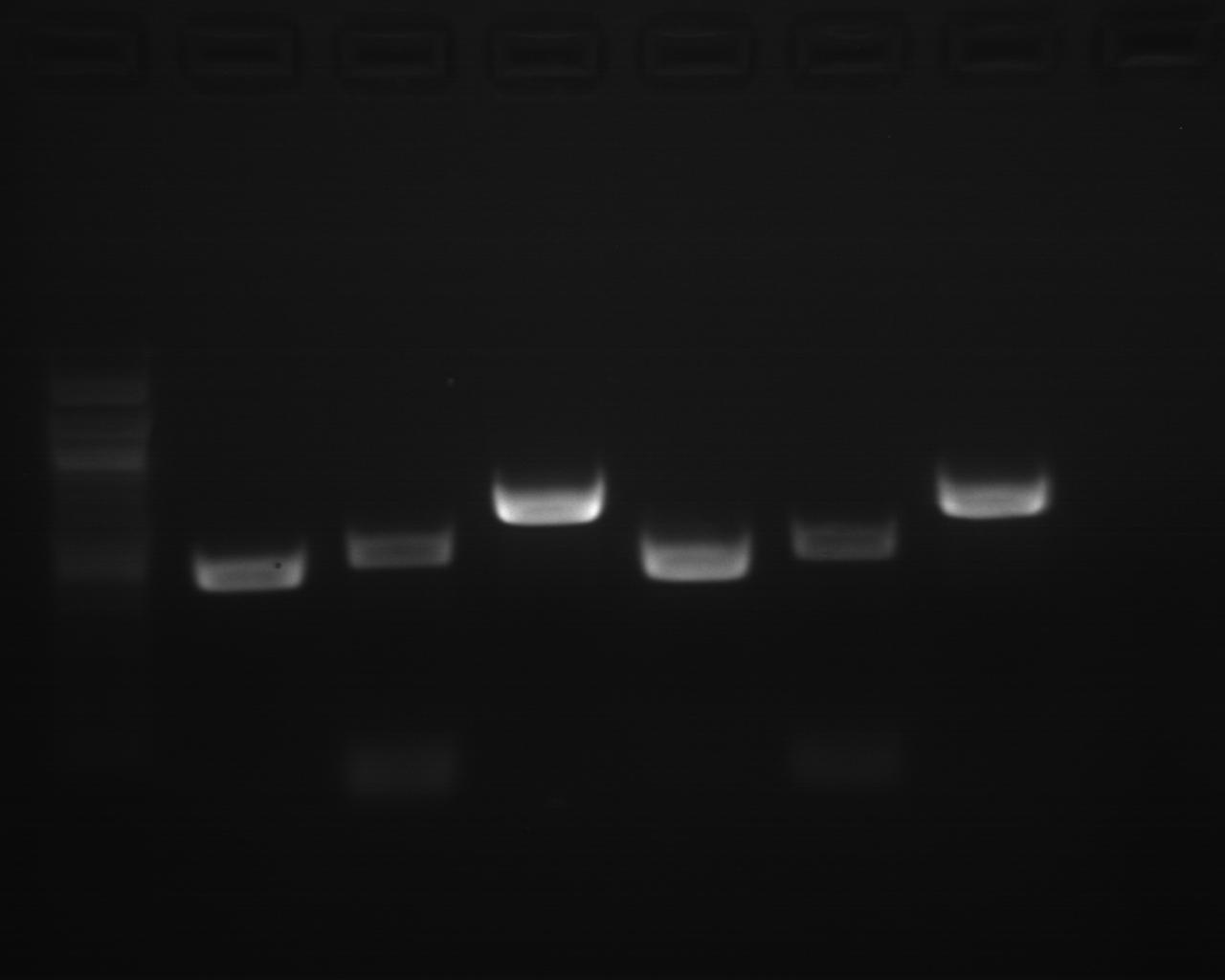

Supplement: Figure 2—source data 1. [file elife-84491-fig2-data1.zip › Figure 2- source data 1/Figure-2(B)_top.jpg]

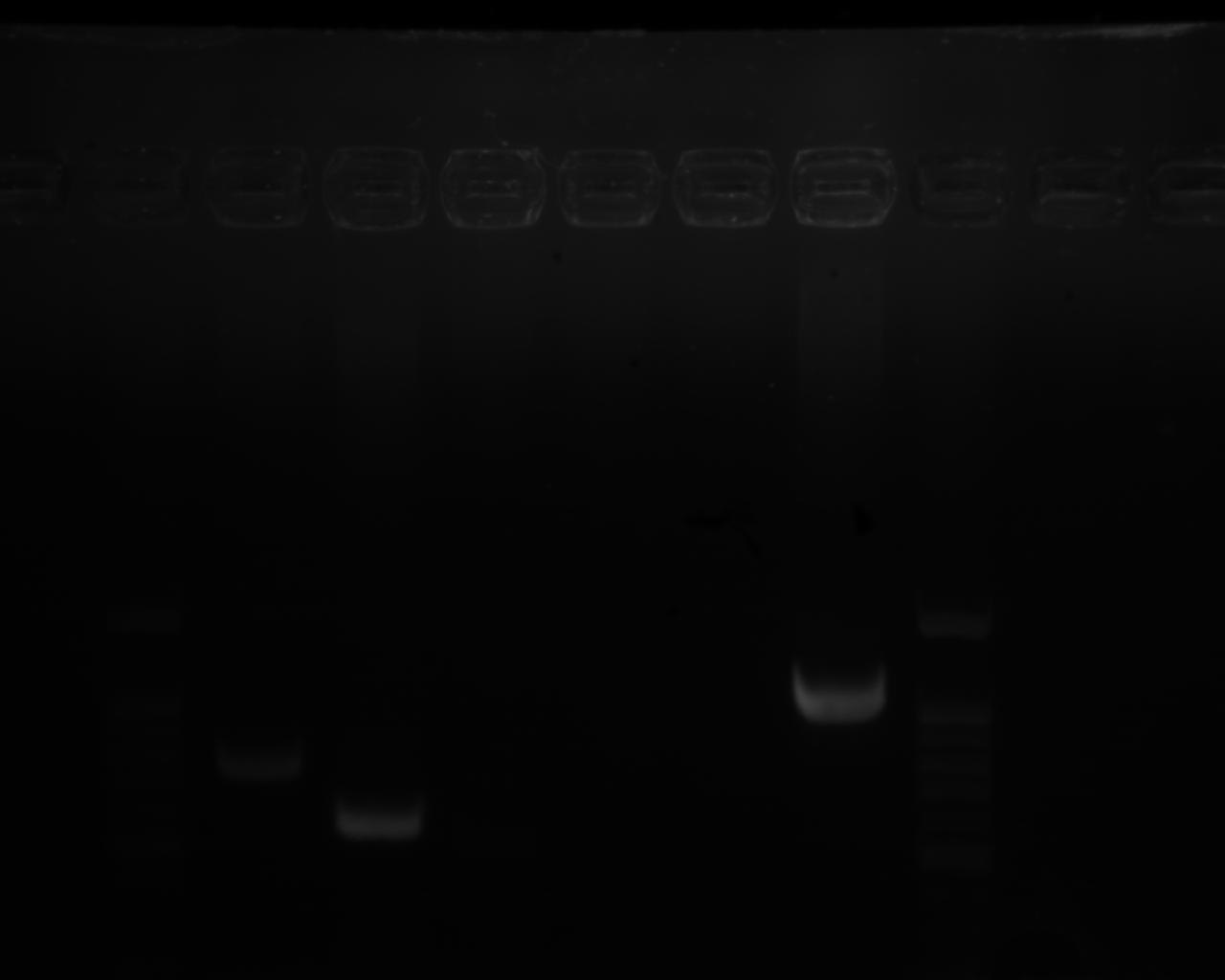

Supplement: Figure 3—source data 1. [file elife-84491-fig3-data1.zip › Figure 3 - source data 1/Figure-3(C).jpg]

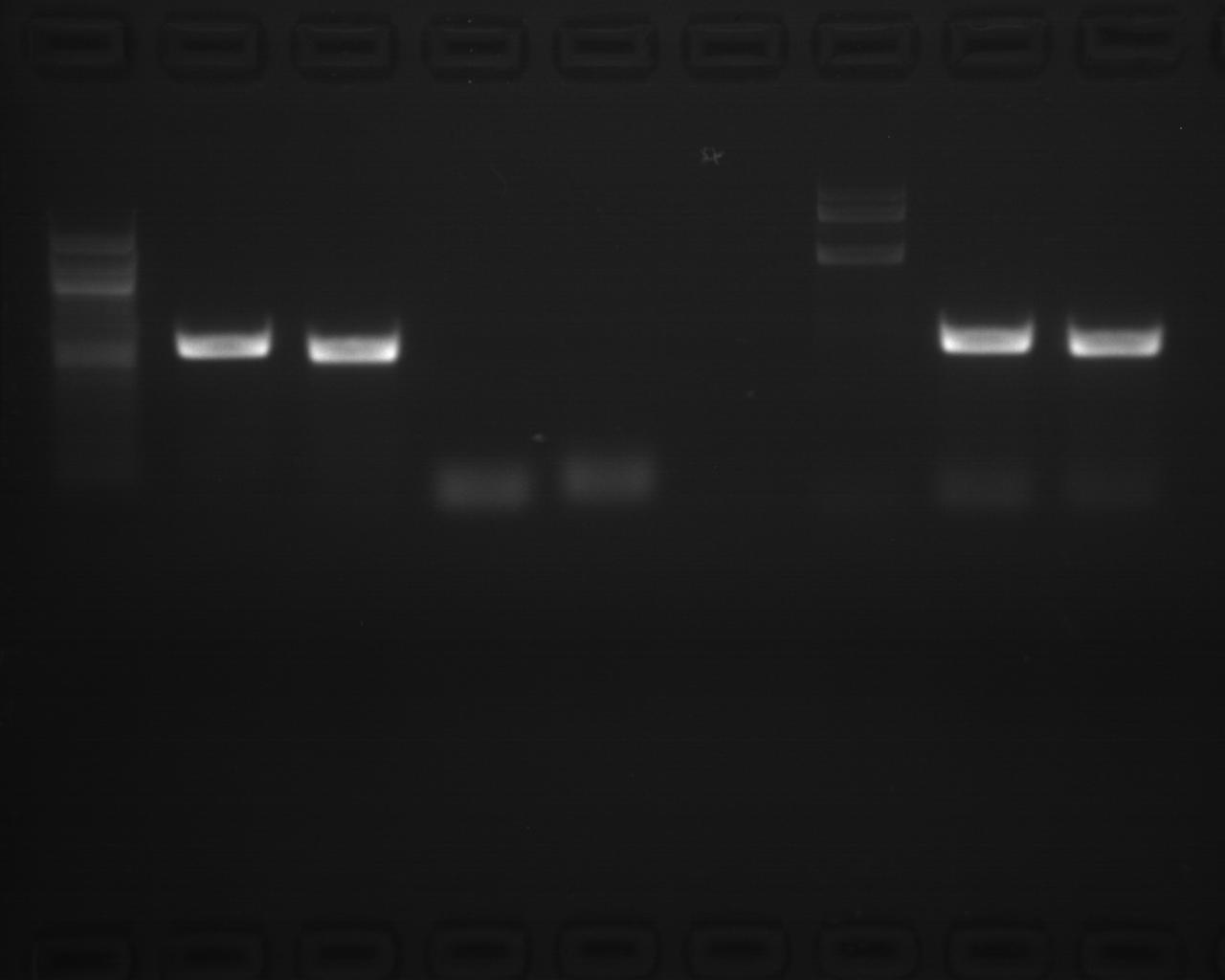

Supplement: Figure 3—source data 1. [file elife-84491-fig3-data1.zip › Figure 3 - source data 1/Figure-3(I).jpg]

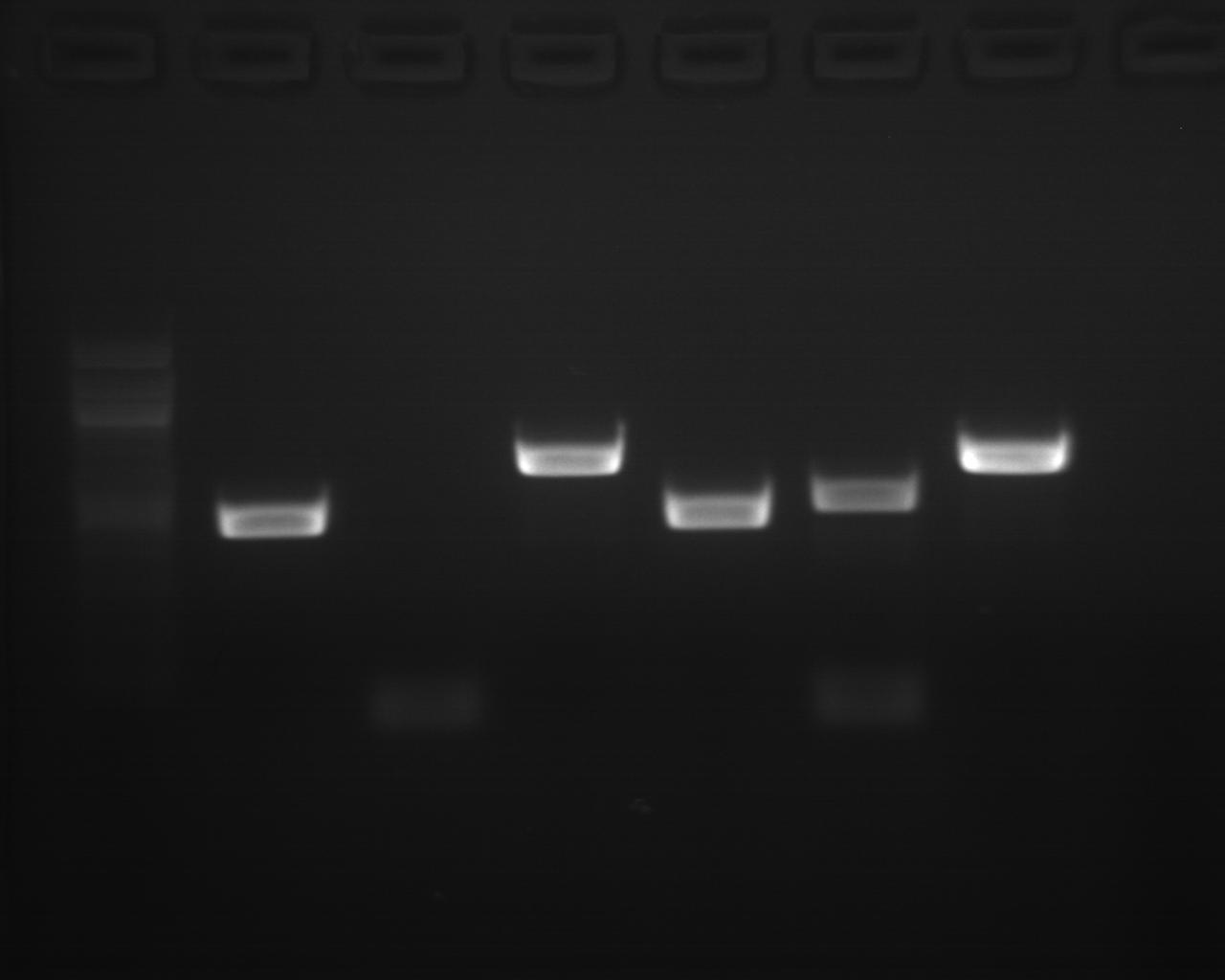

Supplement: Figure 3—source data 1. [file elife-84491-fig3-data1.zip › Figure 3 - source data 1/Figure-3(J).jpg]

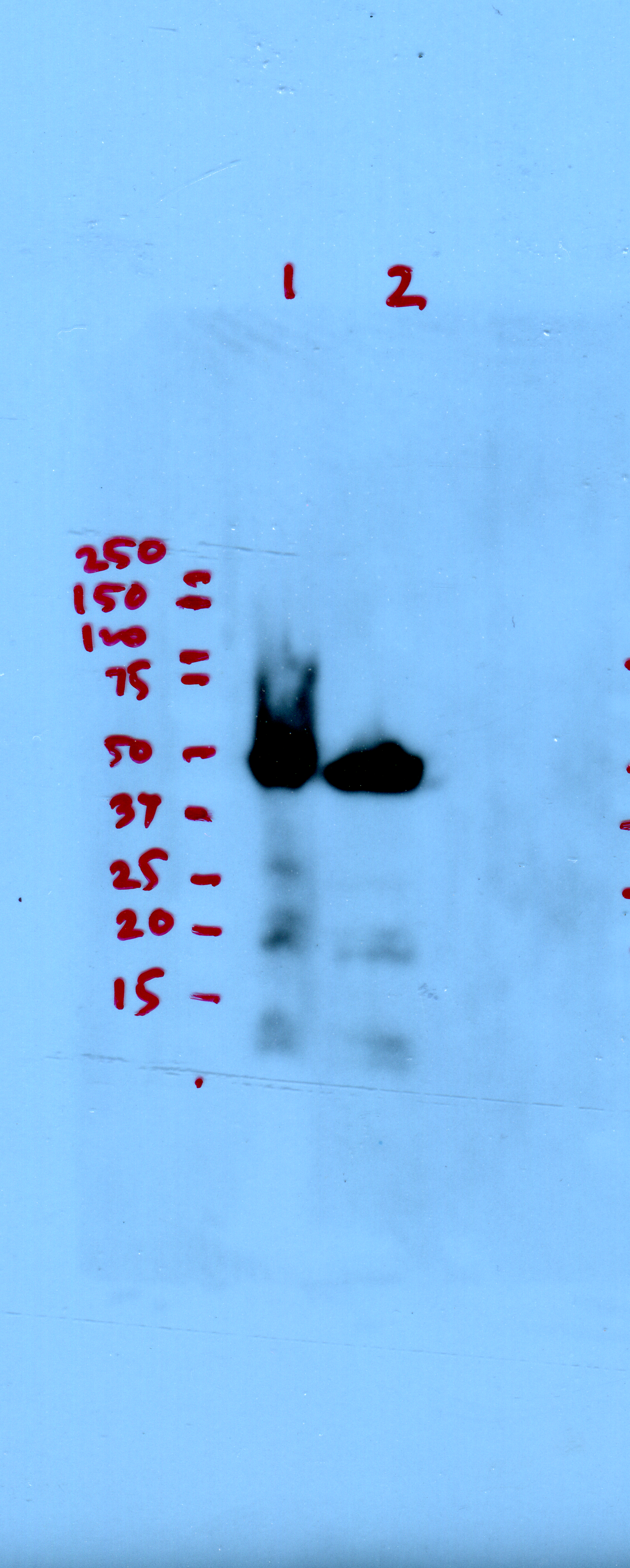

Supplement: Figure 3—figure supplement 4—source data 2. — The red boxes correspond to the cropped images in Figure 3—figure supplement 4C. [file elife-84491-fig3-figsupp4-data2.zip › Figure 3- figure supplement 4- source data 1/Figure 3- figure supplement4_bottom.tif]

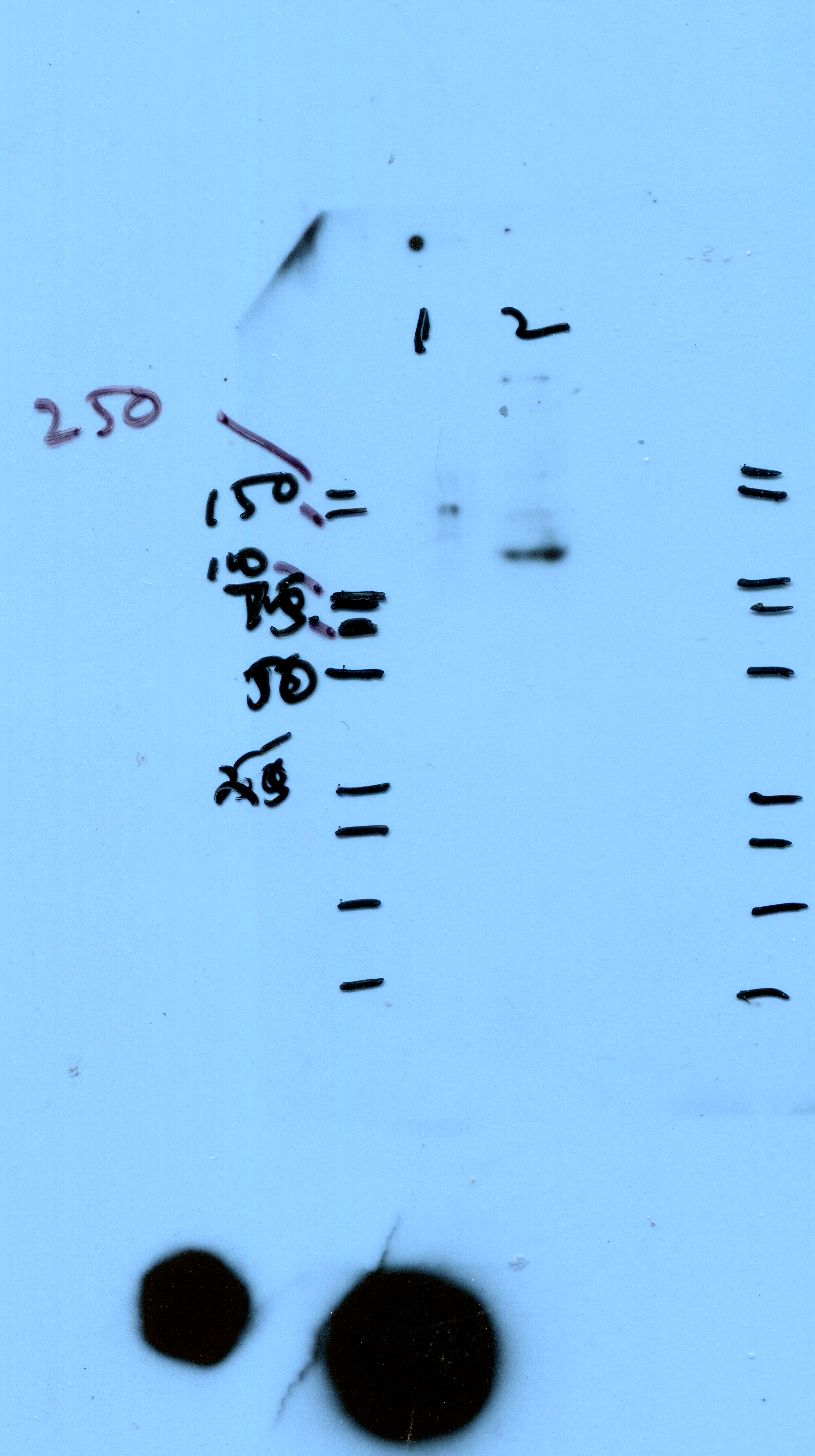

Supplement: Figure 3—figure supplement 4—source data 2. — The red boxes correspond to the cropped images in Figure 3—figure supplement 4C. [file elife-84491-fig3-figsupp4-data2.zip › Figure 3- figure supplement 4- source data 1/Figure 3- figure supplement4_top.tif]

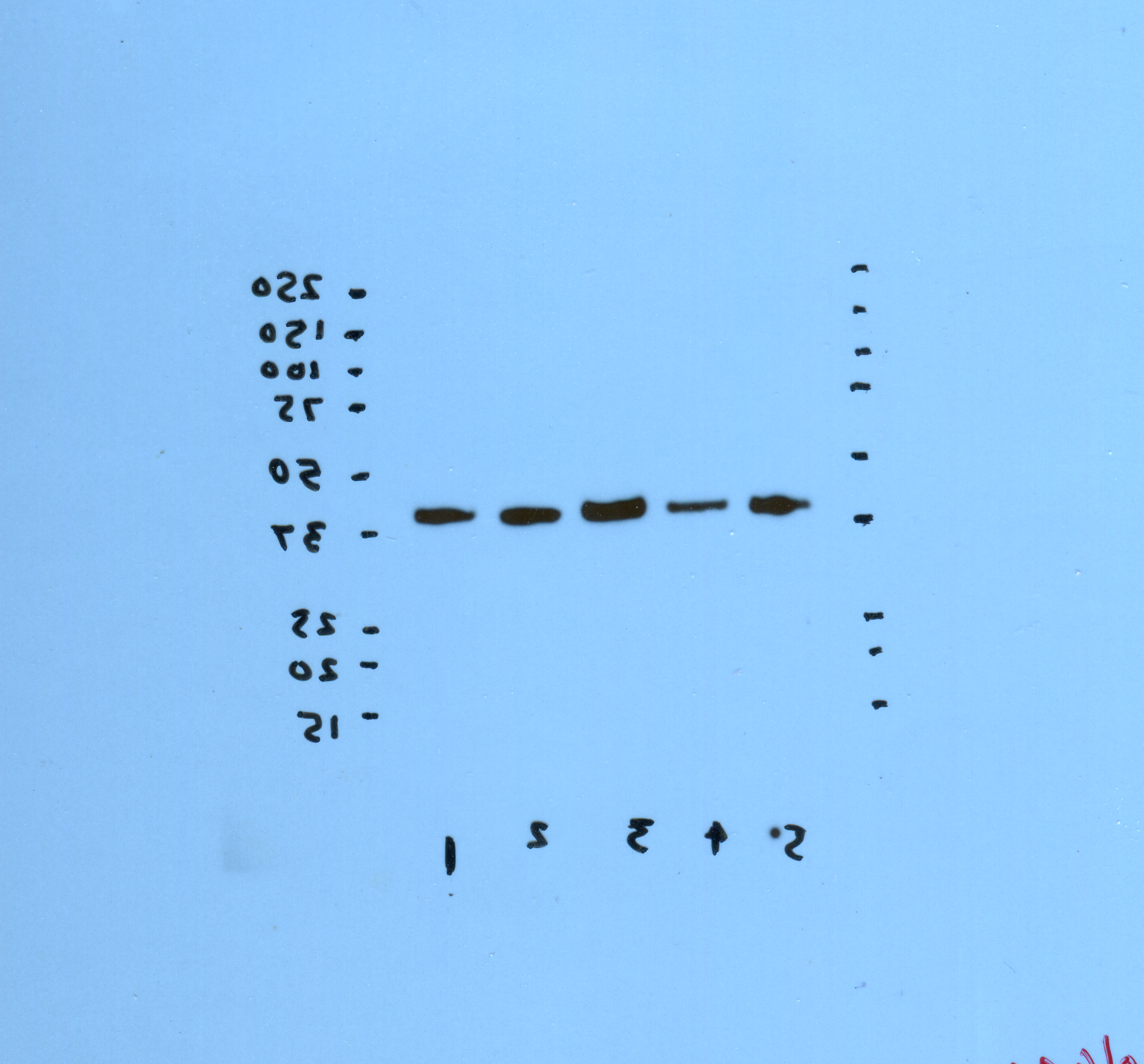

Supplement: Figure 3—figure supplement 5—source data 1. — The red boxes correspond to the cropped images in Figure 3—figure supplement 5. Blots were inverted for correct orientation. [file elife-84491-fig3-figsupp5-data1.zip › Figure 3- figure supplement 5- source data 1/Figure 3- figure supplement5_bottom.tif]

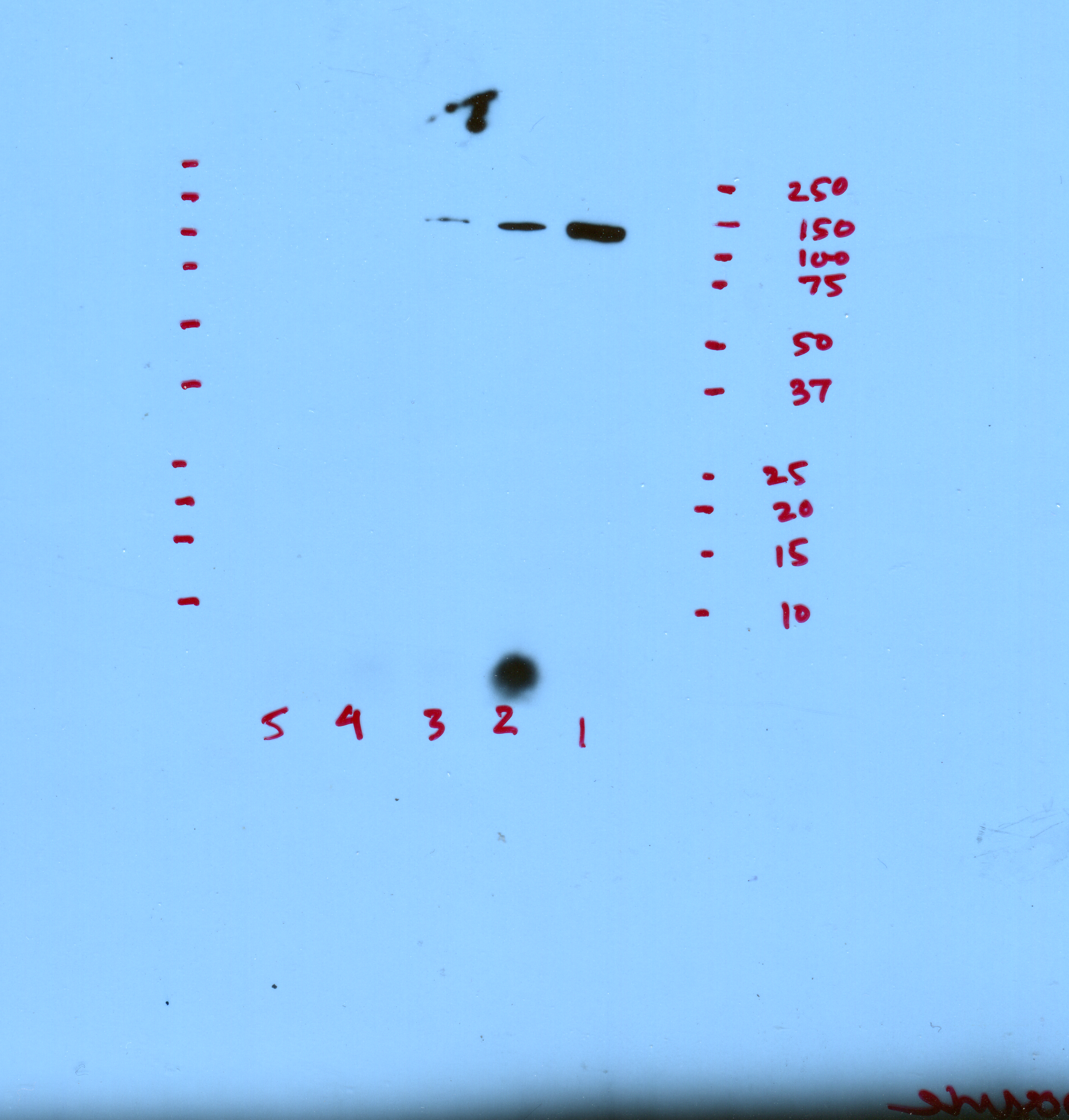

Supplement: Figure 3—figure supplement 5—source data 1. — The red boxes correspond to the cropped images in Figure 3—figure supplement 5. Blots were inverted for correct orientation. [file elife-84491-fig3-figsupp5-data1.zip › Figure 3- figure supplement 5- source data 1/Figure 3- figure supplement5_top.tif]

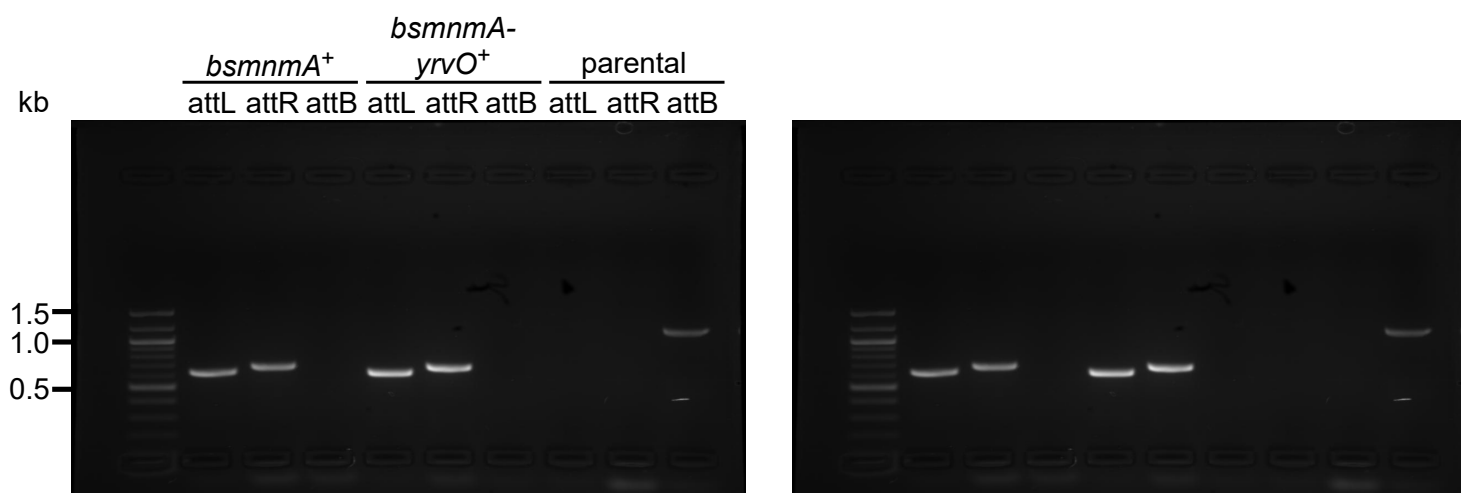

**Figure 4- source data 1.** Uncropped agarose gel images of PCR analyses presented in Figure 4(B).

Supplement: Figure 4—source data 1. [file elife-84491-fig4-data1.zip › Figure 4- source data 1/Figure 4- source data 1.pdf]

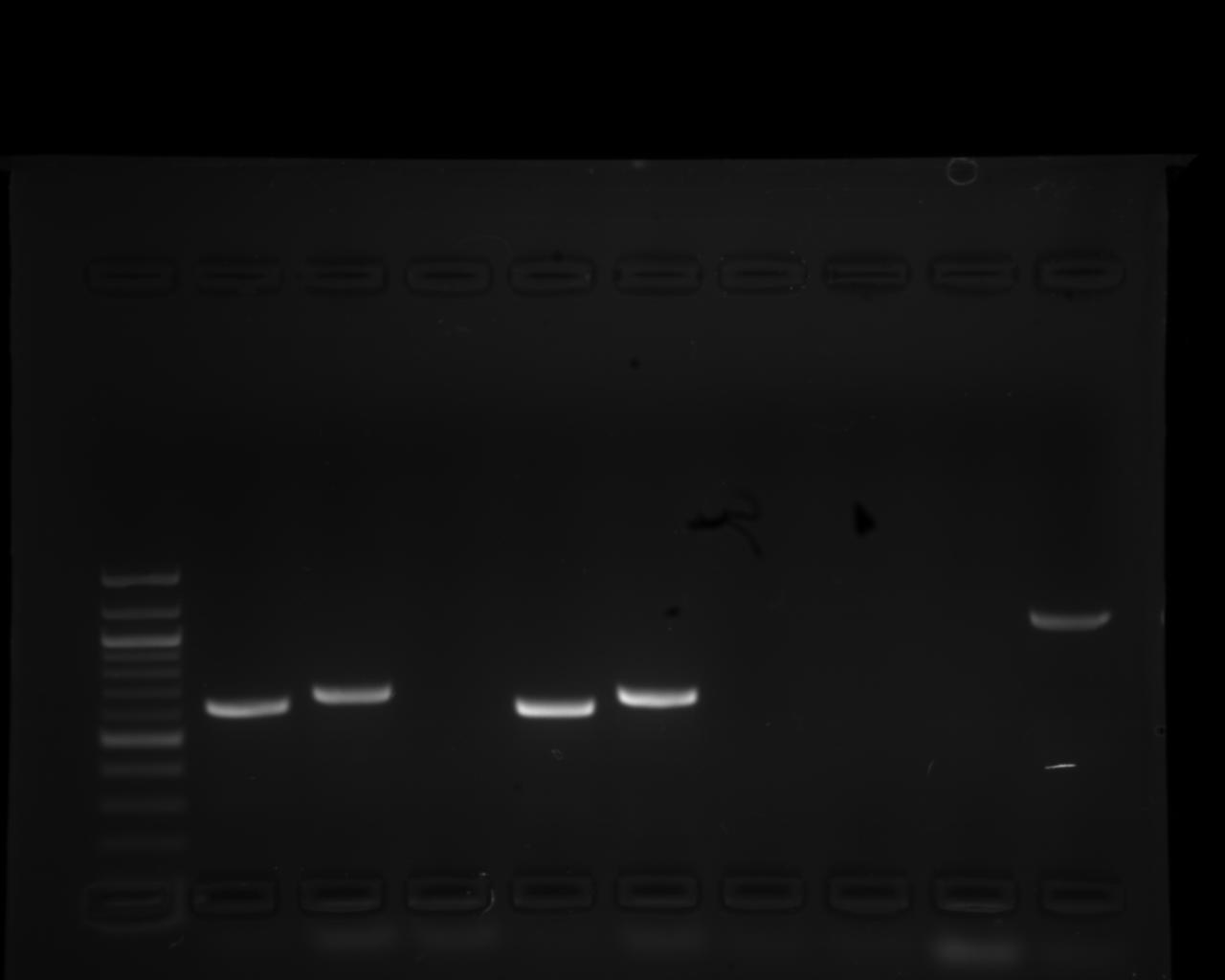

Supplement: Figure 4—source data 1. [file elife-84491-fig4-data1.zip › Figure 4- source data 1/Figure-4(B).jpg]

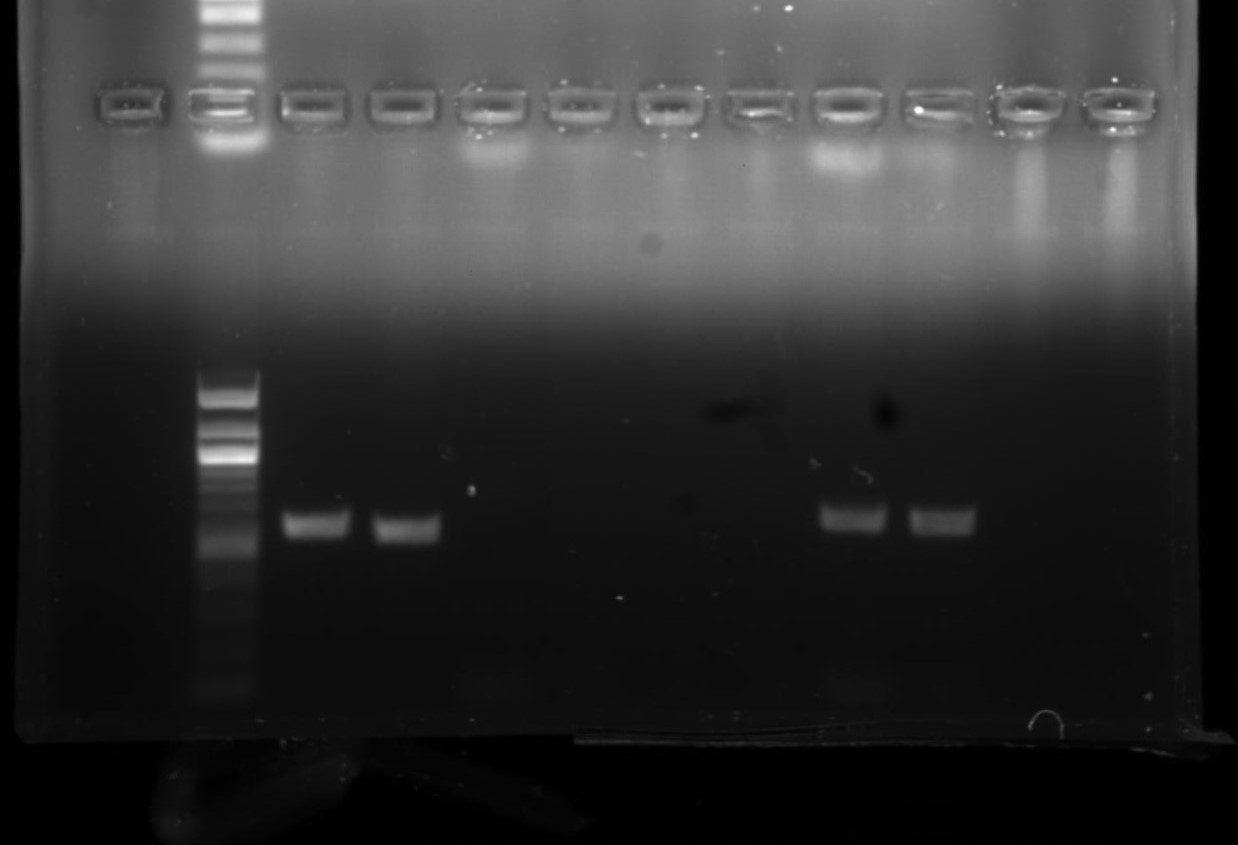

Supplement: Figure 5—source data 1. [file elife-84491-fig5-data1.zip › Figure 5- source data 1/Figure-5(A).jpg]

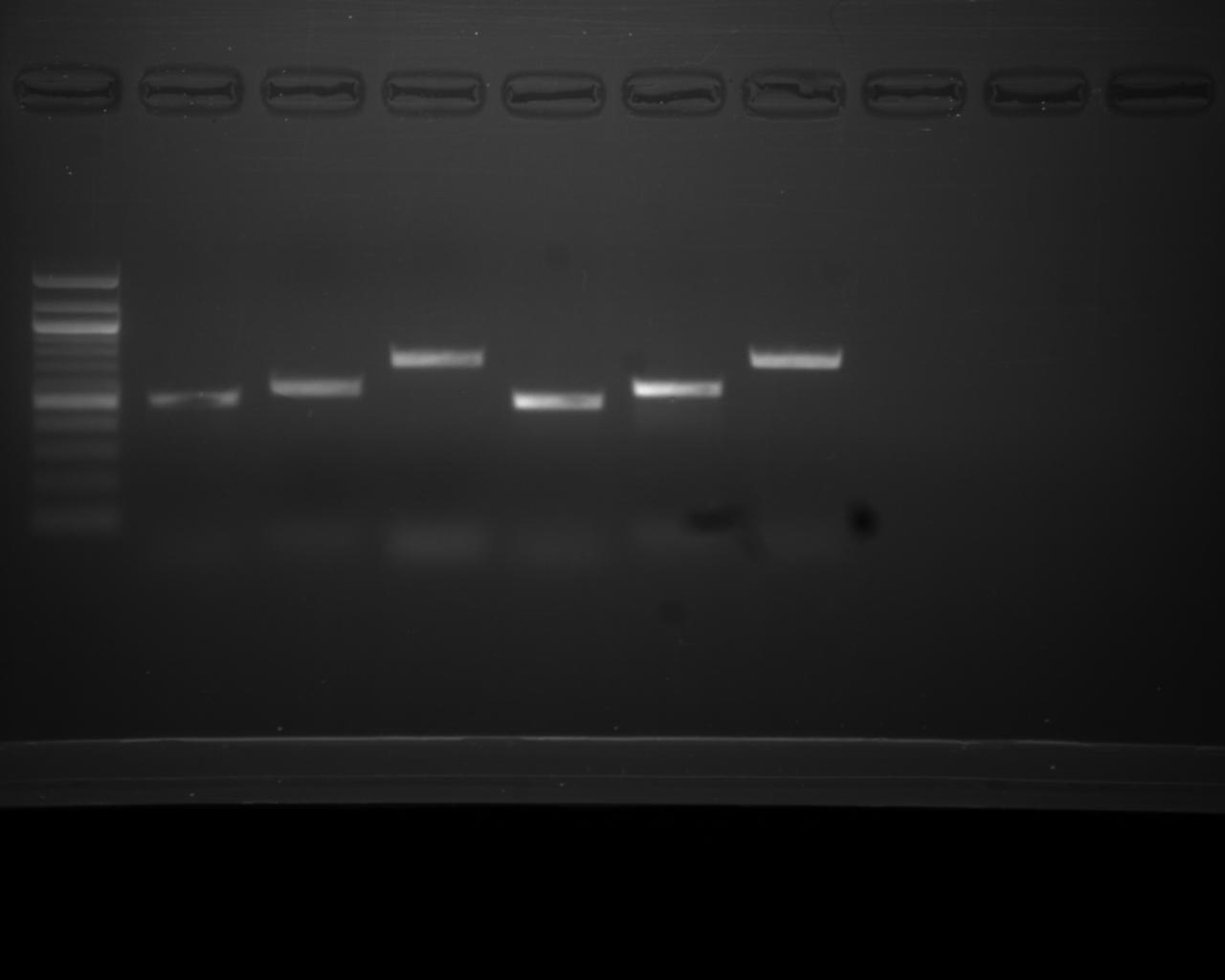

Supplement: Figure 5—source data 1. [file elife-84491-fig5-data1.zip › Figure 5- source data 1/Figure-5(C).jpg]

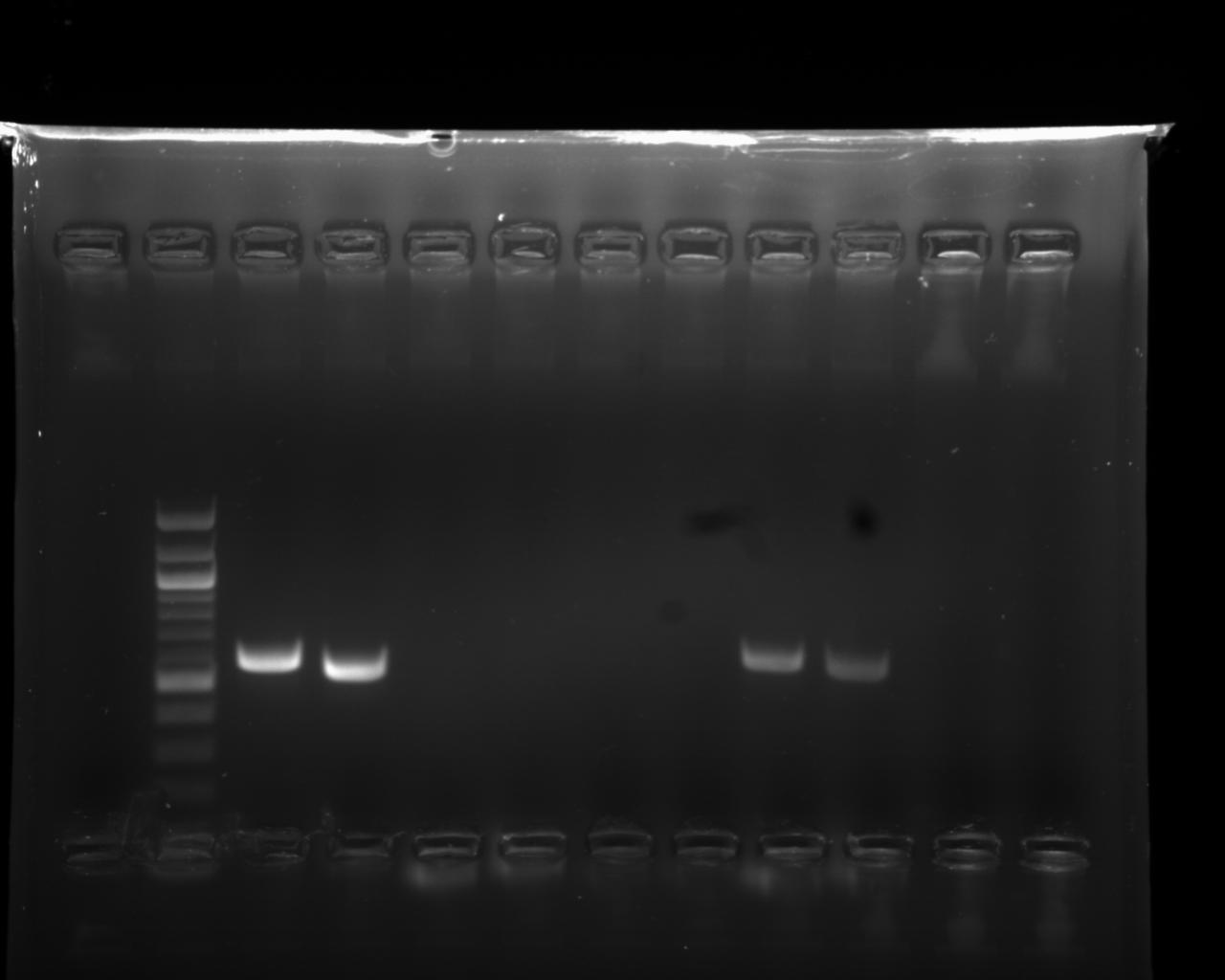

Supplement: Figure 6—source data 1. [file elife-84491-fig6-data1.zip › Figure 6- source data 1/Figure-6(A).jpg]

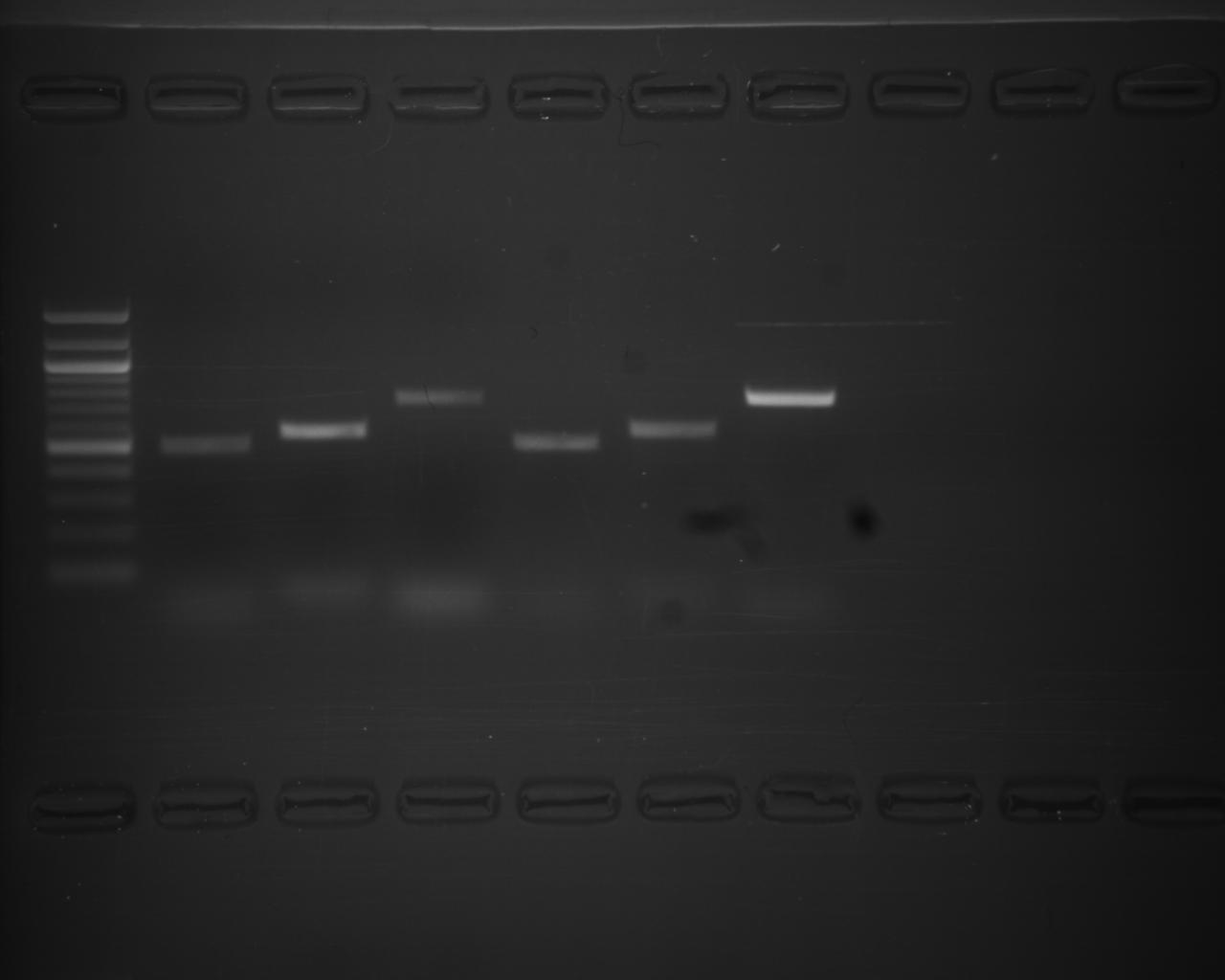

Supplement: Figure 6—source data 1. [file elife-84491-fig6-data1.zip › Figure 6- source data 1/Figure-6(C).jpg]

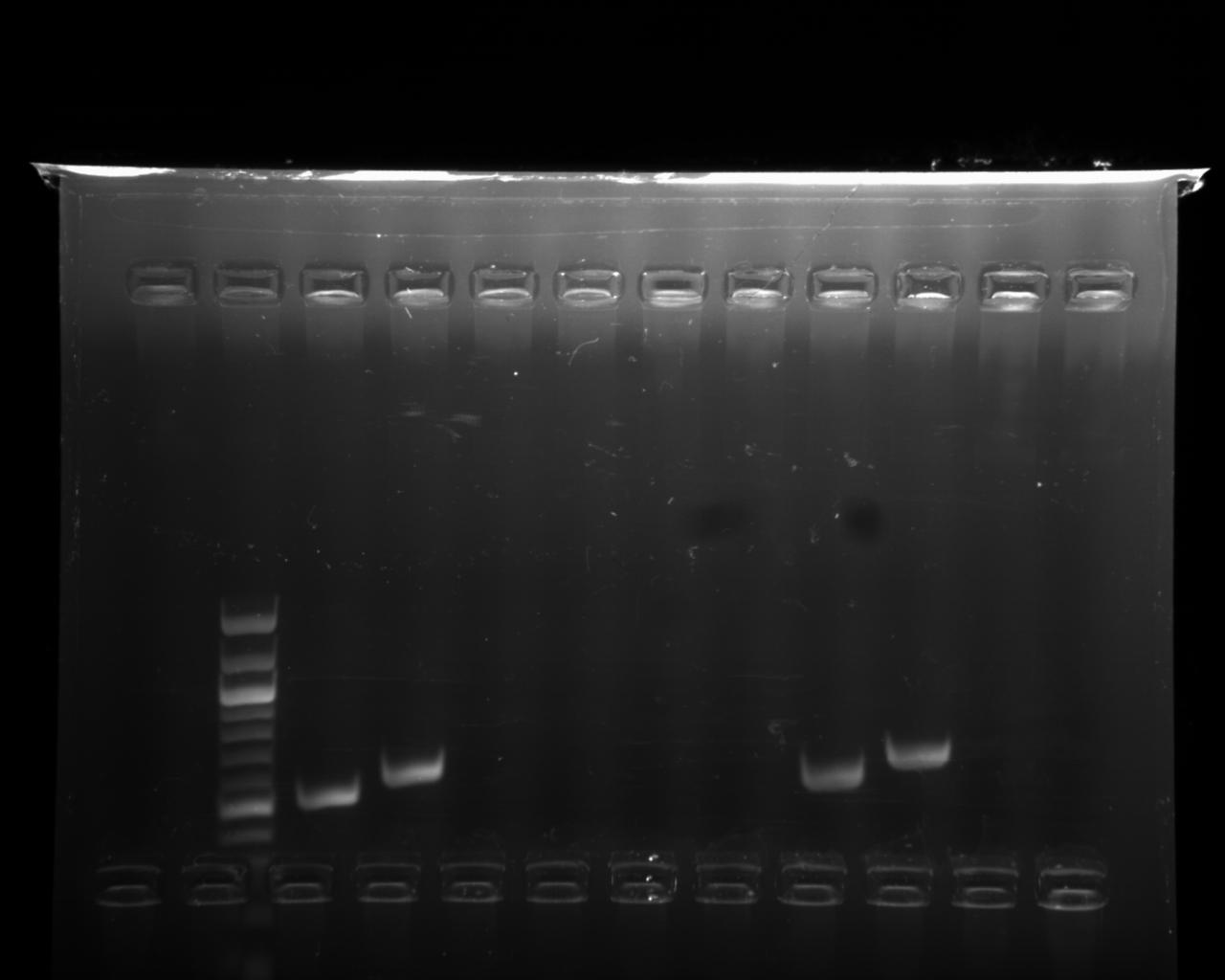

Supplement: Figure 7—source data 1. [file elife-84491-fig7-data1.zip › Figure 7- source data 1/Figure-7(B).jpg]

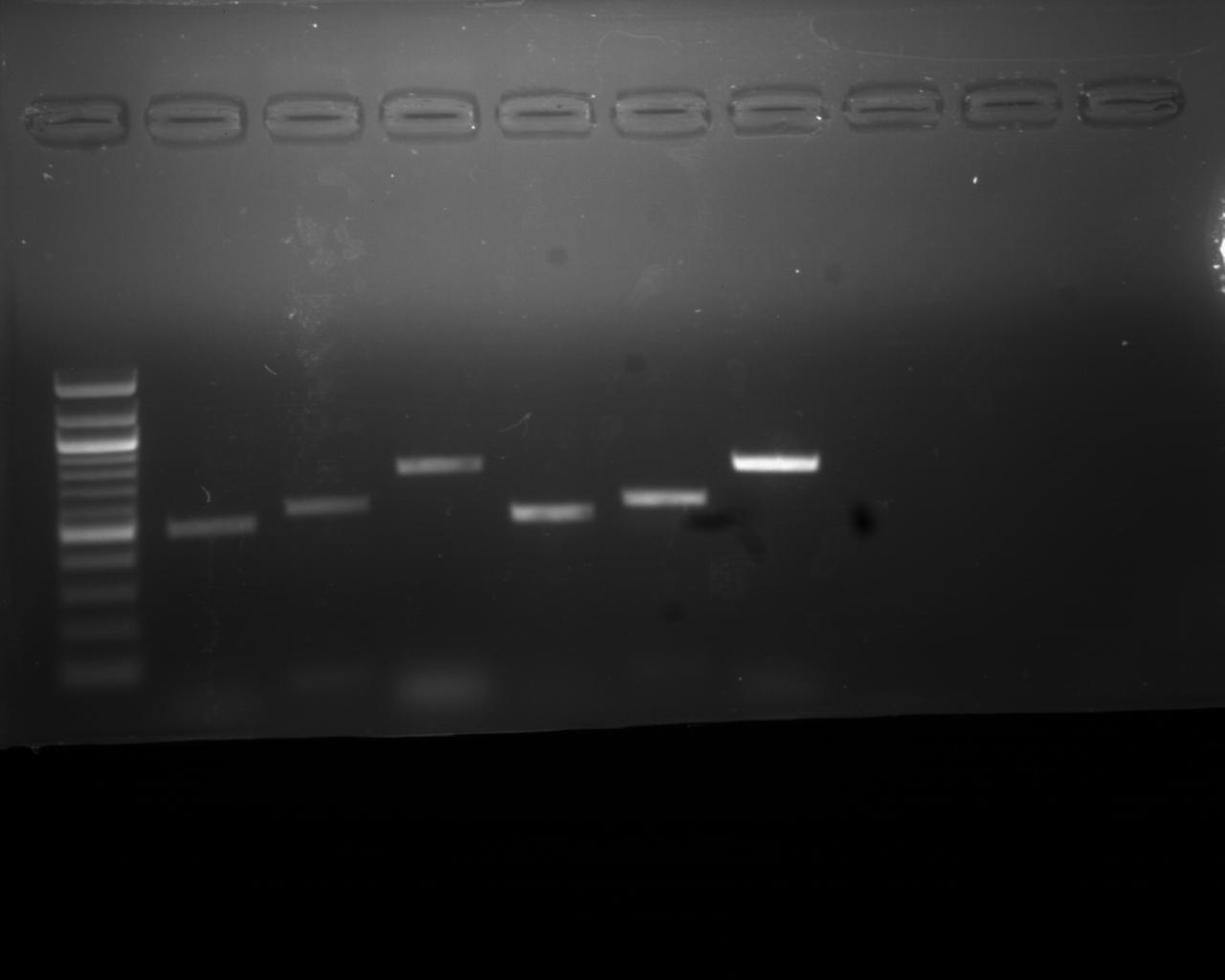

Supplement: Figure 7—source data 1. [file elife-84491-fig7-data1.zip › Figure 7- source data 1/Figure-7(C).jpg]
