# Supplementary material for: The Plasmodium falciparum apicoplast cysteine desulfurase provides sulfur for both iron-sulfur cluster assembly and tRNA modification
Source: eLife. 2023 May 11;12:e84491. doi: 10.7554/eLife.84491 (PMC10219651; doi:10.7554/eLife.84491)
Supplement: Figure 2—source data 1. [file elife-84491-fig2-data1.zip › Figure 2- source data 1/Figure 2- source data 1.pdf]

## Figure 2(A), Top

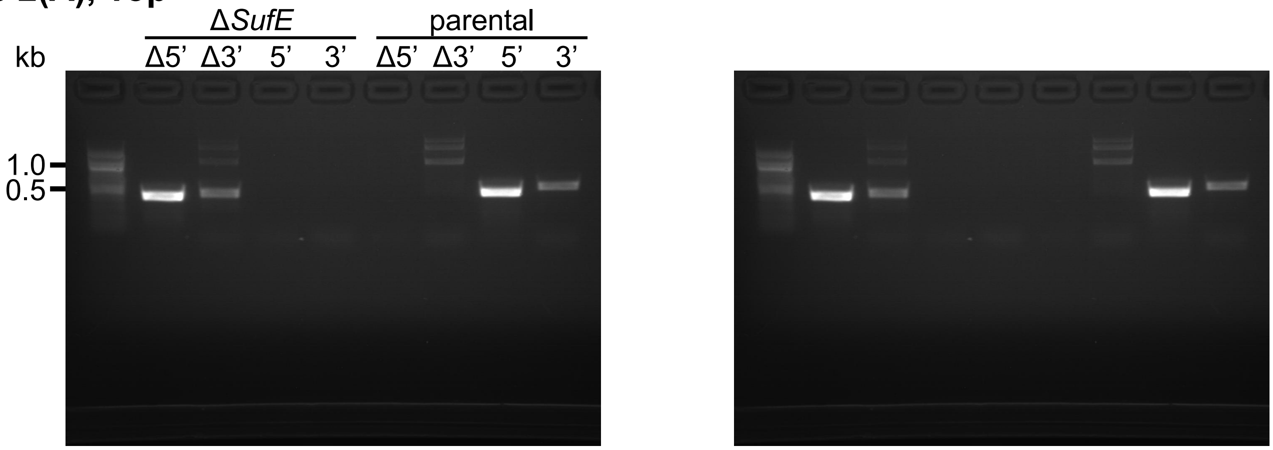


## Figure 2(A), Bottom

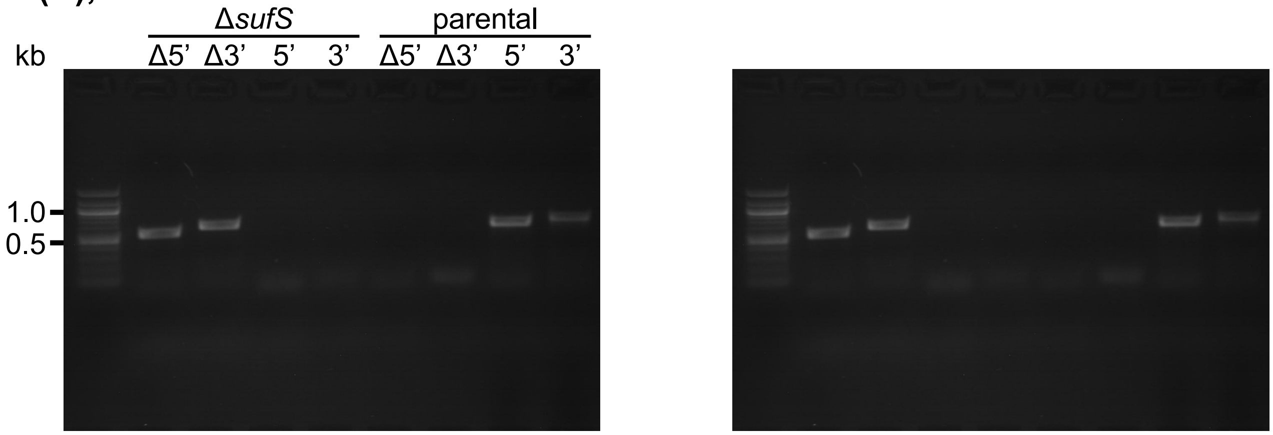


## Figure 2(B), Top

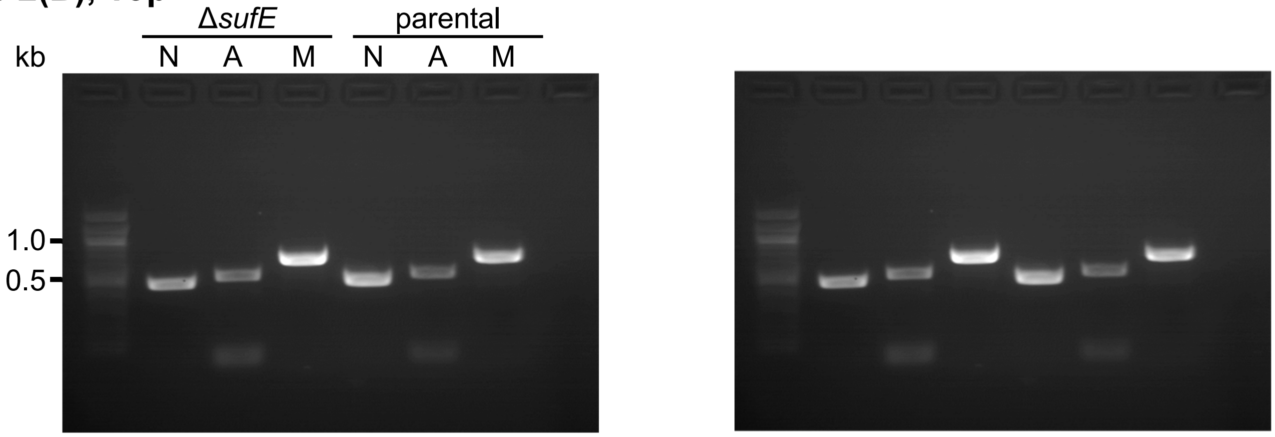


## Figure 2(B), Bottom

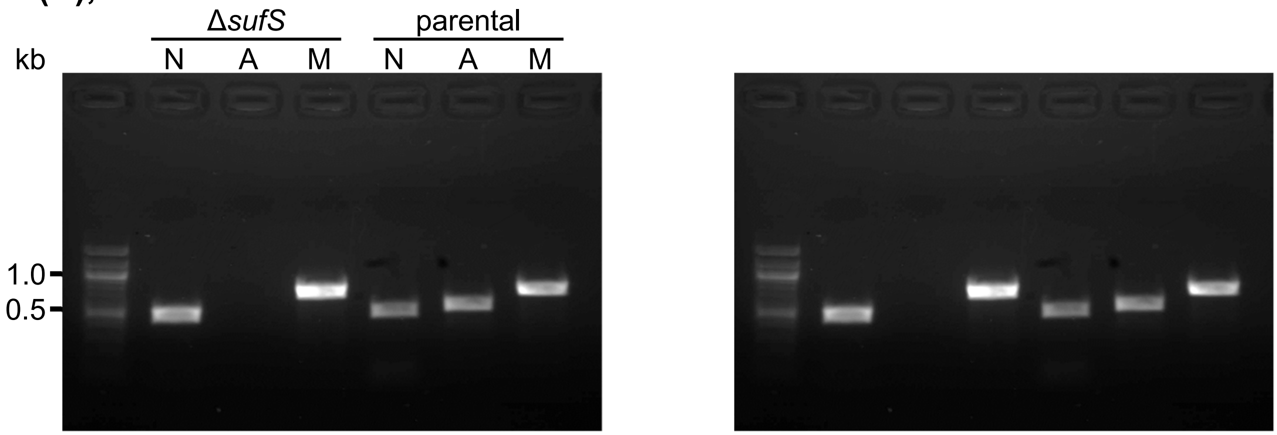


**Figure 2- source data 1.** Uncropped agarose gel images of PCR analyses presented in **Figures 2(A)** and **2(B).**
