# Supplementary material for: The Plasmodium falciparum apicoplast cysteine desulfurase provides sulfur for both iron-sulfur cluster assembly and tRNA modification
Source: eLife. 2023 May 11;12:e84491. doi: 10.7554/eLife.84491 (PMC10219651; doi:10.7554/eLife.84491)
Supplement: Figure 2—source data 1. [file elife-84491-fig2-data1.zip › Figure 2- source data 1/Figure 2- source data 1.pptx]

## Slide 1
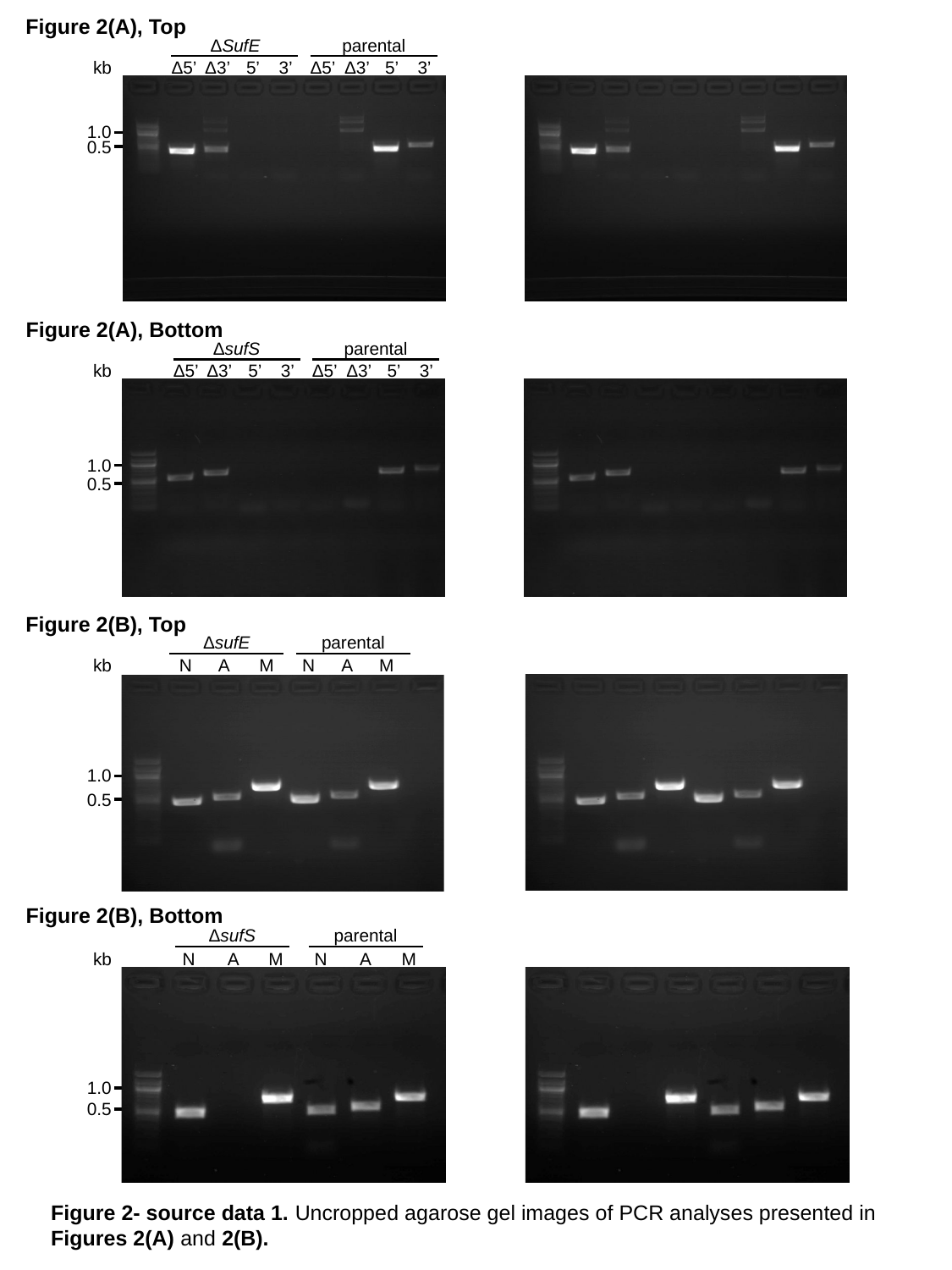

Figure 2(A), Top
ΔSufE
parental
kb
Δ5’
Δ3’
5’
3’
Δ5’
Δ3’
5’
3’
1.0
0.5
Figure 2(A), Bottom
ΔsufS
parental
kb
Δ5’
Δ3’
5’
3’
Δ5’
Δ3’
5’
3’
1.0
0.5
Figure 2(B), Top
ΔsufE
parental
kb
N
A
M
N
A
M
1.0
0.5
Figure 2(B), Bottom
ΔsufS
parental
kb
N
A
M
N
A
M
1.0
0.5
Figure 2- source data 1. Uncropped agarose gel images of PCR analyses presented in Figures 2(A) and 2(B).
