# Supplementary material for: The Plasmodium falciparum apicoplast cysteine desulfurase provides sulfur for both iron-sulfur cluster assembly and tRNA modification
Source: eLife. 2023 May 11;12:e84491. doi: 10.7554/eLife.84491 (PMC10219651; doi:10.7554/eLife.84491)
Supplement: Figure 3—source data 1. [file elife-84491-fig3-data1.zip › Figure 3 - source data 1/Figure 3- source data 1.pdf]

Figure 3(C)

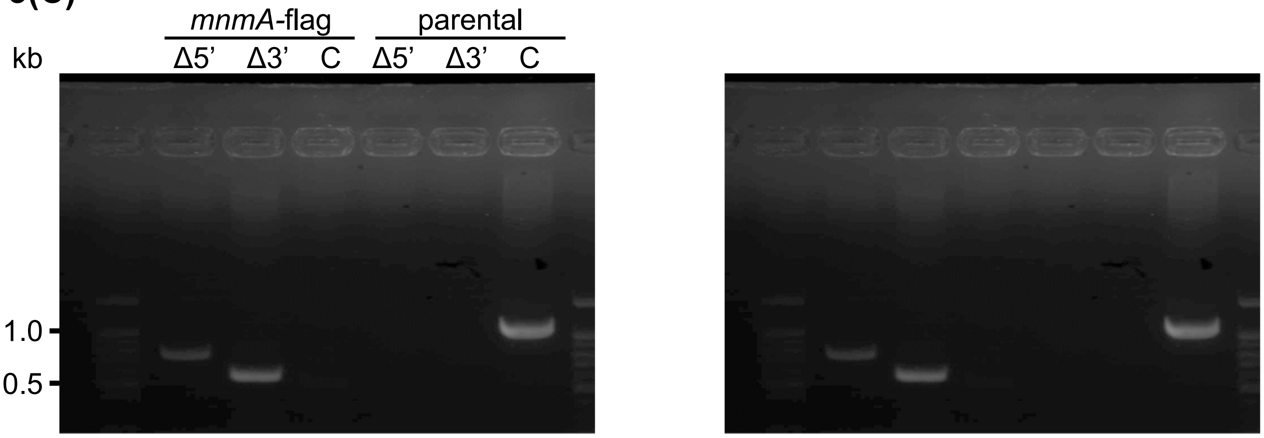

Figure 3(I)

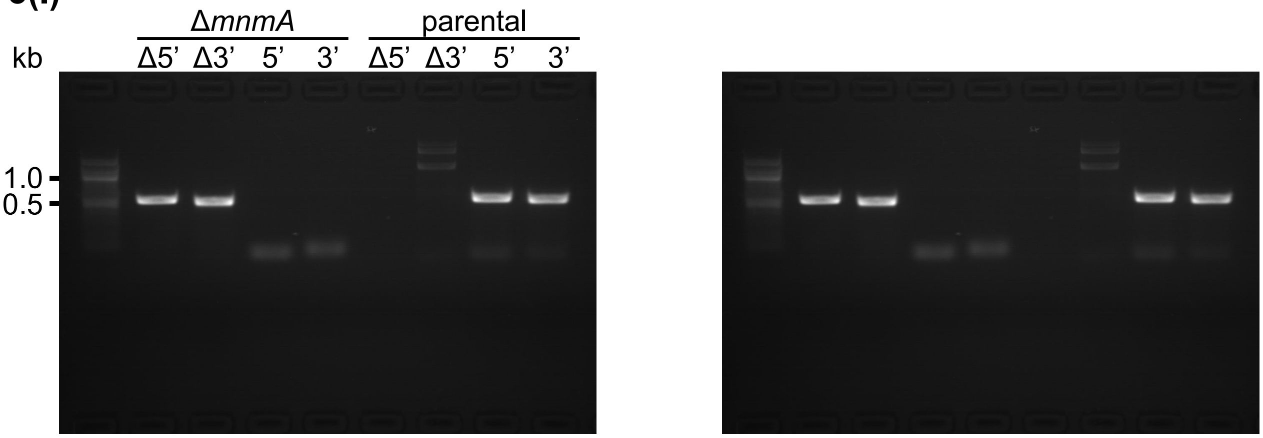

Figure 3(J)

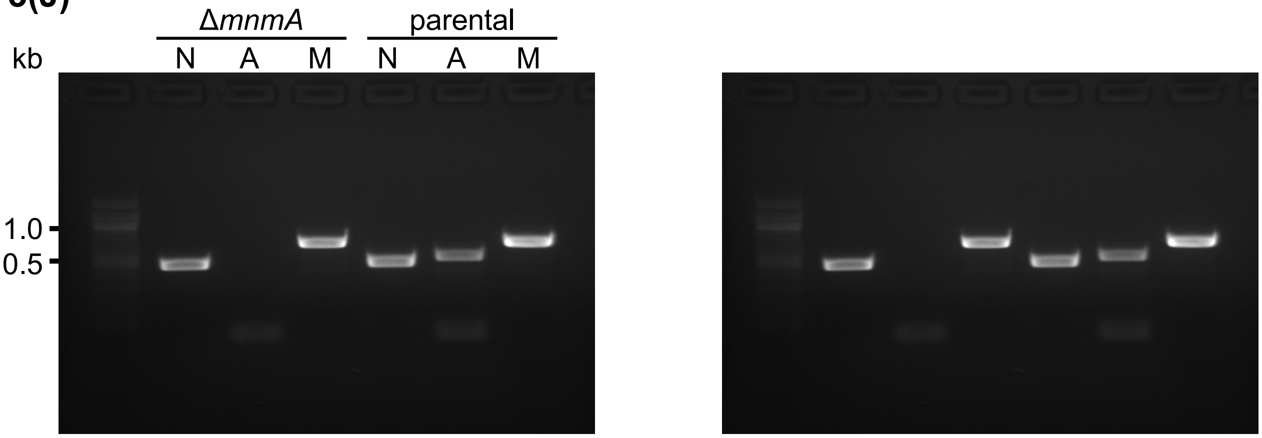

Figure 3- source data 1. Uncropped agarose gel images of PCR analyses presented in **Figures 3(C), 3(I),** and **3(J).**
