# Supplementary material for: The Plasmodium falciparum apicoplast cysteine desulfurase provides sulfur for both iron-sulfur cluster assembly and tRNA modification
Source: eLife. 2023 May 11;12:e84491. doi: 10.7554/eLife.84491 (PMC10219651; doi:10.7554/eLife.84491)
Supplement: Figure 3—source data 1. [file elife-84491-fig3-data1.zip › Figure 3 - source data 1/Figure 3- source data 1.pptx]

## Slide 1
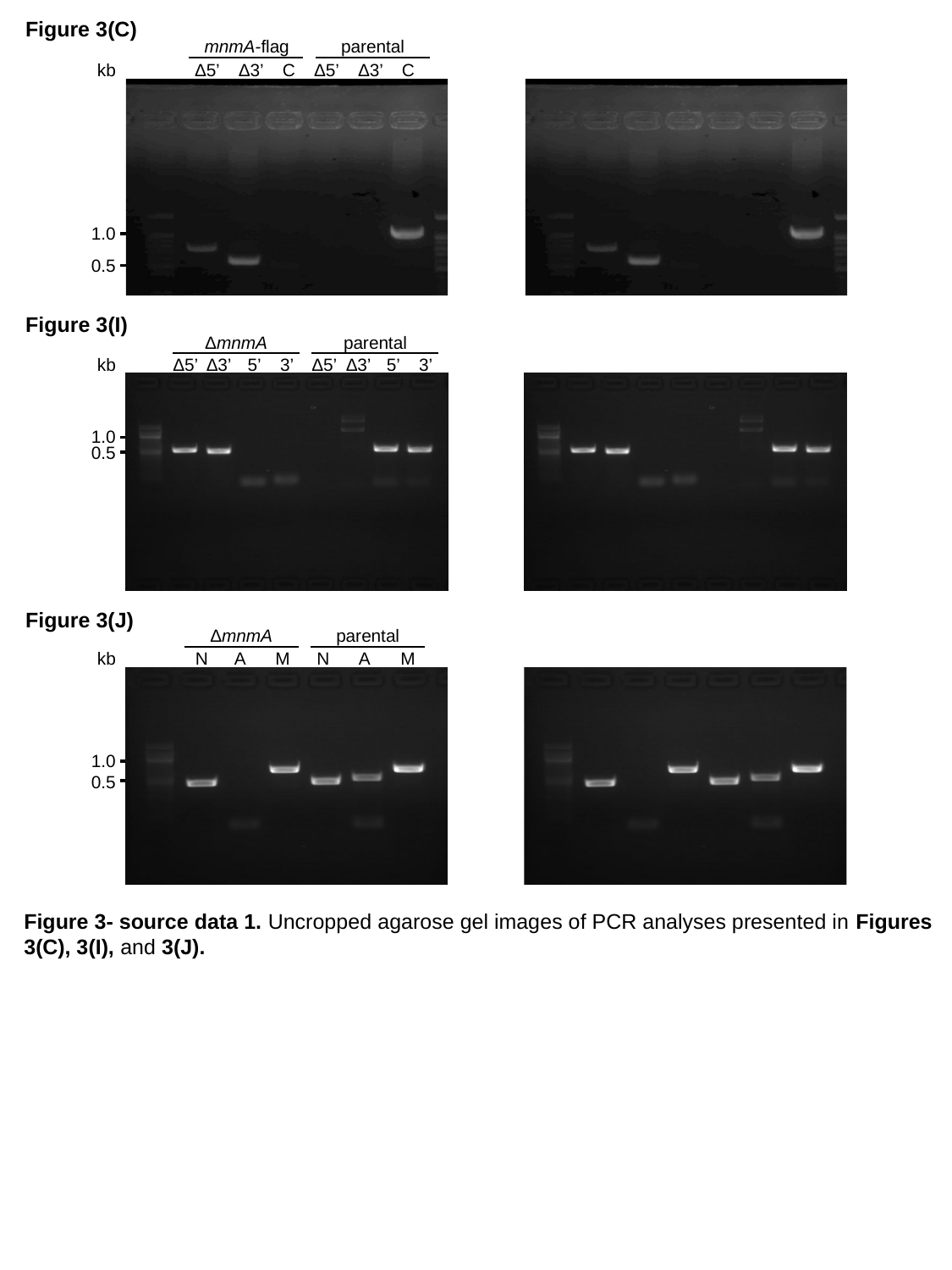

Figure 3(C)
mnmA-flag
parental
kb
Δ5’
Δ3’
C
Δ5’
Δ3’
C
1.0
0.5
Figure 3(I)
ΔmnmA
parental
kb
Δ5’
Δ3’
5’
3’
Δ5’
Δ3’
5’
3’
1.0
0.5
Figure 3(J)
ΔmnmA
parental
kb
N
A
M
N
A
M
1.0
0.5
Figure 3- source data 1. Uncropped agarose gel images of PCR analyses presented in Figures 3(C), 3(I), and 3(J).
