# Supplementary material for: The Plasmodium falciparum apicoplast cysteine desulfurase provides sulfur for both iron-sulfur cluster assembly and tRNA modification
Source: eLife. 2023 May 11;12:e84491. doi: 10.7554/eLife.84491 (PMC10219651; doi:10.7554/eLife.84491)
Supplement: Figure 3—figure supplement 1—source data 1. [file elife-84491-fig3-figsupp1-data1.pdf]

```
Pf MnmA  MLIFFFFFFFFKYIYNIFILTCFYITLSSYYFIISFIFSTLMFFYFCTFYVISLFFLYIS   60
Sy MnmA  ------------------------------------------------------------    0
Bs MnmA  ------------------------------------------------------------    0
Ec MnmA  ------------------------------------------------------------    0
At MnmA  ------------------------------------------------------------    0
Sc Mtu1  ------------------------------------------------------------    0

Pf MnmA  SFCKSIKVTQLYDKKIKIKSFINNYLVSCRKKKYIYNNVDDKSNIGTFNLYHNIRDNNNN  120
Sy MnmA  ------------------------------------------------------------    0
Bs MnmA  ------------------------------------------------------------    0
Ec MnmA  ------------------------------------------------------------    0
At MnmA  ------------------------------------------------------------    0
Sc Mtu1  ------------------------------------------------------------    0

Pf MnmA  NNNNDNNNNLKKRDDVLFPLCNKNIINDVQKIYDEVNNIKDKEQKINYLMEQCSSLCKEN  180
Sy MnmA  ------------------------------------------------------------    0
Bs MnmA  ------------------------------------------------------------    0
Ec MnmA  ------------------------------------------------------------    0
At MnmA  ------------------------------------------------------------    0
Sc Mtu1  ------------------------------------------------------------    0

Pf MnmA  YFPPILNLNKAYRNKRIDEFNKGNKNFYINEVGKNIWYKYVNRCDEILFMAIDIQIDEDE  240
Sy MnmA  ------------------------------------------------------------    0
Bs MnmA  ------------------------------------------------------------    0
Ec MnmA  ------------------------------------------------------------    0
At MnmA  ------------------------------------------------------------    0
Sc Mtu1  ------------------------------------------------------------    0

Pf MnmA  QRNNSIKDVHDVHDDNIKTCTLIKDDKHFEKYKDIHNDNILKNILPLDKKIDSIKNMLNH  300
Sy MnmA  ------------------------------------------------------------    0
Bs MnmA  ------------------------------------------------------------    0
Ec MnmA  ------------------------------------------------------------    0
At MnmA  ------------------------------------------------------------    0
Sc Mtu1  ------------------------------------------------------------    0

Pf MnmA  KYMKKKKCIITIDAYSNNLILYCFLYLILKHINKMYLYSFMNIQIKEITAKLKELFDLHF  360
Sy MnmA  ------------------------------------------------------------    0
Bs MnmA  ------------------------------------------------------------    0
Ec MnmA  ------------------------------------------------------------    0
At MnmA  ------------------------------------------------------------    0
Sc Mtu1  ------------------------------------------------------------    0

Pf MnmA  NVHHIIDYIHEYIYNFLMSYHIKRKKNKSKNMKEKDIKNVFANNIIISDEENKHISKESS  420
Sy MnmA  ---------------------------------------------------------MAT    3
Bs MnmA  ------------------------------------------------------------    0
Ec MnmA  ------------------------------------------------------------    0
At MnmA  ------------------------------------------------------------    0
Sc Mtu1  ------------------------------------------------------------   14

Pf MnmA  DMYKKKTTITTTTTTKKKKNTMKLFTYPRIAHMLSGGVDSLMALHLLERKKFYVDNYFFN  480
Sy MnmA  SPYPL-----------VAPEIAGSPSRPRIVAALSGGVDSSTVAAILHEQGYAVEGVTL-   51
Bs MnmA  --------------------MEKRPEDTRVVVGMSGGVDSSVAALLLKEQGYDVIGIFMK   40
Ec MnmA  ---------------------M-SETAKKVIVGMSGGVDSSVSAWLLQQQGYQVEGLFMK   38
At MnmA  ---------------------M-PEKPLRVAVLLSGGVDSSVALRLLHAAGHSCTAFYLK   38
Sc Mtu1  -MLARYLNLIGRRSASPYRPQRLPAKFDNVIVAMSSGVDSSVAAALFAGEFPNTRGVYMQ   59
```

```
Pf MnmA  -FTN---------A----DC-SKNDIKYVKDICKNNKRNLFIININDEYFDEVLVPMLFF  525
Sy MnmA  -WLMRG------KGQC---CT--DGLVDAAAICEQLGIPHHIVDSRELFQANIVDYLVAG   99
Bs MnmA  NWDDTD------ENGF---CTATEDYEDVIRVCNQIGIPYYAVNFEKQYYEKVFQYFLDE   91
Ec MnmA  NWEEDD------GEEY---CTAAADLADAQAVCDKLGIELHTVNFAAEYWDNVFELFLAE   89
At MnmA  IWFQEG------FENFWNQCPWEDDLKYAKHVCEQVDVPLEVVHLTDEYWERVVSYIIEE   92
Sc Mtu1  NWSESQSLDDPGKE----PC-YERDWRDVNRVAKHLNIRVDKVNFEQDYWIDVFEPMLRG  114

                     *
Pf MnmA  YADGKVPNPDIMCNQKIKYNFFLKVIKSIYKQKWNYRTKSKLCNYDFISTGHYAMIRTND  585
Sy MnmA  YADGITPLPCSQCNKLVKFGPLLTYARETL-------------GISQIATGHYARVRFNS  146
Bs MnmA  YKAGRTPNPDVLCNKEIKFKAFLEHA-LSL-------------GADYLATGHYARVD--R  135
Ec MnmA  YKAGRTPNPDILCNKEIKFKAFLEFAAEDL-------------GADYIATGHYVRRA--D  134
At MnmA  YRCGRTPNPDVLCNTRIKFGAFMDAI-SDM-------------EYDYVGSGHYAKVVHPP  138
Sc Mtu1  YSEGSTPNPDIGCNKFVKFGKLREWLDEKYG-----------TGNYWLVTGHYARVM--Q  161

Pf MnmA  KNNPNNIFNNNLFIKKKKKKIKNIKNKKNIKNKNNIKNKNNNNNIYTYNIYNLHNDNIKT  645
Sy MnmA  E-----------------------------------------------------------  147
Bs MnmA  S-----------------------------------------------------------  136
Ec MnmA  V-----------------------------------------------------------  135
At MnmA  AD----------------------------------------------------------  140
Sc Mtu1  EM----------------------------------------------------------  163

Pf MnmA  NYKKNNKYFYKLLVSNDKKKDQTFFLSSFNHIQLSKFIFPLSLYTKKDVKKYMNENNINN  705
Sy MnmA  ------LGRYQLLRAVDRQKDQSYFLYDLSQEHLAHSLFPLGNYTKAQTRQIAARYGLVT  201
Bs MnmA  ------GGKVRMLRGIDENKDQTYFLNQLTEDTLSKVMFPIGELQKSRVREIAKEAELAT  190
Ec MnmA  ------DGKSRLLRGLDSNKDQSYFLYTLSHEQIAQSLFPVGELEKPQVRKIAEDLGLVT  189
At MnmA  ----QNDASSVLELSQDMVKDQTYFLSHLSQTQLKRLLFPLGCVKKDEVRKLATQFDLPN  196
Sc Mtu1  ----NGKGLFHLLRSIYRPKDQSYYLSQINSTVLSSLLLPIGHLTKPEVRDLAKYAGLPT  219

                  *
Pf MnmA  YNHKETKGLCLFGN---IDMQTLLHKYFVNTEKDDIKNKQNEDNIFKENNILNLNNNFNQ  762
Sy MnmA  ANKPESQDLCLIET--YGSMRNFLDQHLGQRPGEIVDT-Q--------------------  238
Bs MnmA  ATKKDSTGICFIGE---RNFKTFLSQYLPAQPGDMMTM-D--------------------  226
Ec MnmA  AKKKDSTGICFIGE---RKFREFLGRYLPAQPGKIITV-D--------------------  225
At MnmA  KDRKDSQGICFLGK---IKFSDFVCRHIGEMEGIILEAES--------------------  233
Sc Mtu1  AEKPDSQGICFVNNSQHGKFKNFLKHYLPSSPGDIITVDPQS------------------  261

Pf MnmA  NEKKKKKEKKLLVDITTTSSHLKKFRETFIPKMNLHYKNYLINLDDQTILDINSDIHLYA  822
Sy MnmA  ------------------------------------------------GRVLGSHQGIHHYT  252
Bs MnmA  ------------------------------------------------GEVKGRHDGLMYYT  240
Ec MnmA  ------------------------------------------------GDEIGEHQGLMYHT  239
At MnmA  ------------------------------------------------GDFLGNHRGFWFYT  247
Sc Mtu1  -----------------------------------------------GAKTTWGRHDGLWSYT  277

Pf MnmA  IGQHKNVTNYLHNLYNKKMININGYKKKHVKNVISSFQWIVVYKKIKRDMSTNLIHNFIY  882
Sy MnmA  VGQRKGLGI-------------ASS-----------RPLYVVRIDAA--------MNRVV  280
Bs MnmA  IGQRHGLGI-------------GGS----------GEPWFAVGKDLE--------KNILY  269
Ec MnmA  LGQRKGLGI-------------GGTKE------GTEEPWYVVDKDVE--------NNILV  272
At MnmA  IGQRQGLRL-------------PG------------GPWYVVEKDTK--------NNVVF  274
Sc Mtu1  IGQKVGISM-------------PQADP------NYQGTWFVSEKLRD--------TNEIL  310

Pf MnmA  LTKNYDQDLFTHIRTKCKLHNIKWIEGKLPACIKKQFKKYNKINKKKKINNNNNKYKTN  942
Sy MnmA  VGEREEAT-----QAEAWVRQVNWVST-PAPD---------------------------  306
Bs MnmA  VDQGFHNPLL--YSDKITATNISWVRSDIMKG---------------------------  299
Ec MnmA  VAQGHEHPRL--MSVGLIAQQLHWVDREPFTG---------------------------  302
At MnmA  VSRNYYSIDK--RRRIFRVGSLRWNSGKPSGK---------------------------  304
Sc Mtu1  IVRGRDNPAL--YSDTMRIENFSSLGPREDTINA-------------------------  342
```

```
Pf MnmA  ETFHVYNNIQESGKKKKKKKVKNIPHDEKTIFVKIRNS---EQIKKAKIKFSLSNNTAYL    999
Sy MnmA  ----------E----------------PLAVEVQVRYR---TPAVPATLIPESPERVK-L    336
Bs MnmA  ----------E----------------EISCTAKFRYR---QEDHKVTVRMTGEGEAE-V    329
Ec MnmA  ---------------------------TMRCTVKTRYR---QTDIPCTVKALDDDRIE-V    331
At MnmA  ---------VR----------------ELRC--KVRHG---PGFYSCSFEMEGDGDVAVV    334
Sc Mtu1  --------FQN----------------TGALTLQFRSLQVPVQIKSCKLNRSADNLDITI    378

Pf MnmA  KVKQKDTGFSPGQIITLYFPFIIKKNNKVTYITNLNKYNNLINTNKNTIYYHCLGSATIS   1059
Sy MnmA  QFAEPQFGVTPGQAAVWYHGD------------------------------LLLGGGILE    366
Bs MnmA  IFDEQVRAVTPGQAVVFYDGE------------------------------ECLGGGTID    359
Ec MnmA  IFDEPVAAVTPGQSAVFYNGE------------------------------VCLGGGIIE    361
At MnmA  HLDEDDQGLAAGQFAAFYEGT------------------------------TCIGSGVIL    364
Sc Mtu1  HLASKQRAITPGQSCCLYIDD------------------------------RVLGSGPIS    408

Pf MnmA  NQFLDYNLYQHIKNI--HQIND----------LNMSK--------------------  1084
Sy MnmA  RPSP-----------------------------------------------------   370
Bs MnmA  DVYKDGTKLWYV---------------------------------------------   371
Ec MnmA  QRLPLPV--------------------------------------------------   368
At MnmA  ESWDDQCFPVCAKALQLAALEDKTKLGKPVKIMTMPTMSSVEADTGEASAEQKLVNA   421
Sc Mtu1  HVNNNDTHA------------------------------------------------   417
```

Figure 3- figure supplement 1- source data 1. Multiple sequence alignment used for **Figure 3(A)** and **Figure 3- figure supplement 1**.
