# Supplementary material for: The Plasmodium falciparum apicoplast cysteine desulfurase provides sulfur for both iron-sulfur cluster assembly and tRNA modification
Source: eLife. 2023 May 11;12:e84491. doi: 10.7554/eLife.84491 (PMC10219651; doi:10.7554/eLife.84491)
Supplement: Figure 3—figure supplement 2—source data 1. [file elife-84491-fig3-figsupp2-data1.pdf]

```
P. falciparum   ------------------------------------------------------------   0
B. microti      ------------------------------------------------------------   0
T. annulata     ------------------------------------------------------------   0
T. gondii       MFLSSLGGCKSLSAVLFLLLFVLDPRQASPLATSVASAAGSSAWLPAALPRSSGVRPPDQ  60
E. tenella      ------------------------------------------------------------   0

P. falciparum   ------------------------------------------------------------   0
B. microti      ------------------------------------------------------------   0
T. annulata     ------------------------------------------------------------   0
T. gondii       SRRLGRSAPRGNADNLQQLPSQNGVRRRRSVSWARGLPALFRAAADEGTEGGTRSDPRED 120
E. tenella      ------------------------------------------------------------   0

P. falciparum   --------------------------------------------MLIFFFFFFFFKYIY-  15
B. microti      ------------------------------------------------------------   0
T. annulata     ---------------------------------------MFKNVVL--LYNYLFFIFLNH  19
T. gondii       DACSLLPSSHRPSWQLRGDAAGASLNEKSGKTRRRSGSHPWVRSVAKHRFPRWTYTSLSH 180
E. tenella      ----------------------------------------------MRAFLELFFLVL--  12

P. falciparum   -------NIFILTCFYITLSSYYFIISFIFSTLMFFYFCTFYVISLFFLYISSFCKSIKV  68
B. microti      ------------------------------------------------------------   0
T. annulata     FN-----IIFVIHCNFAYSISY-------------------------NNK--FINN----  43
T. gondii       HRGVPCASLLPHRCGFCPSSSF-------------------------LNSARFCPVIGE 214
E. tenella      -------SALFHQCLEVSDSAI-------------------------LS-----------  29

P. falciparum   TQ-----------LYDKKIKI---------------------------K-SFINNY----  85
B. microti      ---------------------------------------MGT-----------G------   4
T. annulata     ------------------I------EI------LNKRNNISKNNNLLYPYLFIY------  67
T. gondii       DTAVRGVLAGGDGVWSHVV--SGSAARCDAGRPRQEKAGVAGRKRPHTAVFFISNFAPCG 272
E. tenella      -----CCHVQPARVWQPRRPLQLQVKICRTGRPLGG------SLMPRVS-FLLHPR-ANL  76

P. falciparum   --------------------LVSCRKKKYIYNNVDDKSNIGT------------------ 107
B. microti      -HRTICGCFVPANGHVRVAAT---------------------------------------  24
T. annulata     -NRIYNKCCKN-------------------------------------------------  77
T. gondii       ASTSSRDCFANSA------------------EDRRG-APVGPPLSPSLRSLSRSSLCSLS 313
E. tenella      LTRTIVGCFSRPLGPFCPSIDHKCDRQLYLKQVEAG-RPPEPPSAAS----QTADECASP 131

P. falciparum   -FNLYHNIRDNNNNNNNNDNNNNLKKRDDVLFPLCNKNIINDVQKIYDEVNNIKDKEQKI 166
B. microti      ------------------------------------------------------------  24
T. annulata     ------------------------------------------------------------  77
T. gondii       RSSLRSSARDPFSPATPLASSGSLPPRMRELLSELR--RQKDLHAVFE------------ 359
E. tenella      DGSVM--------------GS--------AFFEVAR--RWLTLCRVFRGA---PTAESRI 164

P. falciparum   NYLMEQ------------------------------------------------------ 172
B. microti      ------------------------------------------------------------  24
T. annulata     ------------------------------------------------------------  77
T. gondii       --------------KLVSFASSVPLYPARDASAVSSRASTASPPSPPRPVPSASLSPAPP 405
E. tenella      KILMHLAFQETNGSDFLSFTGKRPLTRIKQS----------------------AHGPSAS 202

P. falciparum   --------CSSLCKENYFPPILNLNKAYRNK--RID-EFNKGNKNFYINEVGKNIWYKYV 221
B. microti      NKGTNEELNVGNHDAHIQQTA--F---ARELNARLVERLSK-------KFSSSSEHLTEV  72
T. annulata     -----EPFRANSDTKHTLTST--LQHNYNDLNEKIKHRIESESSCLLEDNLNKSDFVQEL 130
T. gondii       PKATE---TSGRADDRLLPPAQLLQQPRREASEEAD------------EEGDSPEAWERV 450
E. tenella      SEALESGSAAGVDEASVC---------------PST------------SDLVLSDIWRPV 235

P. falciparum   NRCDEILFMAIDIQIDEDEQRNNSIKDVHDVHDDNIKTCTLIKDDKHFEKYKD------- 274
B. microti      T------RVASEI-FPNPPKDEH------------------FHL---------------F  92
T. annulata     A------RLGRSI-YSNIPTPTL------------------FKS-----VPNGINLLKSF 160
T. gondii       AGCAALVRIRVCLRRVLSPKREG-----------ESRACSVFREADVREEPQQ-GALASL 498
E. tenella      TGCAAVAAVKVSLHYPVRPQQRS-----------LAA----------------------- 261
```

```
P. falciparum   -------------------IHNDNILKNILPLDKKIDSIKNMLNHKYMKKKKCIITIDAY   315
B. microti      --NLSQFSPE--------FADQFGAKP-YAKLQDCITPI-YLAAIRYWHEGVERLAIDGT   140
T. annulata     --NSNDFNSLGCNGRDYFLVKSPVPVD-YKLLDDCSSSI-YISIH---LDKDKKIYLDGI   213
T. gondii       SVAASSSSGASCHDVE-------GAKKERRELRRE-DRD-GEDERR---RWELRVDLRGW   546
E. tenella      --SITSNSRLCCVAA----------------------------------KQGAKVHVHGE   285

P. falciparum   SNNLILYCFLYLILKHINKMYLYSFMNIQIKEITAKLKEL--------------------   355
B. microti      SNSLVGKTYLALMLSALHGNPLDELASIFE------------------SKGRTFEQLSRF   182
T. annulata     SDSFVFKGIISLLIS---------------------------------------------   228
T. gondii       SDSLVVRAWLAILVVGLNNAAPDTVLALSTDD---ILREAGLMPSSTPSGGKGHKDTVKE   603
E. tenella      SDSLVVGGLLQLLSRSLSGSAPCGVFALSALGSTKLLQFSSLLNLLPDSRVRGFRTAMHI   345

P. falciparum   ------------------------------------------------------------   355
B. microti      TDGISQRICQ-----------ELFR-----------------------------------   196
T. annulata     ------------------------------------------------------------   228
T. gondii       TQGEPEKQTEEGEDGHREAEEEAEEAEEEAEEEEEEEEEKERKGSKRESEEERLQEGEER   663
E. tenella      LMGSTQDLLQR------YFCNEVLPP--PAELPQ---NAHGEQGTAP--NKQDITTG---   389

P. falciparum   --------------------FDLHFNVHHIIDYIHEYIYN--------------------   375
B. microti      ------------------------------------------------------------   196
T. annulata     ------------------------------------------------------------   228
T. gondii       LREEETRKCREEEKRRLVVPQGLEFMLRSIQRQVREQLSRLAEEEKNGGAPDGKVRKSKT   723
E. tenella      -------------RTQAKVPKGTSL------------------------------RP---   403

P. falciparum   ------FLMSYHIK---------------RKKNKSKNMKEKDIKNVFANN-----IIISD   409
B. microti      -------LIGFNTNKA--AGYDD-------SEADMDKNDKFQFKRLVPK------PAL--   232
T. annulata     -------LIGWKNSTE--VEVDN-------V-----KKHKF-FKKVIDH------GIISE   260
T. gondii       DRDASRDLTGETRTRTDENGVQRSVFHRCRSDAET-EADSHHVSSSLSSSLPPSQPHLSS   782
E. tenella      PRPANDALSGY------------------RQE-----PHGDQWGNPIPQHALPSI-----   435

P. falciparum   EENKHISKE-------------SSDMYKKKTTITTTTTTKKKKNTMKLFTYPRIAHMLSG   456
B. microti      ----------------------RNSLYRRSY---GFEVHDLYRLRSKSSSPNKVAMLISG   267
T. annulata     NSLNSIL------------NHIKNELKKQDN---PTKGPKRTSMFQSPRSKKRVAVLVSG   305
T. gondii       PSSSSLSSSSSPSSSSSSSSSAAPDLFASTT---ETCEEKRELRRSAASSPPQVAVLLSG   839
E. tenella      PRSKEIAK-----------------L----------AAKSRVSLPLSDGAGAEVHVLVSG   468

P. falciparum   GVDSLMALHLLERKKFYVDNYFFNFT-----------------------------NADC-   486
B. microti      GVDSSVALWMLKSQGYNVHAFYLKAFG--------------------------SDSPGCT   301
T. annulata     GVDSSLALWIMKSRGFDVQAFHLKVNDL------------------------SNGPGLC-   340
T. gondii       GVDSSVSLCLLQQRGFAPQAFFIKVWLPELLLVSRHLNRLLD------SGLAPAAAGGCG   893
E. tenella      GVDSAVSLLLMREWGFRPKPIFLKVWAPEAAQMKEQLQGRYQQEKLDIAAASATAAAACP   528
                                                                   *
P. falciparum   SKNDIKYVKDICKNNKRNLFIININDEYFDEVLVPMLFFYADGKVPNPDIMCNQKIKYNF   546
B. microti      VAEDIRYATDCCNVLGVPLHILPVEDVYNRAVMQYMVEGYRAGNTPNPDVICNREVKFGY   361
T. annulata     SSNDISYAMEVCNILRVTLHVLPFSQVYDKHILSDVLDDYRKGEVPNPDILCNSRIKFGE   400
T. gondii       WERDLLFADQVCRQARVPLEVLPLQEAYWEGVVQQMLDEARQGLTPNPDWWCNQRVKFGA   953
E. tenella      WREDAKAAAAVAAAAGLSLEVVPMQQQYWDRVISGFLEGARAGLTLNPDWSCNSSVKFGA   588

P. falciparum   FLKVIKSIYKQKWNYRTKSK---------------------LCNYDFISTGHYAMIRTND   585
B. microti      FLNLATS-E---FGF------------------------------DMVASGHYARINPQY   387
T. annulata     FLKMA-T-D---WGF------------------------------DYVATGHYATLCDDF   425
T. gondii       FLDLLDGRE---TRFSARRIAGESEGENEKEEADMPFLRNSSRWTGAVASGHYARVVPAA  1010
E. tenella      FAETLDAVD---YR---------------------------------IASGHYARILI--   610

P. falciparum   KNNPNNIFNNNLFIKKKKKKIKNIKNKKNIKNKNNIKNKNNNNNIYTYNIYNLHNDNIKT   645
B. microti      FIPPQQSNG---------------------------------------------------   396
T. annulata     FTKEQIKSP------------S------YL------DGC---------------------   440
T. gondii       ETSRRSEEG------------E------DT------EDRDEDG----------EEDRGDK  1036
E. tenella      ------------------------------------------------------------   610
```

```
P. falciparum   NYKKNNKYFYKLLVSNDKKKDQTFFLSSFNHIQLSKFIFPLSLYTKKDVKKYMNENNINN  705
B. microti      -TQSSFVRFNELETSYYVNKDQTYFLSRLSSAQLGKMICPLGVHKKKMVRALAYINGLGT  455
T. annulata     TSINGYYRIKRLCLSTDTVKDQTYFLSRLNQNQMSRLIFPIGNILKTQVRDFAKTVGLPT  500
T. gondii       ERGSEEERRTRLFRGKDRRKDQSYFLSGLSQRQLRRLVTPVGDMEKVEVRRLAAALDLPT 1096
E. tenella      -----DEGCPRLLRGVDLTKDQSYFLSGLSSLQLSRLLFPVGALLKTQVRKIAVSWGLPS  665

                         *
P. falciparum   YNHKETKGLCLFGNIDMQTLLHKYFVNTEKDDIKNKQNEDNIFKENNILNLNNNFNQNEK  765
B. microti      QARDDSMGVCFLGRLDLRQFLKAHLGETPGQIIDYKS-KQVIGQHTGLYNY----TIGQR  510
T. annulata     YNKKDSFGLCFLEDLDLSEYLTKTLGESRGPIVEYET-NKVIGEHNGLYNF----TIGQK  555
T. gondii       ARRQDSQGLCFLGNLSLSFFFRHFLGSSTGPVLHFPS-CLALGSHDGLWNF----TVGQR 1151
E. tenella      SQRPDSQGLCFLGPLPVSQFLMHLLGEEEGPVIHFPT-GVSIGRHKGLWGF----TVGQQ  720

P. falciparum   KKKKEKKLLVDITTTTSSHLKKFRE-TFIP------------------------------  793
B. microti      Q---GLGVSIDHGG-EE-------------------------------------------  523
T. annulata     K---TINNYLNPKHVGN-------------------------------------------  569
T. gondii       K---GVTPCIDVARVRR-LSSLPDSSLTPHAASVDSEGSEEQTARAGDSRRRQARAPPGK 1207
E. tenella      K---GVVPLLDPRLCRR-CHGASG------------------------------------  740

P. falciparum   ------------------------------------KMNLHYKNYLINLDDQTILDINSD  817
B. microti      ------------------------------------------------------------  523
T. annulata     ------------------------------------------------------------  569
T. gondii       PTKASATGQGRQAADDEERRLLEAKCHRDGDDSCLRSANHTVRGRLECTDNADRP--DGR 1265
E. tenella      ------------------------------------------------------------  740

P. falciparum   IHLYAIG------------------QHK---------------NVTNYLHNLYNKKMINI  844
B. microti      ------------------------------------------------------------  523
T. annulata     ------------------------------------------------------------  569
T. gondii       VPVLSEGNEAPSSSSCSSSAEADEGQEEGGDRQSRLSSSSFAACLSGSPQNLFE----PA 1321
E. tenella      ------------------------------------------------------------  740

P. falciparum   NGYKKKHVKNVISSFQWIVVYKKIKRDM-----------------------------ST-  874
B. microti      --------------GGWFVVSKDAASNTLYVTKEYNSRHFTGRSCIRRAFSLAHVRWNN-  568
T. annulata     --------------TPKYVVKKDIYKNTLYVSASYNEDFFTNPRGIRSSFKVRDFFWNT-  614
T. gondii       NPFSRTGLGAASLAGRWVVAAKHPPSNALFVVSEKEMKAAAA---VAES-----VGYSLD 1373
E. tenella      --------GPPKLSGPWSVVGKLPEANALFVVSKEEEAAAED---RLRALATEPGNYSEP  789

P. falciparum   -----NLIHNFIYLTKNYDQD-LFTHIRTKCKLHNIKWIEGKLPACIKKQFKKYNKINK-  927
B. microti      -----PILLNSHGINEAD----YI----------------------------------KA  585
T. annulata     -----PDYKDLINKVQSEVSD-NFKFK--------VKLRHTPNYNYCNINFKGDEATDLQ  660
T. gondii       VLAAGPGVRT-LGDSQTYLLALLLTLQQKFLRVDNIQWISHPPCAACNAEEQPVS----- 1427
E. tenella      L-G-APLLRAAANGDKAAALALKLRQLRTCLRVDNIRWFHGKAPAGFDASATLQP-GCLR  846

P. falciparum   ---------------KKKKINNNNNKYKTNETFHVYNNIQESGKKKKKKKVK--NIPHDE  970
B. microti      EWEPPITRRDI-KTMQDLTLDRNVASTE----KLFL----K---------VRNGTVLYEA  627
T. annulata     KWLPTTYELAA-ATIASV--DNSKCSIKM--IQLILT-FCT---------IEDVGFIHTA  705
T. gondii       -RDPSFLGTLLGSRDPRARLRGDEKSLETADEFAFLR-WALEGSRDKRPRLYDVQVRHAA 1485
E. tenella      EEGPTL-AAVAASAVAAAKT-NTALPMERP-AESFL-----TG-SSKGGAGFVVQVRHSA  897

P. falciparum   KTIFVKIRNS--------------------------EQIKKAK----------IKFSLSN  994
B. microti      ELK------------------------------------------LSPPI---RKGQGVT  642
T. annulata     DSKATTIAL---F------------------------------FIFGSNK---DEKDGTS  729
T. gondii       GTACAAIHRRVRLCLFPPERRSPSFLSPSAVSEESGGRRKESRAPFGPSRVETSAGSSGA 1545
E. tenella      GFHGVAKHNFKVLCVSRGH----------------------------------SGNSSPI  923

P. falciparum   NTAYLKVKQKDTGFSPGQIITLYFPFIIKKNNKVTYITNLNKYNNLINTNKNTIYYHCLG 1054
B. microti      QHGWAVLDRSDPGLATGQYAALYRGN------------------------------ICIG  672
T. annulata     IELEDIVHEPAPSEHGSKKSPSELHI------------------------------YEIG  759
T. gondii       WTAWIELAEPDEGLAPGQIAAIYEGE------------------------------ECLG 1575
E. tenella      EEAELLLEEPDVGLAPGQVAAFYRDD------------------------------ECIG  953
```

```
P. falciparum   SATISNQFLDYNLYQHIKN------------------------IHQINDLNMS---K--- 1084
B. microti      SGIIYQSF---------------------------------------------------- 680
T. annulata     KPIDYLDWKQHKRTNSSQSGRNGTHGKTHTEKRRNSDPTKRSKVHKPQRITSELEQKTKP 819
T. gondii       AGRISARQGQMAVEAAL--RSAGLN----------------------------------- 1598
E. tenella      SGRISALQGSFALKSII--DRLG------------------------------------- 974

P. falciparum   ------------------------------------------------------------ 1084
B. microti      ------------------------------------------------------------ 680
T. annulata     NSPQQTTTKQQTNTKEQNTNKQQSSVEQKTKADQSGPITRPFISPSVLKPSEKAERVFSS 879
T. gondii       ------------------------------------------------------------ 1598
E. tenella      ------------------------------------------------------------ 974

P. falciparum   ------------------------------------------------------------ 1084
B. microti      ------------------------------------------------------------ 680
T. annulata     NEKPAVQQRLFKPRENKYQKRTERYDTDMDFEKIFNPVHENAKDDEPGSYMPFESIYDLS 939
T. gondii       ------------------------------------------------------------ 1598
E. tenella      ------------------------------------------------------------ 974

P. falciparum   ------------------------------------------------------------ 1084
B. microti      ------------------------------------------------------------ 680
T. annulata     LDKCRLNKAFPPKGKPAGHKFECIDLDVIRVSKDFKNISLVNANVIHPKSNTHKICKISF 999
T. gondii       ------------------------------------------------------------ 1598
E. tenella      ------------------------------------------------------------ 974

P. falciparum   ------------------------------------------------------------ 1084
B. microti      ------------------------------------------------------------ 680
T. annulata     DNKVLVDKVDDSMEFTAVVLEHVRSISILSVISTNPNMERSAIQFVITGKDKINVMNDYV 1059
T. gondii       ------------------------------------------------------------ 1598
E. tenella      ------------------------------------------------------------ 974

P. falciparum   -----------     1084
B. microti      -----------      680
T. annulata     SVMSTILKSEL     1070
T. gondii       -----------     1598
E. tenella      -----------      974
```

Figure 3- figure supplement 2- source data 1. Multiple sequence alignment used for **Figure 3- figure supplement 2**.
