# Supplementary material for: The Plasmodium falciparum apicoplast cysteine desulfurase provides sulfur for both iron-sulfur cluster assembly and tRNA modification
Source: eLife. 2023 May 11;12:e84491. doi: 10.7554/eLife.84491 (PMC10219651; doi:10.7554/eLife.84491)
Supplement: Figure 3—figure supplement 3—source data 2. [file elife-84491-fig3-figsupp3-data2.pdf]

```
Sy MnmA   -----------MATSPY---PLVA----PEIAGSPSRPRIVAALSGGVDSSTVAAILHEQ     42
Bs MnmA   ------------------------------MEKRPEDTRVVVGMSGGVDSSVAALLLKEQ     30
Ec MnmA   --------------------------------MSETAKKVIVGMSGGVDSSVSAWLLQQQ     28
At MnmA   --------------------------------MPEKPLRVAVLLSGGVDSSVALRLLHAA     28
Sc Mtu1   MLARYLNLIGRRSASPY---RP--------QRLPAKFDNVIVAMSSGVDSSVAAALFAGE     49
Pf MnmA   -----------MYKKKTTITTTTTTKKKKNTMKLFTYPRIAHMLSGGVDSLMALHLLERK    470
Bm MnmA   ----------SLYRRSY---GFEVHDLYRLRSKSSSPNKVAMLISGGVDSSVALWMLKSQ    281
Ta MnmA   ----------ELKKQDN---PTKGPKRTSMFQSPRSKKRVAVLVSGGVDSSLALWIMKSR    319
Tg MnmA   ----------DLFASTT---ETCEEKRELRRSAASSPPQVAVLLSGGVDSSVSLCLLQQR    853
Et MnmA   ----------------------AAKSRVSLPLSDGAGAEVHVLVSGGVDSAVSLLLMREW    482

Sy MnmA   GYAVEGVTL--WLMRG-------------------------KGQCCT--DGLVDAAAICE     73
Bs MnmA   GYDVIGIFMKNWDDTD-------------------------ENGFCTATEDYEDVIRVCN     65
Ec MnmA   GYQVEGLFMKNWEEDD-------------------------GEEYCTAAADLADAQAVCD     63
At MnmA   GHSCTAFYLKIWFQEGFE---------------------NFWNQ-CPWEDDLKYAKHVCE     66
Sc Mtu1   FPNTRGVYMQNWSESQSL--------------------DDPGKEPCY-ERDWRDVNRVAK     88
Pf MnmA   KFYVDNYFFNF------------------------------TNADC-SKNDIKYVKDICK    499
Bm MnmA   GYNVHAFYLKAFG--------------------------SDSPG-CTVAEDIRYATDCCN    314
Ta MnmA   GFDVQAFHLKVNDL-------------------------SNGPGLC-SSNDISYAMEVCN    353
Tg MnmA   GFAPQAFFIKVWLPELLLVSRHLNRLLD------SGLAPAAAGG-CGWERDLLFADQVCR    906
Et MnmA   GFRPKPIFLKVWAPEAAQMKEQLQGRYQQEKLDIAAASATAAAA-CPWREDAKAAAAVAA    541

                                                 *
Sy MnmA   QLGIPHHIVDSRELFQANIVDYLVAGYADGITPLPCSQCNKLVKFGPLLTYARETLG--I    131
Bs MnmA   QIGIPYYAVNFEKQYYEKVFQYFLDEYKAGRTPNPDVLCNKEIKFKAFLEHA-LSLG--A    122
Ec MnmA   KLGIELHTVNFAAEYWDNVFELFLAEYKAGRTPNPDILCNKEIKFKAFLEFAAEDLG--A    121
At MnmA   QVDVPLEVVHLTDEYWERVVSYIIEEYRCGRTPNPDVLCNTRIKFGAFMDAI-SDME--Y    123
Sc Mtu1   HLNIRVDKVNFEQDYWIDVFEPMLRGYSEGSTPNPDIGCNKFVKFGKLREWLDEKYGTGN    148
Pf MnmA   NNKRNLFIININDEYFDEVLVPMLFFYADGKVPNPDIMCNQKIKYNFFLKVIKSIYKQKW    559
Bm MnmA   VLGVPLHILPVEDVYNRAVMQYMVEGYRAGNTPNPDVICNREVKFGYFLNLATSEFG--F    372
Ta MnmA   ILRVTLHVLPFSQVYDKHILSDVLDDYRKGEVPNPDILCNSRIKFGEFLKMA-TDWG--F    410
Tg MnmA   QARVPLEVLPLQEAYWEGVVQQMLDEARQGLTPNPDWWCNQRVKFGAFLDLL-DGRE--T    963
Et MnmA   AAGLSLEVVPMQQQYWDRVISGFLEGARAGLTLNPDWSCNSSVKFGAFAETL-DAVD--Y    598

Sy MnmA   SQ--------------------------------IATGHYARVRFN--------------    145
Bs MnmA   DY--------------------------------LATGHYARVD----------------    134
Ec MnmA   DY--------------------------------IATGHYVRRA----------------    133
At MnmA   DY--------------------------------VGSGHYAKVVHPPADQN---------    142
Sc Mtu1   YW--------------------------------LVTGHYARVMQEM-------------    163
Pf MnmA   NYRTKS---------------------KLCNYDFISTGHYAMIRTNDKNNPNNIFNNNLF    598
Bm MnmA   DM--------------------------------VASGHYARINPQYFIPPQQSNG----    396
Ta MnmA   DY--------------------------------VATGHYATLCDDFFTKEQIKSPS---    435
Tg MnmA   RFSARRIAGESEGENEKEEADMPFLRNSSRWTGAVASGHYARVVPAAETSRRSEEGE---   1020
Et MnmA   R---------------------------------IASGHYARILID--------------    611

Sy MnmA   ---------------------------------------------------SELGRYQLL    154
Bs MnmA   ---------------------------------------------------RSGGKVRML    143
Ec MnmA   ---------------------------------------------------DVDGKSRLL    142
At MnmA   -----------------------------------------------------DASSVLE    149
Sc Mtu1   ---------------------------------------------------NGKGLFHLL    172
Pf MnmA   IKKKKKKIKNIKNKKNIKNKNNIKNKNNNNNIYTYNIYNLHNDNIKTNYKKNNKYFYKLL    658
Bm MnmA   ------------------------------------------------TQSSFVRFNELE    408
Ta MnmA   ---------------------YLDGC---------------------TSINGYYRIKRLC    453
Tg MnmA   ---------------------DTEDRDED----------GEEDRGDKERGSEEERRTRLF   1049
Et MnmA   -----------------------------------------------------EGCPRLL    618
```

```
Sy MnmA    RAVDRQKDQSYFLYDLSQEHLAHSLFPLGNYTKAQTRQIAARYGLVTANKPESQDLCLIE    214
Bs MnmA    RGIDENKDQTYFLNQLTEDTLSKVMFPIGELQKSRVREIAKEAELATATKKDSTGICFIG    203
Ec MnmA    RGLDSNKDQSYFLYTLSHEQIAQSLFPVGELEKPQVRKIAEDLGLVTAKKKDSTGICFIG    202
At MnmA    LSQDMVKDQTYFLSHLSQTQLKRLLFPLGCVKKDEVRKLATQFDLPNKDRKDSQGICFLG    209
Sc Mtu1    RSIYRPKDQSYYLSQINSTVLSSLLLPIGHLTKPEVRDLAKYAGLPTAEKPDSQGICFVN    232
Pf MnmA    VSNDKKKDQTFFLSSFNHIQLSKFIFPLSLYTKKDVKKYMNENNINNYNHKETKGLCLFG    718
Bm MnmA    TSYYVNKDQTYFLSRLSSAQLGKMICPLGVHKKKMVRALAYINGLGTQARDDSMGVCFLG    468
Ta MnmA    LSTDTVKDQTYFLSRLNQNQMSRLIFPIGNILKTQVRDFAKTVGLPTYNKKDSFGLCFLE    513
Tg MnmA    RGKDRRKDQSYFLSGLSQRQLRRLVTPVGDMEKVEVRRLAAALDLPTARRQDSQGLCFLG   1109
Et MnmA    RGVDLTKDQSYFLSGLSSLQLSRLLFPVGALLKTQVRKIAVSWGLPSSQRPDSQGLCFLG    678

Sy MnmA    T--YGSMRNFLDQHLGQRPGEIVDTQGR--------------------------------    240
Bs MnmA    E---RNFKTFLSQYLPAQPGDMMTMDGE--------------------------------    228
Ec MnmA    E---RKFREFLGRYLPAQPGKIITVDGD--------------------------------    227
At MnmA    K---IKFSDFVCRHIGEMEGIILEAESG--------------------------------    234
Sc Mtu1    NSQHGKFKNFLKHYLPSSPGDIITVDPQSG------------------------------    262
Pf MnmA    N---IDMQTLLHKYFVNTEKDDIKNKQNEDNIFKENNILNLNNNFNQNEKKKKKEKKLLV    775
Bm MnmA    R---LDLRQFLKAHLGETPGQIIDYKSK--------------------------------    493
Ta MnmA    D---LDLSEYLTKTLGESRGPIVEYETN--------------------------------    538
Tg MnmA    N---LSLSFFFRHFLGSSTGPVLHFPSC--------------------------------   1134
Et MnmA    P---LPVSQFLMHLLGEEEGPVIHFPTG--------------------------------    703

Sy MnmA    -----------------------------------VLGSHQGIHHYTVGQRKGLGIASS-    264
Bs MnmA    -----------------------------------VKGRHDGLMYYTIGQRHGLGIGGS-    252
Ec MnmA    -----------------------------------EIGEHQGLMYHTLGQRKGLGIGGTK    252
At MnmA    ----------------------------------DFLGNHRGFWFYTIGQRQGLRLPGG-    259
Sc Mtu1    --------------------------------AKTTWGRHDGLWSYTIGQKVGISMPQAD    290
Pf MnmA    DITTTSSHLKKFRETFIPKMNLHYKNYLINLDDQTILDINSDIHLYAIGQHKNVTNYLHN    835
Bm MnmA    ----------------------------------QVIGQHTGLYNYTIGQRQGLGVSIDH    519
Ta MnmA    ----------------------------------KVIGEHNGLYNFTIGQKKTINNYLNP    564
Tg MnmA    ----------------------------------LALGSHDGLWNFTVGQRKGVTPCID-   1159
Et MnmA    ----------------------------------VSIGRHKGLWGFTVGQQKGVVPLLD-    728

Sy MnmA    -----------------------RPLYVVRID--------AAMNRVVVGEREEAT-----    288
Bs MnmA    ----------------------GEPWFAVGKD--------LEKNILYVDQGFHNPLL---    279
Ec MnmA    EG-------------------TEEPWYVVDKD--------VENNILVVAQGHEHPRL---    282
At MnmA    ------------------------PWYVVEKD--------TKNNVVFVSRNYYSID---K    284
Sc Mtu1    PN-------------------YQGTWFVSEKL--------RDTNEILIVRGRDNPAL---    320
Pf MnmA    LYNKKMININGYKKKHVKNVISSFQWIVVYKKIKRDMSTNLIHNFIYLTKNYDQDLFT--    893
Bm MnmA    GG-------------------EEGGWFVVSKD--------AASNTLYVTKEYNSRHFTGR    552
Ta MnmA    KH------------------VGNTPKYVVKKD--------IYKNTLYVSASYNEDFFTNP    598
Tg MnmA    ------------------------VARVRRLS--------SLPDSSL-TPHAASVDSEGS   1186
Et MnmA    ------------------------PRLCRRCH--------GASGG---------------    741

Sy MnmA    ------QAEAWVRQVNWVST-PAPDEP--LAVEVQVRY-------RTPAVPATL------    326
Bs MnmA    -----YSDKITATNISWVRSDIMKGEE--ISCTAKFRY-------RQEDHKVTV------    319
Ec MnmA    -----MSVGLIAQQLHWVDREPFTG-T--MRCTVKTRY-------RQTDIPCTV------    321
At MnmA    RRRIFRVGSL-----RWNSGKPSG---------------------KVRELRCKVRHGPGF    318
Sc Mtu1    -----YSDTMRIENFSSLGPREDTINAFQNTGALTLQF-------RSLQVPVQI------    362
Pf MnmA    ------------------------------------------------------------    893
Bm MnmA    SC-IR--RAFSLAHVRWNNPILLNSHG-----IN------EAD---YI------------    583
Ta MnmA    RG-IR--SSFKVRDFFWNTPDYKDLIN-----KV------QSEVSDNFKFKVKLRHTPNY    644
Tg MnmA    EEQTARAGDSRRRQARAPPGKPTKASA-----TGQGRQAADDEERRLLEAKCHR---DGD   1238
Et MnmA    ------------------------------------------------------------    741
```

```
Sy MnmA   IP--------ESPERVKLQFAEPQFGVTPGQAAVWYHGDL------------LLGGGILE    366
Bs MnmA   RM--------TGEGEAEVIFDEQVRAVTPGQAVVFYDGEE------------CLGGGTID    359
Ec MnmA   KA--------LDDDRIEVIFDEPVAAVTPGQSAVFYNGEV------------CLGGGIIE    361
At MnmA   YSCSFEMEG--DGDVAVVHLDEDDQGLAAGQFAAFYEGTT------------CIGSGV--    362
Sc Mtu1   KSCKLN--RSADNLDITIHLASKQRAITPGQSCCLYIDDR------------VLGSGPIS    408
Pf MnmA   ------------------------------------------------------------    893
Bm MnmA   --------------K-------------AEWEPPITRRDIKTMQDLTLDRNVASTE----    612
Ta MnmA   NYCNINFKGDEATDL-------------QKWLPTTYELAAATIASV--DNSKCSIKMI--    687
Tg MnmA   DSCLRSANHTVRGRLECT----DNADRPDGRVPVLSEGNEAPS------SSSCSSSAEAD   1288
Et MnmA   ------------------------------------------------------------    741

Sy MnmA   RPSP--------------------------------------------------------    370
Bs MnmA   DVYKDGTK----------------------------------------LWYV--------    371
Ec MnmA   QRLPLPV-----------------------------------------------------    368
At MnmA   --------ILESWDDQCFPVCAKALQLAALED-----KTKLG------KPVKIMTMPTMS    403
Sc Mtu1   HVNNNDTH----------------------------------------A-----------    417
Pf MnmA   ------------------------------------------------------------    893
Bm MnmA   --------------KLFL---KV-------RNGTVL-------------YEAELKL----    631
Ta MnmA   --------------QLILTFCTI-------EDVGFI-------------HTADSKATTIA    713
Tg MnmA   EGQEEGGDRQSRLSSSSFAACLSGSPQNLFEPANPFSRTGLGAASLAGRWVVAAKHPPSN   1348
Et MnmA   ------------------------------------------PPKLSGPWSVVGKLPEAN    759

Sy MnmA   ------------------------------------------------------------    370
Bs MnmA   ------------------------------------------------------------    371
Ec MnmA   ------------------------------------------------------------    368
At MnmA   ------SVEADTGE--ASAE----------QKLVNA------------------------    421
Sc Mtu1   ------------------------------------------------------------    417
Pf MnmA   ---------------------------------------------------------HIR    896
Bm MnmA   -----SPPIRKGQGVTQHGWAVL----------DRSDPGLAT----GQYAA--LYRGNIC    670
Ta MnmA   LFFIFGSNKDEKDGTSIELEDIV----------HEPAPSEHG----SKKSP--SELHIYE    757
Tg MnmA   ALFVVSEKEMKAAA--AVAES-----VGYSLDVLAAGPGVRT-LGDSQTYLLALLLTLQQ   1398
Et MnmA   ALFVVSKEEEAAAE--DRLRALATEPGNYSEPLGA--PLLRAAANGDKAAALALKLRQLR    815

Sy MnmA   ------------------------------------------------------------    370
Bs MnmA   ------------------------------------------------------------    371
Ec MnmA   ------------------------------------------------------------    368
At MnmA   ------------------------------------------------------------    421
Sc Mtu1   ------------------------------------------------------------    417
Pf MnmA   TKCKLHNIKWIEGKLPACIKKQFKKYN-----------------KINKKKKKINNNNNKY    939
Bm MnmA   IGSGIIYQSF--------------------------------------------------    680
Ta MnmA   IGKPIDYLDWKQHKRTNSSQSGRNGTHGKTHTEKRRNSDPTKRSKVHKPQRITSELEQKT    817
Tg MnmA   KFLRVDNIQWISHPPCAACNAEEQ-------PVS---RDPSFLGTLLGSRDPRARLRGDE   1450
Et MnmA   TCLRVDNIRWFHGKAPAGFDASATL-----QPGCLREEGPTL-AAVAASAVAAAKT-NTA    868

Sy MnmA   ------------------------------------------------------------    370
Bs MnmA   ------------------------------------------------------------    371
Ec MnmA   ------------------------------------------------------------    368
At MnmA   ------------------------------------------------------------    421
Sc Mtu1   ------------------------------------------------------------    417
Pf MnmA   KTN-----ETFHVYNNIQESGKKKKKKKVK--N-------IPH-----------------    968
Bm MnmA   ------------------------------------------------------------    680
Ta MnmA   KPNS-PQQTTTKQQTNTKEQNTNKQQSSVEQKTKADQSGPITRPFI----------SPSV    866
Tg MnmA   KSLETADEFAFLRWALEGSRDKRPRLYDVQVRHAAGTACAAIHRRVRLCLFPPERRSPSF   1510
Et MnmA   LPMERPA-ESFL----TG-SSKGGAGFVVQVRHSAGFHGVAKHNFKVLCVSRGH------    916
```

```
Sy MnmA    ------------------------------------------------------------   370
Bs MnmA    ------------------------------------------------------------   371
Ec MnmA    ------------------------------------------------------------   368
At MnmA    ------------------------------------------------------------   421
Sc Mtu1    ------------------------------------------------------------   417
Pf MnmA    ------------------------------------------DEKTIFVKIRNS------   980
Bm MnmA    ------------------------------------------------------------   680
Ta MnmA    LKPSEKAERVFSSNEKPAVQQRLFKPRENKYQKRTERYDTDMDFEKIFNPVHENAKDDEP   926
Tg MnmA    LSPSAVSEESG-----------------------GRRK----ESRAPFGPSRVETSAGSS  1543
Et MnmA    -------------------------------------------------------SGNSS   921

Sy MnmA    ------------------------------------------------------------   370
Bs MnmA    ------------------------------------------------------------   371
Ec MnmA    ------------------------------------------------------------   368
At MnmA    ------------------------------------------------------------   421
Sc Mtu1    ------------------------------------------------------------   417
Pf MnmA    EQIKKAKIKFSLSNNTAYLKVKQKDTGFSPGQIITLYFPFIIKKNNKVTYITNLNKYNNL  1040
Bm MnmA    ------------------------------------------------------------   680
Ta MnmA    GSYMPFESIYDLSLDKCRLNKAFPPKGKPAG------HKFE-------------------   961
Tg MnmA    GAWTAW------------IELAEPDEGLAPGQIAAIYEGEE-------------------  1572
Et MnmA    PIEEAE------------LLLEEPDVGLAPGQVAAFYRDDE-------------------   950

Sy MnmA    ------------------------------------------------------------   370
Bs MnmA    ------------------------------------------------------------   371
Ec MnmA    ------------------------------------------------------------   368
At MnmA    ------------------------------------------------------------   421
Sc Mtu1    ------------------------------------------------------------   417
Pf MnmA    INTNKNTIYYHCLGSATISNQFLD-------YNLYQHIKNIHQINDLNMS-K--------  1084
Bm MnmA    ------------------------------------------------------------   680
Ta MnmA    -----------CIDLDVIRVSKDFKNISLVNANVIHPKSNTHKICKISFDNKVLVDKVDD  1010
Tg MnmA    -----------CLGAGRISARQGQ------------------------------------  1585
Et MnmA    -----------CIGSGRISALQGS------------------------------------   963

Sy MnmA    ------------------------------------------------------------   370
Bs MnmA    ------------------------------------------------------------   371
Ec MnmA    ------------------------------------------------------------   368
At MnmA    ------------------------------------------------------------   421
Sc Mtu1    ------------------------------------------------------------   417
Pf MnmA    ------------------------------------------------------------  1084
Bm MnmA    ------------------------------------------------------------   680
Ta MnmA    SMEFTAVVLEHVRSISILSVISTNPNMERSAIQFVITGKDKINVMNDYVSVMSTILKSEL  1070
Tg MnmA    ------------------------------------------------MAVEAALRSAGL  1597
Et MnmA    ------------------------------------------------FALKSIIDRLG-   974

Sy MnmA    -      370
Bs Mnm     -      371
Ec MnmA    -      368
At MnmA    -      421
Sc Mtu1    -      417
Pf MnmA    -     1084
Bm MnmA    -      680
Ta MnmA    -     1070
Tg MnmA    N     1598
Et MnmA    -      974
```

Figure 3- figure supplement 3- source data 2. Multiple sequence alignment used for **Figure 3- figure supplement 3(E)**.
