# Supplementary material for: The Plasmodium falciparum apicoplast cysteine desulfurase provides sulfur for both iron-sulfur cluster assembly and tRNA modification
Source: eLife. 2023 May 11;12:e84491. doi: 10.7554/eLife.84491 (PMC10219651; doi:10.7554/eLife.84491)
Supplement: Figure 3—figure supplement 4—source data 2. — The red boxes correspond to the cropped images in Figure 3—figure supplement 4C. [file elife-84491-fig3-figsupp4-data2.zip › Figure 3- figure supplement 4- source data 1/Figure 3- figure supplement 4- source data 1.pdf]

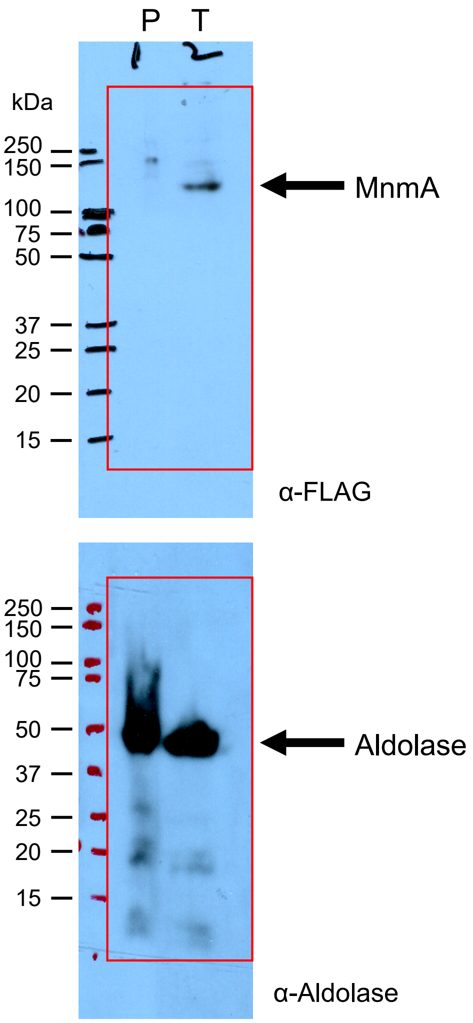

Figure 3- figure supplement 4- source data 1. Full immunoblot shown in **Figure 3- figure supplement 4(C)**. The red boxes correspond to the cropped images in **Figure 3- figure supplement 4(C)**.
