# Supplementary material for: The Plasmodium falciparum apicoplast cysteine desulfurase provides sulfur for both iron-sulfur cluster assembly and tRNA modification
Source: eLife. 2023 May 11;12:e84491. doi: 10.7554/eLife.84491 (PMC10219651; doi:10.7554/eLife.84491)
Supplement: Figure 3—figure supplement 8—source data 3. [file elife-84491-fig3-figsupp8-data3.pdf]

```
Ec SufE   ------------------------M-----------------------------------   1
Pf SufE   ----------------------DEY-----------------------------------   103
Pf MnmA   --------------INDVQKIYDEVNNIKDKEQKINYLMEQ-----C-------SSLCK-   178
Bm MnmA   ------------------------------------------------------------   84
Ta MnmA   ---------------------------LEDNLNKSDFVQELARL--GRSIY---SNIPTP   144
Tg MnmA   LPPRMRELLSELRRQKDLHAVFEKLVSFASSVP--LYPARDASAVSSRASTASPPSPPRP   394
Et MnmA   ------------RRWLTLCRVFRGAPTAESRI---KILMHLAF-----------------   172

Ec SufE   -----------------------ALLPDKEKLLRNFL-------RCANWEEKYLYIIELG   31
Pf SufE   -----------------------NLTPKLKKTVELFQS---MP-NSPYY--KSQQVILMG   134
Pf MnmA   ----------------------ENYFPPILNLNKAYRN---KRIDEFNKGNKNFYINEVG   213
Bm MnmA   -------------------------------------------------KDEHFHLFNLS   95
Ta MnmA   ----TL----------------FKSVPNGINLLKSFNS---NDFNSLGCNGRDYFLVKSP   181
Tg MnmA   VPSASLSPAPPPKATETSGRADDRLLPPAQLLQQPRRE--------------------AS   434
Et MnmA   --------------QETNG-SDFLSFTGKRPLTRIKQSAHGPSASSEALESGSAAGVDEA   217

                              *
Ec SufE   QRLPELRD-EDRSPQNSIQGCQSQVWIVMRQN----AQGIIELQGDSDAA-IVKGLIAVV   85
Pf SufE   KKISSMPD-KHKIRQNQVLGCQSVVYIYPKVEENEDKKKVIVWLGHSDGL-LTKGIVYIL   192
Pf MnmA   KN----------IWYKYVNRCDEILFMAIDIQIDEDEQRNNS---IKDVHDVHDDNIKTC   260
Bm MnmA   QFSPEFADQFGAKPYAKLQDCITPIYLAAI------------------------------   125
Ta MnmA   VP----------VDYKLLDDCSSSIYISIHLDKDKKIYLDG----ISDSFVF-KGII---   223
Tg MnmA   EEADEEGD--SPEAWERVAGCAALVRIRVCLRRVLSPKREG--------------ESRAC   478
Et MnmA   SVCPSTSDLVLSDIWRPVTGCAAVAAVKVSLHYPVRPQQRSLAASI-------TSNSRLC   270

Ec SufE   -----FILYDQM------------------TPQDIVNFDVRPWFE-KMALTQHLTPSRSQ   121
Pf SufE   -----TDGLSGY------------------MPEDILKVN--PNFITLTGISEFLTMSRIN   227
Pf MnmA   TLIKDDKHFEKY------------------K--DIHNDNILKNIL---PLD-----KKID   292
Bm MnmA   ------------------------------------------------------------   125
Ta MnmA   SLLISLIGWKNS------------------T--EVE------------------------   239
Tg MnmA   SVFREADVREEPQQGALASLSVAASSSSGASCHDVEGAKKER---------RELRREDRD   529
Et MnmA   CVA---------------------------------------------------------   273

Ec SufE   GLEAMIRA---IRAKAA-----------------ALS-----------------------   138
Pf SufE   GYLNIMNK---IKIFCT-----------------NILKNMDN------------------   249
Pf MnmA   SIKNMLNHKYMKKKKCIITIDAYSNNLILYCFLYLILKHINKMYLYSFMNIQIKE---IT   349
Bm MnmA   --------RYWHEGVERLAIDGTSNSLVGKTYLALMLSALHGNPLDELASIFESK-----   172
Ta MnmA   -VDNVKKHKFFKKVIDH--------GIISENSLNSILNHIKN------------------   272
Tg MnmA   GEDE--RR----RWELRVDLRGWSDSLVVRAWLAILVVGLNNAAPDTVLALSTDD---IL   580
Et MnmA   -----------AKQGAKVHVHGESDSLVVGGLLQLLSRSLSGSAPCGVFALSALGSTKLL   322

Ec SufE   ------------------------------------------------------------   138
Pf SufE   ------------------------------------------------------------   249
Pf MnmA   AKLKE-------------------------------------------------------   354
Bm MnmA   -------------GRTFEQLSRFTDGISQRICQELFRLIGFNTN-----KAAGYDDSE--   212
Ta MnmA   ------------------------------------------------------------   272
Tg MnmA   REAGLMPSSTPSGGKGHKDTVKETQGEPEKQTE-----EGEDGH-----REAEEEAEEAE   630
Et MnmA   QFSSLLNLLPDSRVRGFRTAMHILMGSTQDLLQRYFCNEVLPPPAELPQNAHGEQGT-AP   381

Ec SufE   ------------------------------------------------------------   138
Pf SufE   ------------------------------------------------------------   249
Pf MnmA   ----------------------------------------------------LFDLHFNV   362
Bm MnmA   --ADMD-----KNDKFQFKRLVPKPAL-------RN------------------------   234
Ta MnmA   ------------------------------------------------------------   272
Tg MnmA   EEAEEEEEEEEEKERKGSKRESEEERLQEGEERLREEETRKCREEEKRRLVVPQGLEFML   690
Et MnmA   NKQDITTGRTQAKVPKGTSLRPPRPAN-DALSGYRQEPHGD-----QWGNPIPQH---AL   432
```

Ec **SufE**	------------------	138
Pf **SufE**	------------------	249
Pf **MnmA**	HHIIDYIHEYIYNFLMSY	380
Bm **MnmA**	------------------	234
Ta **MnmA**	------------------	272
Tg **MnmA**	RSIQRQVREQLSRLAEEE	708
Et **MnmA**	PSIPRS--KEIAKL----	444

Figure 3- figure supplement 8- source data 3. Multiple sequence alignment used for **Figure 3- figure supplement 8(H)**.
