# Supplementary material for: The Plasmodium falciparum apicoplast cysteine desulfurase provides sulfur for both iron-sulfur cluster assembly and tRNA modification
Source: eLife. 2023 May 11;12:e84491. doi: 10.7554/eLife.84491 (PMC10219651; doi:10.7554/eLife.84491)
Supplement: Figure 4—source data 1. [file elife-84491-fig4-data1.zip › Figure 4- source data 1/Figure 4- source data 1.pptx]

## Slide 1
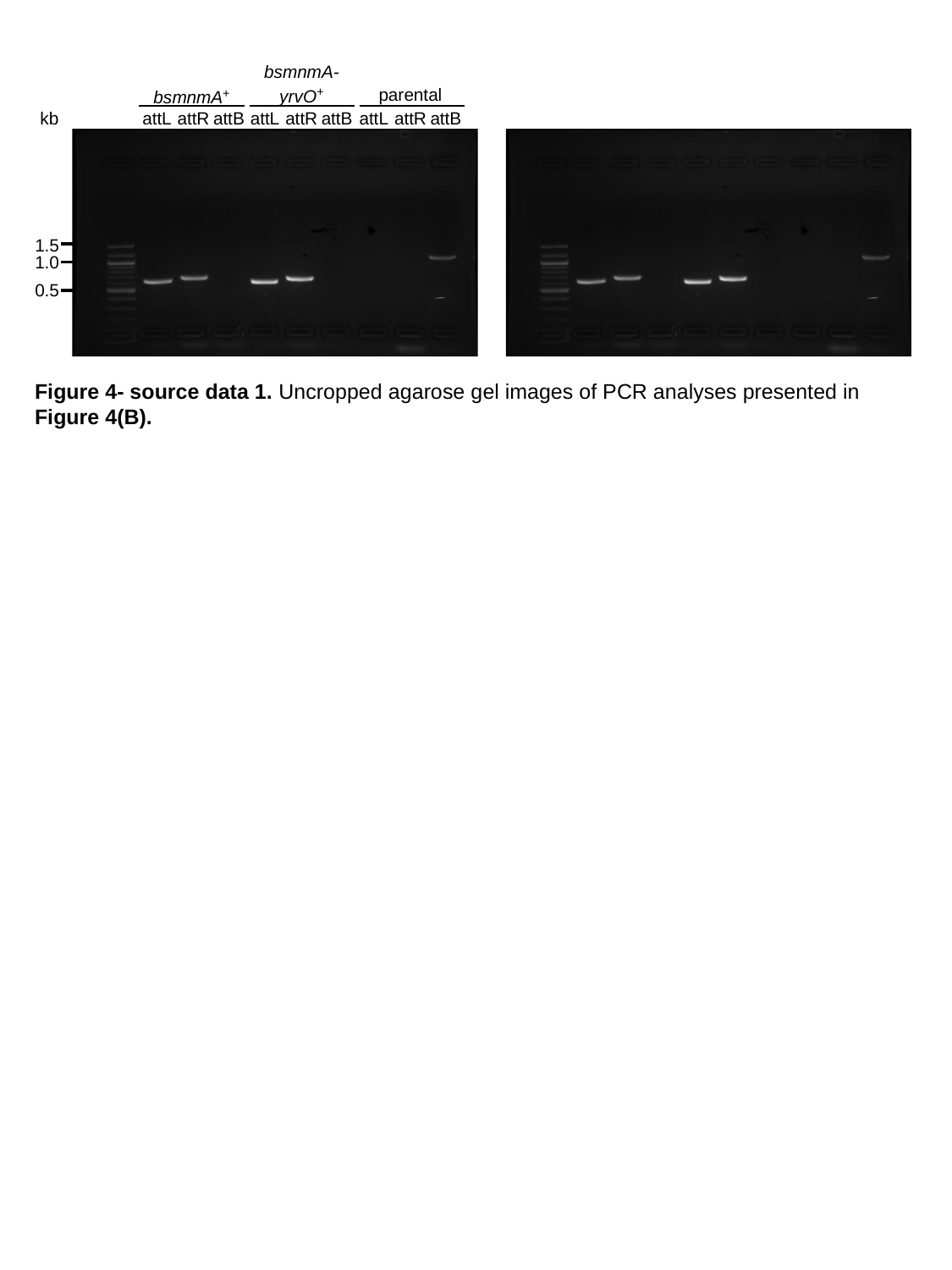

bsmnmA-yrvO+
parental
bsmnmA+
kb
attL
attR
attB
attL
attR
attB
attL
attR
attB
1.5
1.0
0.5
Figure 4- source data 1. Uncropped agarose gel images of PCR analyses presented in Figure 4(B).
