# Supplementary material for: The Plasmodium falciparum apicoplast cysteine desulfurase provides sulfur for both iron-sulfur cluster assembly and tRNA modification
Source: eLife. 2023 May 11;12:e84491. doi: 10.7554/eLife.84491 (PMC10219651; doi:10.7554/eLife.84491)
Supplement: Figure 5—source data 1. [file elife-84491-fig5-data1.zip › Figure 5- source data 1/Figure 5- source data 1.pdf]

Figure 5(A)

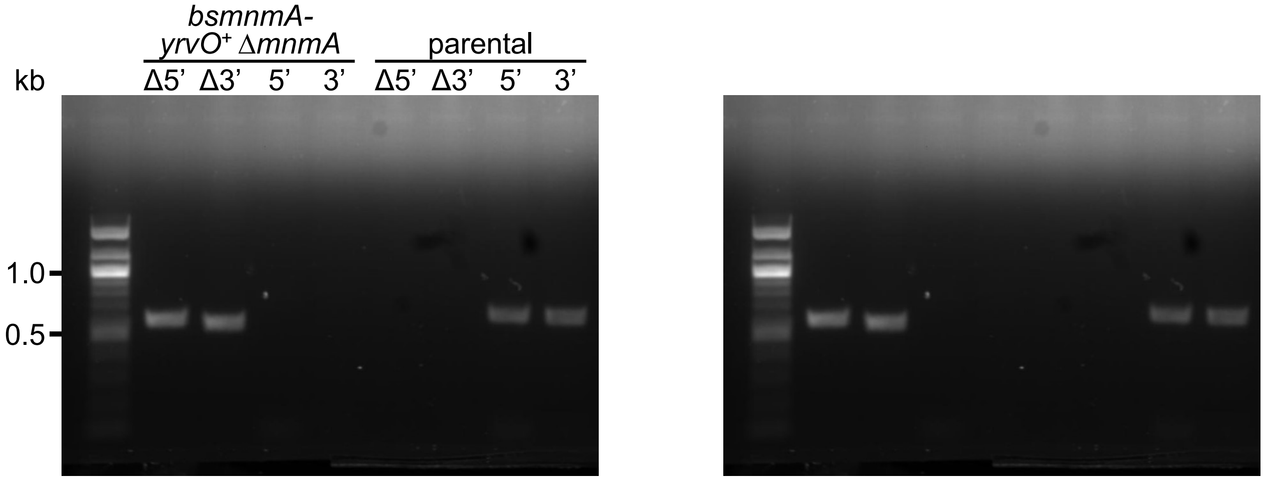

Figure 5(C)

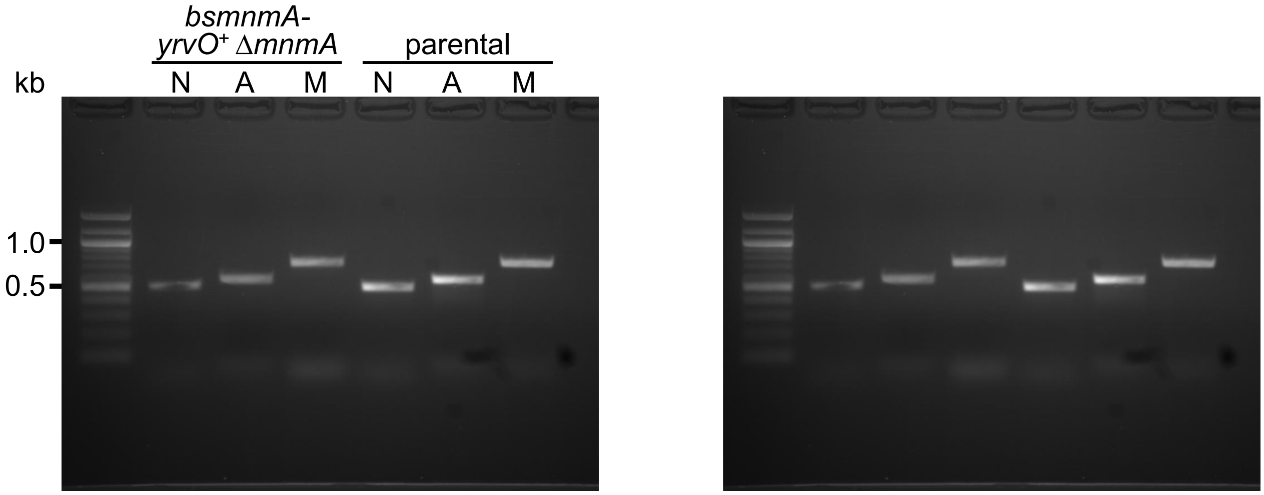

Figure 5- source data 1. Uncropped agarose gel images of PCR analyses presented in **Figures 5(A)** and **5(C).**
