# Supplementary material for: The Plasmodium falciparum apicoplast cysteine desulfurase provides sulfur for both iron-sulfur cluster assembly and tRNA modification
Source: eLife. 2023 May 11;12:e84491. doi: 10.7554/eLife.84491 (PMC10219651; doi:10.7554/eLife.84491)
Supplement: Figure 5—source data 1. [file elife-84491-fig5-data1.zip › Figure 5- source data 1/Figure 5- source data 1.pptx]

## Slide 1
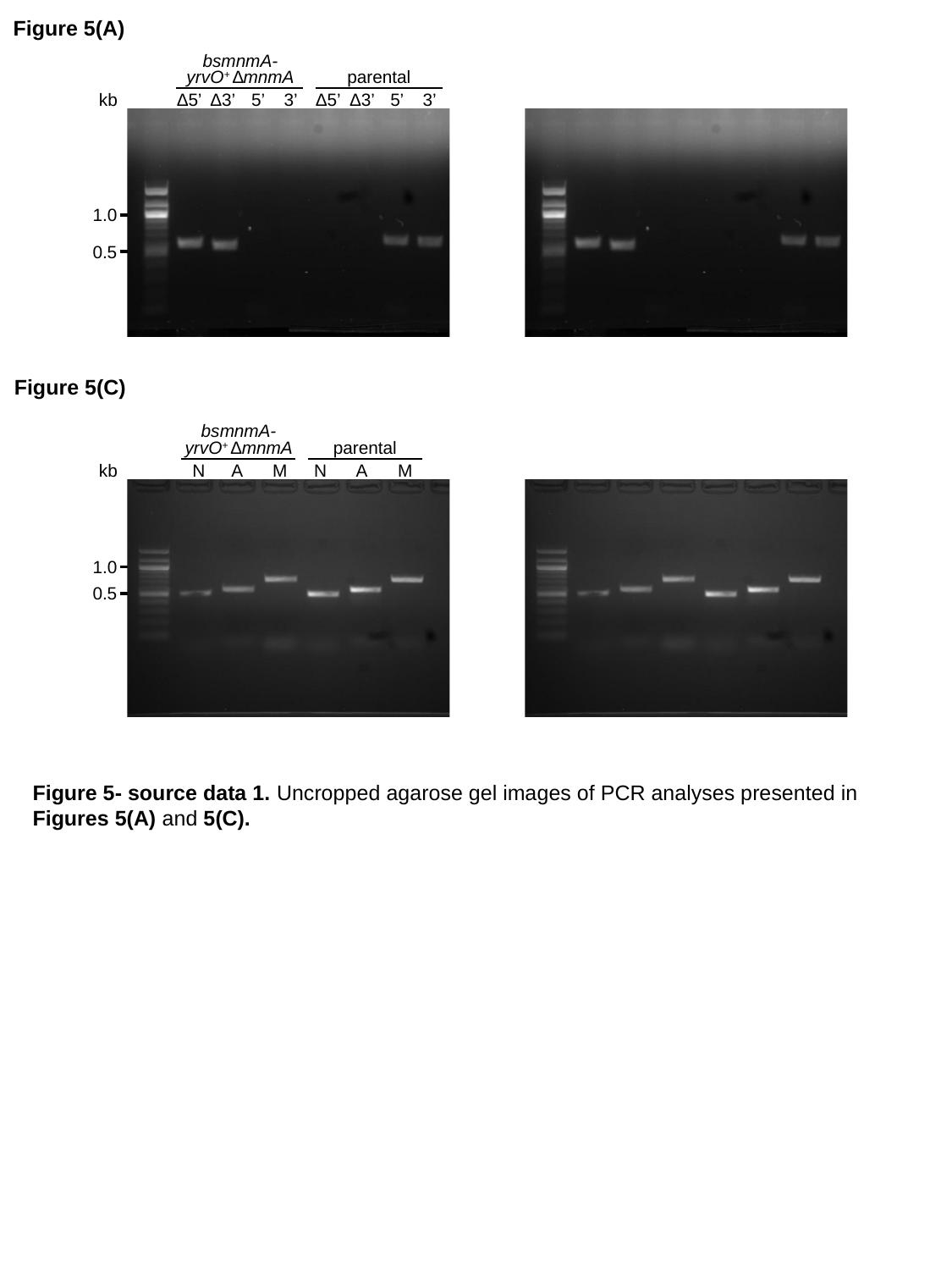

Figure 5(A)
bsmnmA-
yrvO+ ∆mnmA
parental
kb
Δ5’
Δ3’
5’
3’
Δ5’
Δ3’
5’
3’
1.0
0.5
Figure 5(C)
bsmnmA-
yrvO+ ∆mnmA
parental
kb
N
A
M
N
A
M
1.0
0.5
Figure 5- source data 1. Uncropped agarose gel images of PCR analyses presented in Figures 5(A) and 5(C).
