# Supplementary material for: The Plasmodium falciparum apicoplast cysteine desulfurase provides sulfur for both iron-sulfur cluster assembly and tRNA modification
Source: eLife. 2023 May 11;12:e84491. doi: 10.7554/eLife.84491 (PMC10219651; doi:10.7554/eLife.84491)
Supplement: Figure 6—source data 1. [file elife-84491-fig6-data1.zip › Figure 6- source data 1/Figure 6- source data 1.pdf]

Figure 6(A)

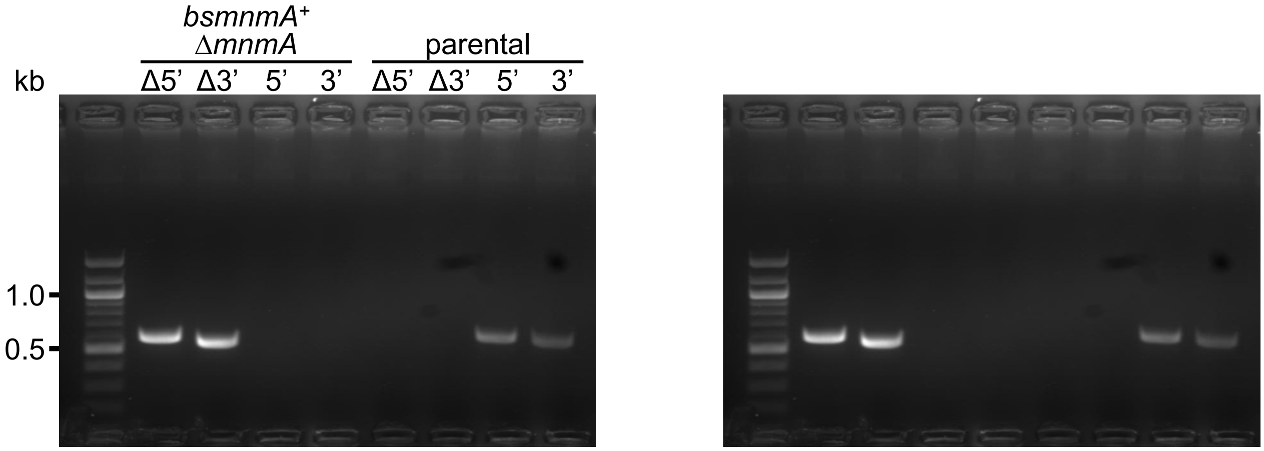

Figure 6(C)

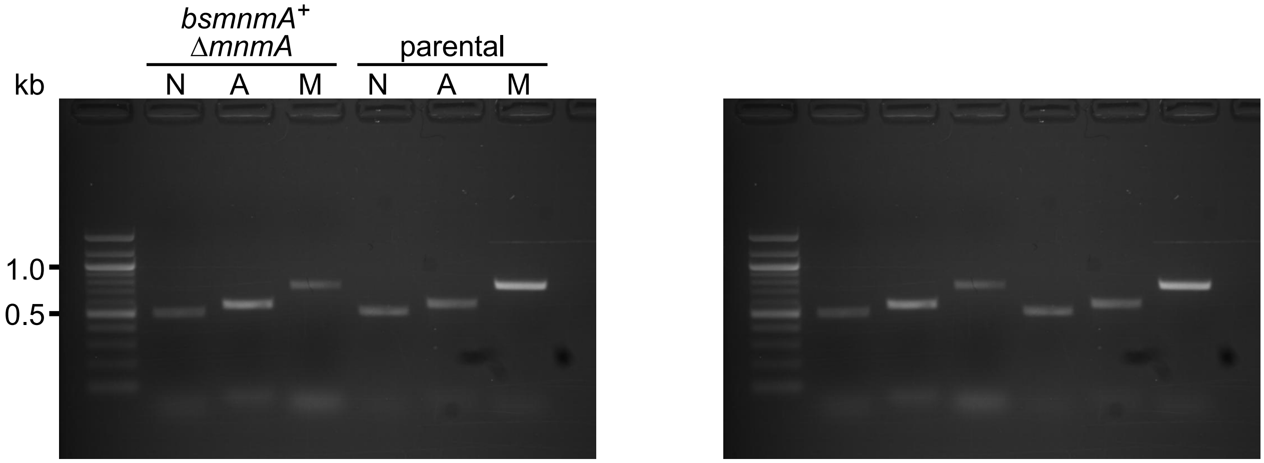

Figure 6- source data 1. Uncropped agarose gel images of PCR analyses presented in **Figures 6(A)** and **6(C)**.
