# Supplementary material for: The Plasmodium falciparum apicoplast cysteine desulfurase provides sulfur for both iron-sulfur cluster assembly and tRNA modification
Source: eLife. 2023 May 11;12:e84491. doi: 10.7554/eLife.84491 (PMC10219651; doi:10.7554/eLife.84491)
Supplement: Figure 6—source data 1. [file elife-84491-fig6-data1.zip › Figure 6- source data 1/Figure 6- source data 1.pptx]

## Slide 1
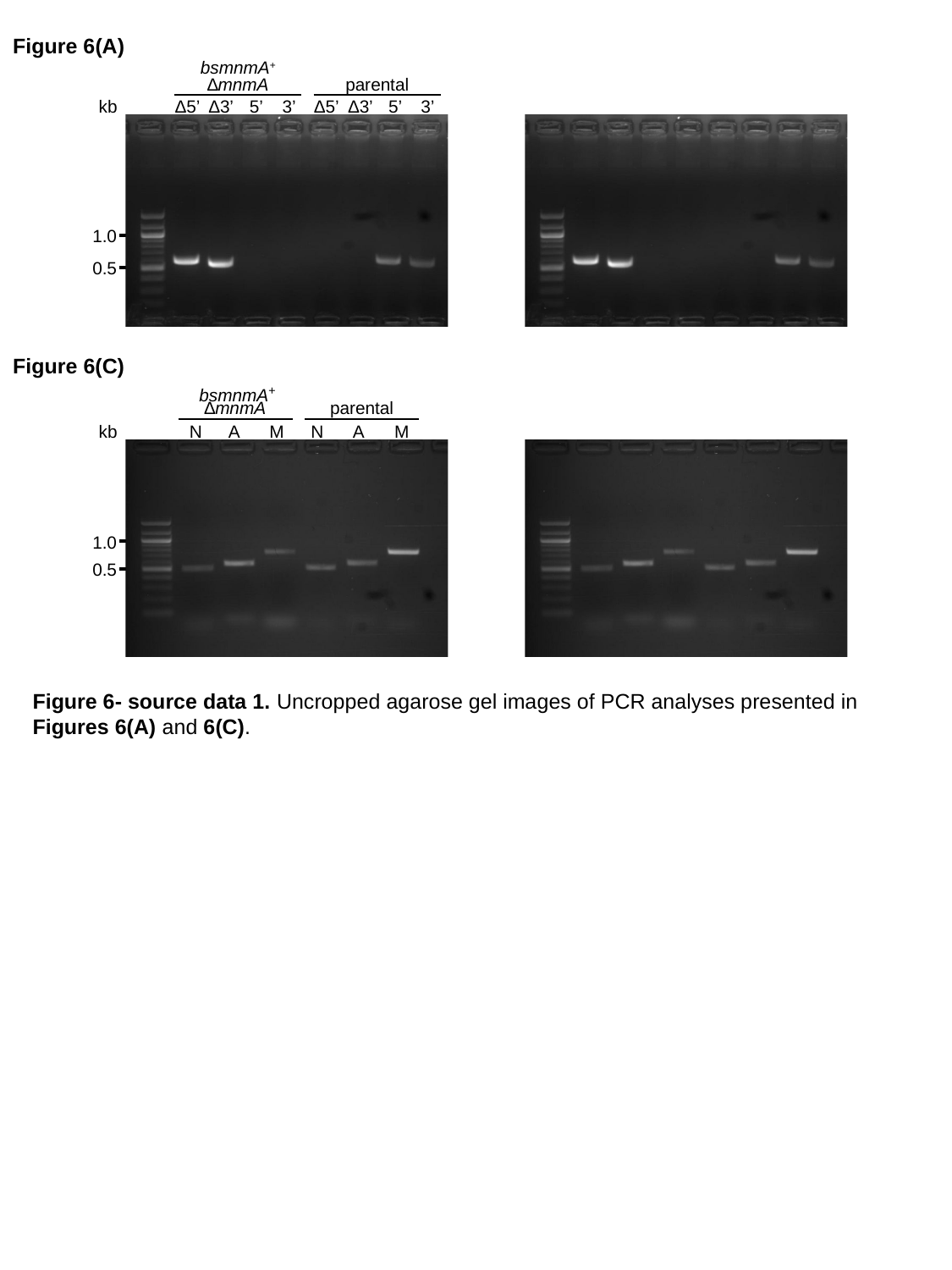

Figure 6(A)
bsmnmA+
∆mnmA
parental
kb
Δ5’
Δ3’
5’
3’
Δ5’
Δ3’
5’
3’
1.0
0.5
Figure 6(C)
bsmnmA+
∆mnmA
parental
kb
N
A
M
N
A
M
1.0
0.5
Figure 6- source data 1. Uncropped agarose gel images of PCR analyses presented in Figures 6(A) and 6(C).
