# Supplementary material for: The Plasmodium falciparum apicoplast cysteine desulfurase provides sulfur for both iron-sulfur cluster assembly and tRNA modification
Source: eLife. 2023 May 11;12:e84491. doi: 10.7554/eLife.84491 (PMC10219651; doi:10.7554/eLife.84491)
Supplement: Figure 7—figure supplement 1—source data 1. — (A) Multiple sequence alignment used for Figure 7—figure supplement 1. (B) Table showing the percentage sequence identity among cysteine desulfurase orthologs based on the sequence alignment shown in panel (A). [file elife-84491-fig7-figsupp1-data1.pdf]

A

```
Pf SufS    --------------------MLRGPRCLYI-------YLFFV---FLPFSFCYIRNNDNR    30
Bs SufS    ------------------------------------------------------------     0
Ec SufS    ------------------------------------------------------------     0
At cNif1   ---------------------MEGVAMK------------LP---SFPNA---ISIGHRS    21
Pf IscS   MKFLQIIKHL-KL-QNKKNALDNFVNCRTYEHISNINKLFLNNFSSTKEHSEHGQVKHEN    58
Bs YrvO    ------------------------------------------------------------     0
Ec IscS    ------------------------------------------------------------     0
At mNif1   MASKVIS---ATIRRTLTKPHGTFSRCR-----------YL----STA---AA-------    32
Sc Nfs1    MLKSTATRSITRLSQVYNVPAATYRACLVSRR-------FY----SPP---AAGVKLDDN    46

Pf SufS    FVY--------------------------------IVKSIRKGPNIK-------LR----    47
Bs SufS    ------------------------------------------------------------     0
Ec SufS    ------------------------------------------------------------     0
At cNif1   FSR------------------------------------VRCSSSLSV----CSAA----    37
Pf IscS    FLNSTLKYEENSQNGSTNNLKNGKYNMYVSEGNVNINEEKYKDNNISSNNTQYNNNSSNS   118
Bs YrvO    ------------------------------------------------------------     0
Ec IscS    ------------------------------------------------------------     0
At mNif1   ------------------------------------------------------------    32
Sc Nfs1    FS---------------------------LETHTDIQA---------AAKAQASARASAS    70

Pf SufS    -LTKDEKPNIDNHIIDYFKNVREHFPFFKE---NKSLIYFDSAATTHKPSCVIEKMSEFY   103
Bs SufS    ---------------MNITDIREQFPILHQQVNGHDLVYLDSAATSQKPRAVIETLDKYY    45
Ec SufS    -------------MIFSVDKVRADFPVLSREVNGLPLAYLDSAASAQKPSQVIDAEAEFY    47
At cNif1   -AASSATISTDSESVSLGHRVRKDFRILHQEVNGSKLVYLDSAATSQKPAAVLDALQNYY    96
Pf IscS    GSLNDEGPL-WKEHID--------DVVNENKKKKMNRFYLDSQATTMIDPRVLDKMLPYM   169
Bs YrvO    ----------------------------------MERIYLDHAATSPMDERVLEQMIPHF    26
Ec IscS    ---------------------------------MKLPIYLDYSATTPVDPRVAEKMMQFM    27
At mNif1   ---ATEV----NYEDE--------SIMMKGVRISGRPLYLDMQATTPIDPRVFDAMNASQ    77
Sc Nfs1    GTTPDAVVASGSTAMS--------HAYQENTGFGTRPIYLDMQATTPTDPRVLDTMLKFY   122

Pf SufS    --KKENSNIHRGIYKLSHNATNNYEKVRETIKEYINCEKNDNIIFTNGSTYGLNVVCKMM   161
Bs SufS    --NQYNSNVHRGVHTLGTRATDGYEGAREKVRKFINAKSMAEIIFTKGTTTSLNMVALSY   103
Ec SufS    --RHGYAAVHRGIHTLSAQATEKMENVRKRASLFINARSAEELVFVRGTTEGINLVANSW   105
At cNif1   --EFYNSNVHRGIHYLSAKATDEFELARKKVARFINASDSREIVFTRNATEAINLVAYSW   154
Pf IscS    --TYIYGNAHSRNHFFGWESEKAVEDARTNLLNLINGKNNKEIIFTSGATESNNLALIGI   227
Bs YrvO    --SGSFGNPSS-IHSFGRESRKWVDEARAQIAAEIG-AAEQEIIFTSGGTEADNLAIMGT    82
Ec IscS    TMDGTFGNPASRSHRFGWQAEEAVDIARNQIADLVG-ADPREIVFTSGATESDNLAIKGA    86
At mNif1   --IHEYGNPHSRTHLYGWEAENAVENARNQVAKLIE-ASPKEIVFVSGATEANNMAVKGV   134
Sc Nfs1    --TGLYGNPHSNTHSYGWETNTAVENARAHVAKMIN-ADPKEIIFTSGATESNNMVLKGV   179

                               *
Pf SufS    IEEI--IKKEEDEIYLSYMEHHSNIIPWQEYINKEKKGRIKYVPLNKSGYINIKKLISNM   219
Bs SufS    ARAN---LKPGDEVVITYMEHHANIIPWQQAVKA-TGATLKYIPLQEDGTISLEDVRETV   159
Ec SufS    GNSN---VRAGDNIIISQMEHHANIVPWQMLCAR-VGAELRVIPLNPDGTLQLETLPTLF   161
At cNif1   GLSN---LKPGDEVILTVAEHHSCIVPWQIVSQK-TGAVLKFVTLNEDEVPDINKLRELI   210
Pf IscS    CTYYNKLNKQKNHIITSQIEHKCILQTCRFLQ-T-KGFEVTYLKPDTNGLVKLDDIKNSI   285
Bs YrvO    ALARKD---LGRHIITTKIEHHAVLHTCEKLE-G-DGFDITYLDVDQNGRVSAKQVKEAL   137
Ec IscS    ANFYQK---KGKHIITSKTEHKAVLDTCRQLE-R-EGFEVTYLAPQRNGIIDLKELEAAM   141
At mNif1   MHFYKD---TKKHVITTQTEHKCVLDSCRHLQ-Q-EGFEVTYLPVKTDGLVDLEMLREAI   189
Sc Nfs1    PRFYKK---TKKHIITTRTEHKCVLEAARAMM-K-EGFEVTFLNVDDQGLIDLKELEDAI   234

Pf SufS    NINTKVISICHASNVIGNIQNIEKIIKKIKNVYPHIIIIIDASQSFAHIKYDIKKMKKNK   279
Bs SufS    TSNTKIVAVSHVSNVLGTVNPIKEMAKIAHDNG--AVIVVDGAQSTPHMKIDVQDLD---   214
Ec SufS    DEKTRLLAITHVSNVLGTENPLAEMITLAHQHG--AKVLVDGAQAVMHHPVDVQALD---   216
At cNif1   SPKTKLVAVHHVSNVLASSLPIEEIVVWAHDVG--AKVLVDACQSVPHMVVDVQKLN---   265
Pf IscS    KDNTIMASFIFVNNEIGVIQDIENIGNLCKEKN--ILFHTDASQAAGKVPIDVQKMN---   340
Bs YrvO    RDDTILVTVMYGNNEVGTVQPIEEIGELLKEHK--AYFHTDAVQAFGLLPIDVKNSH---   192
Ec IscS    RDDTILVSIMHVNNEIGVVQDIAAIGEMCRARG--IIYHVDATQSVGKLPIDLSQLK---   196
At mNif1   RPDTGLVSIMAVNNEIGVVQPMEEIGMICKEHN--VPFHTDAAQAIGKIPVDVKKWN---   244
Sc Nfs1    RPDTCLVSVMAVNNEIGVIQPIKEIGAICRKNK--IYFHTDAAQAYGKIHIDVNEMN---   289
```

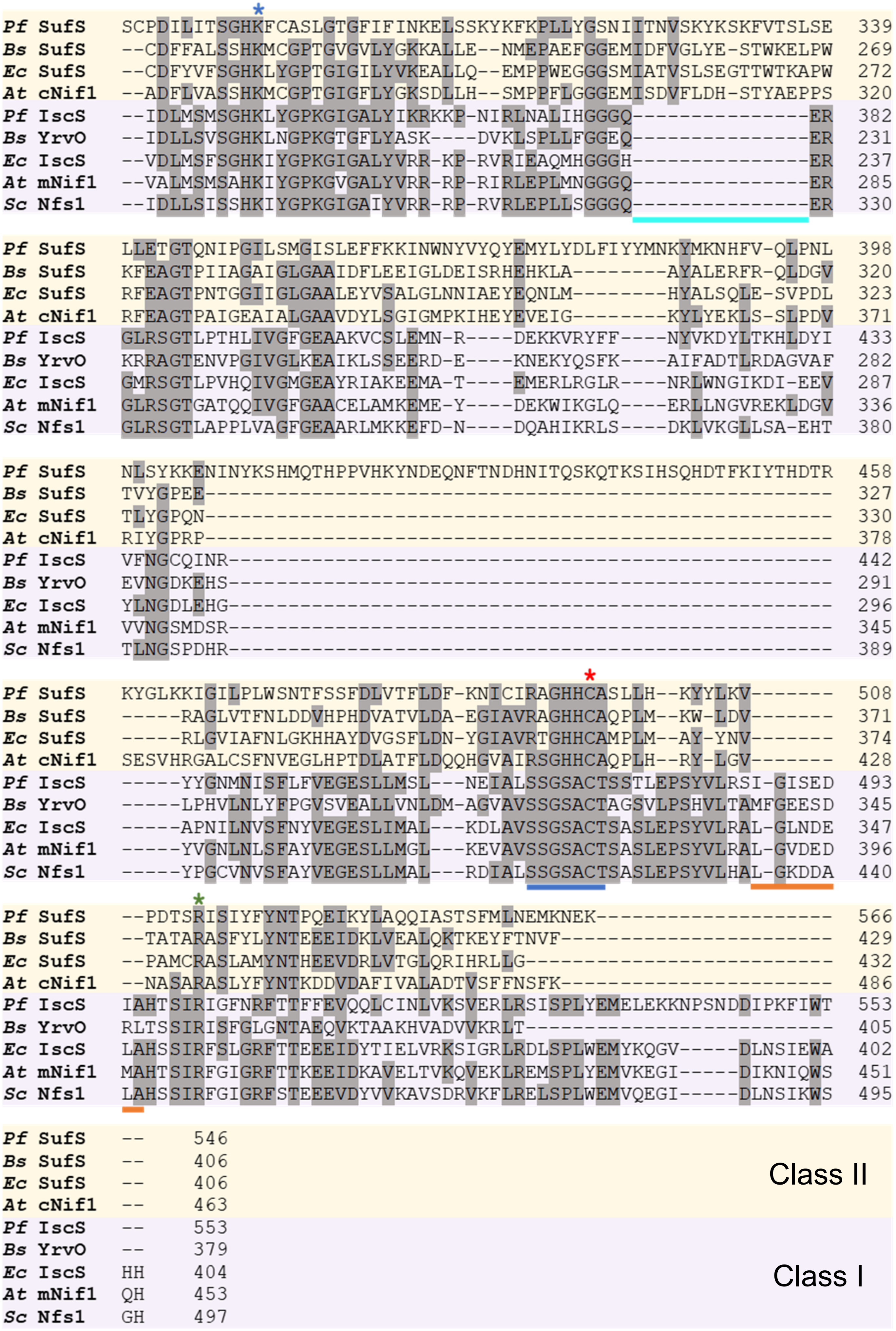

Pf SufS SCPDILITSGHKFCASLGTGFIFINKELSSKYKFKPLLYGSNIITNVSKYKSKFVTSLSE 339
Bs SufS --CDFFALSSHKMCGPTGVGVLYGKKALLE--NMEPAEFGGEMIDFVGLYE-STWKELPW 269
Ec SufS --CDFYVFSGHKLYGPTGIGILYVKEALLQ--EMPPWEGGGSMIATVSLSEGTTWTKAPW 272
At cNif1 --ADFLVASSHKMCGPTGIGFLYGKSDLLH--SMPPFLGGGEMISDVFLDH-STYAEPPS 320
Pf IscS --IDLMSMSGHKLYGPKGIGALYIKRKKP-NIRLNALIHGGGQ---------------ER 382
Bs YrvO --IDLLSVSGHKLNGPKGTGFLYASK----DVKLSPLLFGGEQ---------------ER 231
Ec IscS --VDLMSFSGHKIYGPKGIGALYVRR-KP-RVRIEAQMHGGGH---------------ER 237
At mNif1 --VALMSMSAHKIYGPKGVGALYVRR-RP-RIRLEPLMNGGGQ---------------ER 285
Sc Nfs1 --IDLLSISSHKIYGPKGIGAIYVRR-RP-RVRLEPLLSGGGQ---------------ER 330
Pf SufS LLETGTQNIPGILSMGISLEFFKKINWNYVYQYEMYLYDLFIYYMNKYMKNHFV-QLPNL 398
Bs SufS KFEAGTPIIAGAIGLGAAIDFLEEIGLDEISRHEHKLA--------AYALERFR-QLDGV 320
Ec SufS RFEAGTPNTGGIIGLGAALEYVSALGLNNIAEYEQNLM--------HYALSQLE-SVPDL 323
At cNif1 RFEAGTPAIGEAIALGAAVDYLSGIGMPKIHEYEVEIG--------KYLYEKLS-SLPDV 371
Pf IscS GLRSGTLPTHLIVGFGEAAKVCSLEMN-R----DEKKVRYFF----NYVKDYLTKHLDYI 433
Bs YrvO KRRAGTENVPGIVGLKEAIKLSSEERD-E----KNEKYQSFK----AIFADTLRDAGVAF 282
Ec IscS GMRSGTLPVHQIVGMGEAYRIAKEEMA-T----EMERLRGLR----NRLWNGIKDI-EEV 287
At mNif1 GLRSGTGATQQIVGFGAACELAMKEME-Y----DEKWIKGLQ----ERLLNGVREKLDGV 336
Sc Nfs1 GLRSGTLAPPLVAGFGEAARLMKKEFD-N----DQAHIKRLS----DKLVKGLLSA-EHT 380
Pf SufS NLSYKKENINYKSHMQTHPPVHKYNDEQNFTNDHNITQSKQTKSIHSQHDTFKIYTHDTR 458
Bs SufS TVYGPEE--- 327
Ec SufS TLYGPQN--- 330
At cNif1 RIYGPRP--- 378
Pf IscS VFNGCQINR--- 442
Bs YrvO EVNGDKEHS--- 291
Ec IscS YLNGDLEHG--- 296
At mNif1 VVNGSMDSR--- 345
Sc Nfs1 TLNGSPDHR--- 389
Pf SufS KYGLKKIGILPLWSNTFSSFDLVTFLDF-KNICIRAGHHCASLLH--KYYLKV------- 508
Bs SufS -----RAGLVTFNLDDVHPHDVATVLDA-EGIAVRAGHHCAQPLM--KW-LDV------- 371
Ec SufS -----RLGVIAFNLGKHHAYDVGSFLDN-YGIAVRTGHHCAMPLM--AY-YNV------- 374
At cNif1 SESVHRGALCSFNVEGLHPTDLATFLDQQHGVAIRSGHHCAQPLH--RY-LGV------- 428
Pf IscS -----YYGNMNISFLFVEGESLLMSL---NEIALSSGSACTSSTLEPSYVLRSI-GISED 493
Bs YrvO -----LPHVLNLYFPGVSVEALLVNLDM-AGVAVSSGSACTAGSVLPSHVLTAMFGEESD 345
Ec IscS -----APNILNVSFNYVEGESLIMAL---KDLAVSSGSACTSASLEPSYVLRAL-GLNDE 347
At mNif1 -----YVGNLNLSFAYVEGESLLMGL---KEVAVSSGSACTSASLEPSYVLRAL-GVDED 396
Sc Nfs1 -----YPGCVNVSFAYVEGESLLMAL---RDIALSSGSACTSASLEPSYVLHAL-GKDDA 440
Pf SufS --PDTSRISIYFYNTPQEIKYLAQQIASTSFMLNEMKNEK-------------------- 566
Bs SufS --TATARASFYLYNTEEEIDKLVEALQKTKEYFTNVF----------------------- 429
Ec SufS --PAMCRASLAMYNTHEEVDRLVTGLQRIHRLLG-------------------------- 432
At cNif1 --NASARASLYFYNTKDDVDAFIVALADTVSFFNSFK----------------------- 486
Pf IscS IAHTSIRIGFNRFTTFFEVQQLCINLVKSVERLRSISPLYEMELEKKNPSNDDIPKFIWT 553
Bs YrvO RLTSSIRISFGLGNTAEQVKTAAKHVADVVKRLT-------------------------- 405
Ec IscS LAHSSIRFSLGRFTTEEEIDYTIELVRKSIGRLRDLSPLWEMYKQGV-----DLNSIEWA 402
At mNif1 MAHTSIRFGIGRFTTKEEIDKAVELTVKQVEKLREMSPLYEMVKEGI-----DIKNIQWS 451
Sc Nfs1 LAHSSIRFGIGRFSTEEEVDYVVKAVSDRVKFLRELSPLWEMVQEGI-----DLNSIKWS 495
Pf SufS -- 546
Bs SufS -- 406
Ec SufS -- 406
At cNif1 -- 463
Pf IscS -- 553
Bs YrvO -- 379
Ec IscS HH 404
At mNif1 QH 453
Sc Nfs1 GH 497
Class II
Class I

B

**Percentage (%) sequence identity among the cysteine desulfurases**									
	***Pf* SufS**	*Bs* SufS	*Ec* SufS	*At* cNif1	*Pf* IscS	*Bs* YrvO	*Ec* IscS	*At* mNif1	*Sc* Nfs1
***Pf* SufS**		**45.57**	**41.53**	**40.98**	**28.41**	**33.89**	**34.37**	**29.62**	**29.73**
Bs SufS	**45.57**		67.08	67.23	36.13	42.68	39.95	38.21	38.55
Ec SufS	**41.53**	67.08		63.11	35.66	38.86	36.95	36.56	35.75
At cNif1	**40.98**	67.23	63.11		30.24	38.82	37.62	36.65	34.31
Pf IscS	**28.41**	36.13	35.66	30.24		58.91	69.25	54.95	55.94
Bs YrvO	**33.89**	42.68	38.86	38.82	58.91		62.76	59.42	60.47
Ec IscS	**34.37**	39.95	36.95	37.62	69.25	62.76		76.30	73.51
At mNif1	**29.62**	38.21	36.56	36.65	54.95	59.42	76.30		66.87
Sc Nfs1	**29.73**	38.55	35.75	34.31	55.94	60.47	73.51	66.87	

Figure 7- figure supplement 1- source data 1. (A) Multiple sequence alignment used for **Figure 7- figure supplement 1**. **(B)** Table showing the percentage sequence identity among cysteine desulfurase orthologs based on the sequence alignment shown in panel (A).
