# Supplementary material for: The Plasmodium falciparum apicoplast cysteine desulfurase provides sulfur for both iron-sulfur cluster assembly and tRNA modification
Source: eLife. 2023 May 11;12:e84491. doi: 10.7554/eLife.84491 (PMC10219651; doi:10.7554/eLife.84491)
Supplement: Supplementary file 1. — (a) Sulfur-dependent pathways present in P. falciparum. (b) Summary of attempted transfections in P. falciparum to knockout genes of interest. (c) Plasmids used for generation of gene deletion lines. (d) Primers used in this study. Restriction enzyme sites are single underlined, linker regions are double underlined. [file elife-84491-supp1.docx]

**Supplementary file 1a. Sulfur-dependent pathways present in *P. falciparum*.**

*Although thiamine biosynthesis pathway is present in *P. falciparum*, the parasite depends on exogenous supplementation from the host (1), ^#^ From genome wide saturation piggyBac transposon mutagenesis study (2).

| Pathways that require sulfur | Presence in asexual blood stage | Essentiality in asexual blood stage | Localization | Reference |
| --- | --- | --- | --- | --- |
| FeS assembly | Present | Essential | Apicoplast and mitochondria | (3, 4) |
| Lipoic acid biosynthesis | Present | Non-essential | Apicoplast | (5, 6) |
| Biotin biosynthesis | Absent |  |  | (7, 8) |
| Molybdopterin biosynthesis | Absent |  |  | (7, 9) |
| Selenocysteine biosynthesis | Present | Non-essential^#^ | Cytosol | (10, 11) |
| Thiamine biosynthesis* | Present | Essential | Cytosol | (1, 12, 13) |
| Thio-modification of tRNA | Present | Essential^#^ | Apicoplast | (14) |

**Supplementary file 1b. Summary of attempted transfections in *P. falciparum* to knockout genes of interest**

| Parental line | Gene of interest | No. of attempted independent transfection | No. of transgenic parasite lines with successful deletion |
| --- | --- | --- | --- |
| PfMev | *sufC* | 8 | 5 |
|  | *sufD* | 8 | 5 |
|  | *sufE* | 3 | 3 |
|  | *sufS* | 10 | 8 |
|  | *mnmA* | 3 | 3 |
| ∆*sufD* | *sufC* | 4 | 4 |
| *bsmnmA*^+^ | *mnmA* | 4 | 1 |
| *bsmnmA*-*yrvO*^+^ | *mnmA* | 4 | 4 |
|  | *sufS* | 2 | 2 |

**Supplementary file 1c. Plasmids used for generation of gene deletion lines**

| Gene deletion lines | Target gene | Cas9 plasmid | Repair plasmid |
| --- | --- | --- | --- |
| ∆*sufC* | *sufC* | pCasG- *sufC* | pRSng-*sufC* |
| ∆*sufD* | *sufD* | pCasG-*sufD* | pRSng-*sufD* |
| ∆*sufC/sufD* | *sufC*  *sufD* | pCasG-*sufC*  pCasG-*sufD* | pRSng-(BSD)-*sufC*  pRSng-*sufD* |
| ∆*sufE* | *sufE* | pCasG-*sufE* | pRSng-*sufE* |
| ∆*sufS* | *sufS* | pUF1-Cas9 | pRS-*sufS* |
| ∆*mnmA* | *mnmA* | pCasG-*mnmA* | pRSng-*mnmA* |
| *bsmnmA^+^* Δ*mnmA* | *mnmA* | pCasG-*mnmA* | pRSng-*mnmA* |
| *bsmnmA-yrvO^+^* Δ*mnmA* | *mnmA* | pCasG-*mnmA* | pRSng-*mnmA* |
| *bsmnmA-yrvO^+^* Δ*mnmA* | *sufS* | pUF1-Cas9 | pRS-*sufS* |

**Supplementary file 1d. Primers used in this study.** Restriction enzyme sites are single underlined, linker regions are double underlined.

| Primer name | Sequence (5’🡪3’) |
| --- | --- |
| Primers to amplify homology arms for genetic knockouts | |
| SufC.HA1F | GCCACGAGCGGCCTATGCATCCTTTTGAAACCCTTCAACT |
| SufC.HA1R | AAGCGCAGCGGCCTTGTTATTTTCCATATGCACGACATTC |
| SufC.HA2F | CGACAGACGCCGGAGGAGAAAAAAAAAGAAATGAAATATT |
| SufC. HA2R | GGCCACCAGCCGGCTATTCCTTAAGAAATTGAGAGTACCCC |
| SufD.HA1F | GCCACGAGCGGCCTTTGATTATGTTGAGAGTAAACATTGG |
| SufD.HA1R | AAGCGCAGCGGCCAAATGGGTAGCATTATTAATCTTTAGA |
| SufD.HA2F | CGACAGACGCCGGATGGATTCTCAAGAATTTGTTTAGAAG |
| SufD.HA2R | GGCCACCAGCCGGGTGATTTGCACATTCTATATCACTTGG |
| SufE.HA1F | GCCACGAGCGGCCTTGTTTTCGGGCATACCATTTAATATGC |
| SufE.HA1R | AAGCGCAGCGGCCTGGAGATTACTGAAAAATAAATTGGAGTCA |
| SufE.HA2F | CGACAGACGCCGGCAGGGGTGTCAATCGGTAGTATATATTTAC |
| SufE.HA2R | GGCCACCAGCCGGTGTCCATATTCTTCAATATATTGGTGCAGA |
| SufS.HA1F | GCCACGAGCGGCGCTTAATAAATATTTTCTATTATGTCAT |
| SufS.HA1R | AAGCGCAGCGGCATTATATTATTTCTTCATTTTATTACC |
| SufS.HA2F | CGACAGACGCCGGTAGTAATATAATAACTAATGTTTCTAA |
| SufS.HA2R | GGCCACCAGCCGGTGTGTTATAAAAATATATGGATATCC |
| MnmA.HA1F | GCCACGAGCGGCCATCATTTTGTAAATCTATTAAGGTCACACA |
| MnmA.HA1R | AAGCGCAGCGGCCTATAGCCATAAATAAAATTTCATCACACC |
| MnmA.HA2F | CGACAGACGCCGGAGGGGAAATTACCTGCATGTATAAAAAAG |
| MnmA.HA2R | GGCCACCAGCCGGCAAGAAATTGATTACTTATTGTTGCTGAAC |
| Primers for gRNA to generate knockout lines | |
| SufC.gRNA.F | TAAGTATATAATATTGTTAATTATGGATTTAGTGGGTTTTAGAGCTAGAA |
| SufC.gRNA.R | TTCTAGCTCTAAAACCCACTAAATCCATAATTAACAATATTATATACTTA |
| SufD.gRNA.F | TAAGTATATAATATTAGTAATAGTGGACTAGTTAAGTTTTAGAGCTAGAA |
| SufD.gRNA.R | TTCTAGCTCTAAAACTTAACTAGTCCACTATTACTAATATTATATACTTA |
| SufE.gRNA.F | TAAGTATATAATATTATATAATCTACACATAACAGGTTTTAGAGCTAGAA |
| SufE.gRNA.R | TTCTAGCTCTAAAACCTGTTATGTGTAGATTATATAATATTATATACTTA |
| SufS.gRNA.F | TAAGTATATAATATTAAATTTAAACCATTGTTATAGTTTTAGAGCTAGAA |
| SufS.gRNA.R | TTCTAGCTCTAAAACTATAACAATGGTTTAAATTTAATATTATATACTTA |
| MnmA.gRNA.F | TAAGTATATAATATTACAATATAAAATGGATAGAGGTTTTAGAGCTAGAA |
| MnmA.gRNA.R | TTCTAGCTCTAAAACCTCTATCCATTTTATATTGTAATATTATATACTTA |
| Primers for gene knockout confirmation | |
| SufC.5F | GGGATTTGTCATACATATAAATATGTATAAAAAGGTCC |
| SufC.3R | TAAGTACAAAATAATATCATATGTACATTATAAAATCCAC |
| SufC.5WTR | CTTCTTCTAAATTTTTTAATAAAGATAATCTATCCATATC |
| SufC.3WTF | GAGATAAGTGTAAGTGAATTTAATTTAATGATGATAGAAG |
| SufD.5F | GTGTATATAATTTTTTGAAAAAAATGATTTAGCTAACACA |
| SufD.3R | CAGATATTCCTCTTGTCATTAATGAGAAGATTGG |
| SufD.5WTR | GTGTTTTCTCTTCATTTTGATGTCTATAGTTTTTTATAC |
| SufD.3WTF | CCAATAACAAATCCTAGATTAGTTGTATATGTAAAAGG |
| SufE.5F | CATTTCATTCATTTTGGTAGTTAAAAAGAAAAGG |
| SufE.3R | TCATTTTATTAAAAACATATTTCATTTAATTTGTAAGA |
| SufE.5WTR | CATGAGCATATTATATATAAAACAAAGAAATGTGC |
| SufE.3WTF | CCTGATAAACATAAAATTAGACAAAATCAAGTTTTGG |
| SufS.5F | TTTTCAGAAAATGAGTGAGTTTTATAAAAAGGAAAACTCGA |
| SufS.3R | CAATTTGCTGTGCCAAATATTTGATTTC |
| SufS.5WTR | CAATTTGCTGTGCCAAATATTTGATTTCCTG |
| SufS.3WTF | CCAGATATATTAATAACATCTGGTCATAAATTTTG |
| MnmA.5F | CTACTTTGATGTTTTTTTATTTTTGCACATTTTATG |
| MnmA.3R | GGTATTTTTTATTTGCTCATGTTTAAATCATTTATCTG |
| MnmA.5WTR | CTTTATACTATTGTTTCTTTGTTCGTCTTCATCAATTTG |
| MnmA.3WTF | CTTACTAAAAATTATGACCAAGATTTATTTACACATATACG |
| pRS.R | TACAAAATGCTTAAGCGCAGCGGCC |
| pRS.F | CATATTTATTAAATCTAGAATTCGACAGACGCCG |
| Primers to amplify representative genes from nuclear and organellar genome | |
| LDH.F | GGAGATGTAGTTTTGTTCGATATTG |
| LDH.R | CTTGTAAAGGGATACCACCTACAG |
| SufB.F | CATGTAGCTATAGTAGAAATAATAGTAAAAGATTATGG |
| SufB.R | GACTCTGAAATACTTAAACCACGTTGC |
| Cox1.F | CTTCATCTTTAAGAATAATTGCACAAGAAAATGTAAATC |
| Cox1.R | GTACATATGATGTACCCATACTAAGCTTCC |
| Primers for generation of pCLD-*bsmnmA*-mCherry-10xapt and pCLD-*bsmnmA*-*yrvO*-mCherry-10xapt plasmids | |
| MnmA.BspEI.InF.F | GTTAGAAGGTTCCGGAATGGAAAAACGGCCGGAGG |
| MnmA.BsiWI.InF.R | gcccttgctCgtacgTACGTACCACAATTTTGTTCCGTCTTTG |
| MnmA.Link.R | CCGTTCCATaccagatccactacCTACGTACCACAATTTTGTTCCGTCTTTG |
| YrvO.Link.F | GGTACGTAGgtagtggatctggtATGGAACGGATTTATTTAGATCATGCCG |
| YrvO.BsiWI.InF.R | gcccttgctCgtacgTGTCAGCCGTTTGACAACGTCG |
| MY Apt PspOMI F | tatgcaTAAgggcccactgtCtggcaag |
| MY Apt XmaI R | gacCTTAAGcccgggtcagtcagagcag |
| Primers for generation of pKD-*mnmA*-2xFLAG-10xapt | |
| FLAG.F | GATGTGTCAAGACGTCGATTATAAAGATCATGATGGTGATTATAAGGATCATGATATAGA |
| FLAG.R | CCAGACAGTGGGCCCTTATTTATCATCATCATCCTTATAATCTATATCATGATCCTTATA |
| MnmAKD.HA1F | GATATCgtccacctgGATATCagtcaaaaatattccccatgatga |
| MnmAKD.HA1R | CTTTATAATCgacgtctttgctcatgtttaaatcatttatctg |
| MnmAKD.HA2F | Ccctttccgggcgcgccatatggttgtaatacattttgtatcct |
| MnmAKD.HA2R | GATATCcaggtggacGATATCtatcgtgaataagatatttctggaaatt |
| Primers for gRNA to generate *mnmA*-flag | |
| MnmAKD.gRNA.F | TAAGTATATAATATTtatttgtatgtgtacttatatttGTTTTAGAGCTAGAA |
| MnmAKD.gRNA.R | TTCTAGCTCTAAAACaaatataagtacacatacaaataAATATTATATACTTA |
| Primers for confirmation of *mnmA*-flag genotype | |
| MnmAKD.5F | TTCATGTTTATAATAATATTCAAGAGAGTGG |
| MnmAKD.3R | TGATATGAACATATATAAAGGAGTTTGTAC |
| NewApt.5R | ctcgcTATCAAGGAATCgagtcc |
| HSP86KD.5F | GGAATACTAAATATATATCCAATGGCC |
| Primers for confirmation of gene knock-in | |
| attB.Int.F | GCAGTGTGGAATTCCCTGCA |
| attB.Int.R | TTAAGTGTAGTTAATTCATCAAATAGCATGC |
| P230p.out.HA.F | Ggttgtgatttttcaggtgattcc |
| P230p.out.HA.R | gaaaattgtaggggcagctaaatccgac​ |
| Primers for plasmid sequencing | |
| pRS.R | TACAAAATGCTTAAGCGCAGCGGCC |
| pRS.F | CATATTTATTAAATCTAGAATTCGACAGACGCCG |
| MnmA.seqF | GCTGCGCGGCATTGATG |
| MnmA.seqR | CGCCGATCGGGAACATGAC |
| Primers for *P. falciparum* SufS PCR amplification for sequencing | |
| SufS.SeqF1 | ctATGTTAAGAGGCCCTAGATG |
| SufS.SeqR3 | CGAGTTTTCCTTTTTATAAAACTCACTCA |
| SufS.SeqF3 | TGAGTGAGTTTTATAAAAAGGAAAACTCG |
| SufS.SeqR4 | CCAGGAATATTTTGTGTTCCTGTTTC |
| SufS.SeqF2 | TCATTCAAATATTATTCCCTGGCAAG |
| SufS.SeqR2 | tTCATTTTTCATTTTTCATTTCATTTAACAT |
| Primers for *P. falciparum* SufS amplicon sequencing | |
| SufS.SeqF1 | ctATGTTAAGAGGCCCTAGATG |
| SufS.SeqR1 | CCAATAACATTAGATGCATGACATATAG |
| SufS.SeqF2 | TCATTCAAATATTATTCCCTGGCAAG |
| SufS.SeqR2 | tTCATTTTTCATTTTTCATTTCATTTAACAT |
| SufS.SeqF3 | TGAGTGAGTTTTATAAAAAGGAAAACTCG |
| SufS.SeqR3 | CGAGTTTTCCTTTTTATAAAACTCACTCA |
| SufS.SeqF4 | GAAACAGGAACACAAAATATTCCTGG |
| SufS.SeqR4 | CCAGGAATATTTTGTGTTCCTGTTTC |

**Supplementary references**

1. X. W. Chan *et al.*, Chemical and genetic validation of thiamine utilization as an antimalarial drug target. *Nat Commun* **4**, 2060 (2013).

2. M. Zhang *et al.*, Uncovering the essential genes of the human malaria parasite *Plasmodium falciparum* by saturation mutagenesis. *Science* **360**, eaap7847 (2018).

3. T. A. Dellibovi-Ragheb, J. E. Gisselberg, S. T. Prigge, Parasites FeS up: iron-sulfur cluster biogenesis in eukaryotic pathogens. *PLoS Pathog* **9**, e1003227 (2013).

4. J. E. Gisselberg, T. A. Dellibovi-Ragheb, K. A. Matthews, G. Bosch, S. T. Prigge, The suf iron-sulfur cluster synthesis pathway is required for apicoplast maintenance in malaria parasites. *PLoS Pathog* **9**, e1003655 (2013).

5. J. Storm, S. Müller, Lipoic acid metabolism of *Plasmodium*-a suitable drug target. *Curr Pharm Des* **18**, 3480-3489 (2012).

6. S. Günther *et al.*, Apicoplast lipoic acid protein ligase B is not essential for *Plasmodium falciparum*. *PLoS Pathog* **3**, e189 (2007).

7. A. Krishnan, J. Kloehn, M. Lunghi, D. Soldati-Favre, Vitamin and cofactor acquisition in apicomplexans: synthesis versus salvage. *J Biol Chem* **295**, 701-714 (2020).

8. T. A. Dellibovi-Ragheb *et al.*, Host biotin is required for liver stage development in malaria parasites. *Proc Natl Acad Sci* **115**, E2604-E2613 (2018).

9. E. Kemen *et al.*, Gene gain and loss during evolution of obligate parasitism in the white rust pathogen of *Arabidopsis thaliana*. *PLoS Biol* **9**, e1001094 (2011).

10. A. V. Lobanov *et al.*, The *Plasmodium* selenoproteome. *Nucleic Acids Res* **34**, 496-505 (2006).

11. A. Röseler *et al.*, Insight into the selenoproteome of the malaria parasite *Plasmodium falciparum*. *Antioxid Redox Signal* **17**, 534-543 (2012).

12. M. L. Eschbach, I. B. Muller, T. W. Gilberger, R. D. Walter, C. Wrenger, The human malaria parasite *Plasmodium falciparum* expresses an atypical N-terminally extended pyrophosphokinase with specificity for thiamine. *Biol Chem* **387**, 1583-1591 (2006).

13. C. Wrenger *et al.*, Vitamin B1 de novo synthesis in the human malaria parasite *Plasmodium falciparum* depends on external provision of 4-amino-5-hydroxymethyl-2-methylpyrimidine. *Biol Chem* **387**, 41-51 (2006).

14. S. A. Ralph *et al.*, Metabolic maps and functions of the *Plasmodium falciparum* apicoplast. *Nat Rev Microbiol* **2**, 203-216 (2004).
